# Neuroinflammation and ALS: Transcriptomic Insights into Molecular Disease Mechanisms and Therapeutic Targets

**DOI:** 10.1155/2017/7070469

**Published:** 2017-09-07

**Authors:** Giovanna Morello, Antonio Gianmaria Spampinato, Sebastiano Cavallaro

**Affiliations:** Institute of Neurological Sciences, Italian National Research Council, Catania, Italy

## Abstract

Amyotrophic lateral sclerosis (ALS) is a neurodegenerative disease affecting the motor nervous system. Despite the mechanism underlying motor neuron death is not yet clarified, multiple pathogenic processes have been proposed to account for ALS. Among these, inflammatory/immune responses have recently gained particular interest, although there are conflicting reports on the role of these processes in ALS pathogenesis and treatment. This apparent discrepancy may be due to the absence of an effective stratification of ALS patients into subgroups with markedly different clinical, biological, and molecular features. Our research group recently described genome-wide characterization of motor cortex samples from sporadic ALS (SALS) patients, revealing the existence of molecular and functional heterogeneity in SALS. Here, we reexamine data coming from our previous work, focusing on transcriptomic changes of inflammatory-related genes, in order to investigate their potential contribution in ALS. A total of 1573 inflammatory genes were identified as differentially expressed between SALS patients and controls, characterizing distinct topological pathways and networks, suggestive of specific inflammatory molecular signatures for different patient subgroups. Besides providing promising insights into the intricate relationship between inflammation and ALS, this paper represents a starting point for the rationale design and development of novel and more effective diagnostic and therapeutic applications.

## 1. Introduction

Amyotrophic lateral sclerosis (ALS), or Lou Gehrig's disease, is a fatal neurodegenerative disease characterized by progressive and relatively selective degeneration of the upper and lower motor neurons in the brainstem, spinal cord, and motor cortex, resulting in paralysis and death usually within 3–5 years of diagnosis [1]. With an estimated incidence of about 1–3 cases per 100,000 person-years and a projected lifetime risk of 1/2000, ALS is considered one of the most common motor neuron diseases [2]. ALS is essentially a sporadic disorder (SALS), with 90–95% of the cases originating from an unknown cause, likely resulting from a complex gene-gene and gene-environment interplay [3–6]. The remaining 5–10% of the cases are considered familial (FALS) generally following an autosomal dominant inheritance pattern [7] due to mutations in a number of seemingly disparate genes, including SOD1 [8], ALS2 [9], SETX [10, 11], SPG11 [12], FUS [13, 14], VAPB [15], ANG [16, 17], TARDBP [18–20], FIG4 [21], OPTN [22], ATXN2 [23], and C9ORF72 [24, 25]. Interestingly, mutations in many of these genes were found in both forms, suggesting common disease mechanisms and possibly common triggers [26].

Although the pathophysiological processes and precise genetic causes underlying motor neuron death are still elusive, genomic profiling and expression studies have provided invaluable insights into the molecular mechanisms involved in ALS, supporting a “multiple-hit” hypothesis of neurodegeneration [27–35]. In this regard, a number of converging disease mechanisms are known, including oxidative damage, defective protein misfolding, mitochondrial degeneration, impaired axonal transport, neurotrophic factor deficits, apoptosis, aberrant RNA/DNA regulation, and neuroinflammation [36, 37].

While ALS is not primarily considered an autoimmune or immunodeficiency disease, mounting evidence suggests that immune/inflammatory abnormalities and nonneuronal cells play an important role in the disease onset and progression. Chronically activated microglia and astrocytes as well as infiltrating immune cells represent prominent pathological findings in affected CNS areas of patients and animal models of ALS [38]. In addition, numerous anti- and proinflammatory cytokines and growth factors, including VEGF, IFN-*γ*, TNF-*α*, IL-1*β*, IL-6, and IL-10, seem to play a role in the neuropathological changes that characterize ALS. Nonetheless, it remains to be clarified whether neuroinflammation is a consequence of motor neuron injury or actively contributes to the development and progression of the disease. Indeed, several studies have highlighted the existence of the dual nature of inflammation in ALS, both neuroprotective and neurotoxic, that appears to be mainly dependent on the stage of disease progression. In particular, during periods of slow disease progression, the immune system exerts a protective action by secreting anti-inflammatory factors that rescue and repair damaged tissue. As the disease and motor neuron injury accelerate, a shift occurs from the beneficial immune response to a strong proinflammatory and neurotoxic state [39]. As a consequence, inflammatory mediators have received great attention as potential targets for neuroprotection in ALS, and multiple anti-inflammatory drugs (i.e., minocycline, thalidomide, celecoxib, and pioglitazone) have shown promising results in delaying disease severity in ALS animal models. Unfortunately, none of these compounds have been proved to be effective in clinical testing for ALS [40]. Reasons responsible for this failure include not only the lack of knowledge on the multiple inflammation-related events underlying ALS but also improper planning of clinical trial that does not take clinical, biological, and molecular heterogeneity of the disease into account [41]. Therefore, developing new targeted and stratified treatments that, alone or in combinations, may cope with the multiple inflammatory pathways, continue to be a research priority.

In our previous work, for the first time, we characterized unrecognized molecular heterogeneity in SALS, revealing new clues to the molecular pathogenesis and enabling the identification of novel potential predictive biomarkers and therapeutic targets that were not put in evidence by considering SALS pathology as a single entity [42, 43]. In particular, using a computational analysis of whole genome expression profiles of 41 motor cortex samples of control (10) and SALS patients (31), we were able to differentiate SALS pathology from controls and clearly distinguish the two SALS subtypes (SALS1 and SALS2), each associated with differentially expressed genes and pathways.

In the present paper, we have reexamined our gene expression data and focused on genes and pathways that are related to inflammation. The overall goal was to assess the potential involvement of inflammatory/immunological processes in two molecularly distinct SALS patient subgroups, providing a rationale for the specific use of potential cluster-specific biomarkers and therapeutic targets.

## 2. Materials and Methods

### 2.1. Data Acquisition and Preprocessing

The transcriptomic data were downloaded from the ArrayExpress repository (http://www.ebi.ac.uk/arrayexpress/), with accession number E-MTAB-2325 [42]. This dataset includes transcriptional profiles of 41 postmortem motor cortex samples (31 of which were from patients with SALS and 10 were from control individuals) hybridized on the Whole Human Genome Oligo Expression Microarrays 4x44K (Agilent Technologies). The detailed information regarding clinical, genetic, and phenotypic data of patient samples has been previously described [42]. The original gene expression dataset was normalized to the 50th percentile, followed by the median baseline of all samples using GeneSpring GX v13.1 (Agilent Technologies, Italy). The resulting expression values were thresholded to 1, log2 transformed, and fold changes (FCs) were calculated between the SALS patients and individual controls. Probes not corresponding to an Entrez ID were removed. In cases where several probes corresponded to one Entrez ID, the probe showing the highest variance over all samples was chosen for further analysis.

### 2.2. Gene List Filtering and Differential Expression Analysis

The expression data were filtered to include only probes targeting genes involved in neuroinflammation and immune response. In particular, a set of inflammatory genes was generated using inflammatory/immunology-related query keywords in the Gene Ontology (GO) database (http://www.geneontology.org/) [44]. Overall, 2637 genes were selected from the *Immune system process* (GO: 0002376) biological process term. This initial gene list was subjected to fold-change-based filtering and statistical analysis by using GeneSpring GX v13.1 software package (Agilent Technologies). In particular, we performed a one-way analysis of variance (ANOVA) followed by Tukey's post hoc test to identify differentially expressed genes (DEGs) between the two predefined groups of SALS patients relative to controls. Furthermore, the Benjamini-Hochberg false discovery rate (FDR) correction procedure was used to minimize false-positive cases. An absolute FC value greater than 1.5 and an adjusted *P* value of < 0.05 were used as criteria for defining a set of deregulated candidate genes for further exploration.

### 2.3. Construction and Topological Analysis of Protein-Protein Interaction Network

To better clarify the interaction between immune/inflammatory DEGs and emphasize their potential contribution to ALS pathology, two extended protein-protein interaction (PPI) networks were built by using STRING database v.10.0 and visualized with the Cytoscape v.3.4.0 software [45]. In particular, these extended networks were constructed by using DEGs in both SALS patient subgroups as seed molecules and setting a high level of confidence between molecular interactions (high confidence score of at least 0.8) and a maximum number of interactions to 100. In both networks, nodes correspond to proteins encoded by DEGs in SALS, whereas edges represent the number of interactions between proteins. All interactions in both networks were unweighted and undirected. Once extended networks were constructed, duplicated edges and self-loops were removed. Subsequently, in a prefilter process, we considered only nodes (genes) that were annotated with a high confidence score to the central nervous system by using the TISSUES web resource [46].

In order to identify the “hub” nodes, a network topology analysis was performed by using the Cytoscape plug-in NetworkAnalyzer based on topological parameters [47]. Node (gene) centrality in both networks was also investigated through the evaluation of “node degree.” This topological parameter indicates the relevance of a gene (node) as functionally capable of holding together the communicating nodes in a biological network. Nodes with high degree (hub genes) represented the genes having important biological functions: the higher the value, the higher the relevance of the gene in connecting regulatory molecules. The final PPI networks were visualized based on node degree and edge betweenness parameters. The relative importance of the genes in each network was determined based on the node centrality measure setting the topological parameter “node degree” ≥10. Likewise, values of edge betweenness were mapped with the edge size: high values of this parameter correspond to a large edge size.

### 2.4. Functional Analysis and Selection of the Candidate Pathways

To investigate the relevance of inflammatory-related DEGs in determining specific molecular signatures in SALS pathology, pathway analysis was performed with Ingenuity Pathway Analysis (IPA®; http://www.ingenuity.com/) and GeneGO MetaCore™ [48]. Both these programs identify significantly enriched biological pathways and signaling cascades that are associated with a given list of genes by calculating the hypergeometric distribution. In accordance with the purpose of this study, we focused on predefined the “canonical pathways” commonly associated with immune response/inflammation processes. Finally, immune/inflammatory pathways with a *P* value of < 0.05 and a fold change of >1.5 were screened and analyzed.

In addition, to reduce potential errors due to the use of preselected gene sets [49] and to increase the strength of the functional analysis, we also performed a “control” pathway enrichment analysis both on the entire list of differentially expressed genes in SALS patients versus controls, both without the assignment of SALS patients into the two cluster groups (see Supplementary Tables 1 and 2 available online at https://doi.org/10.1155/2017/7070469).

## 3. Results

### 3.1. Identification of Differentially Expressed Inflammatory Genes

**Figure 1:**
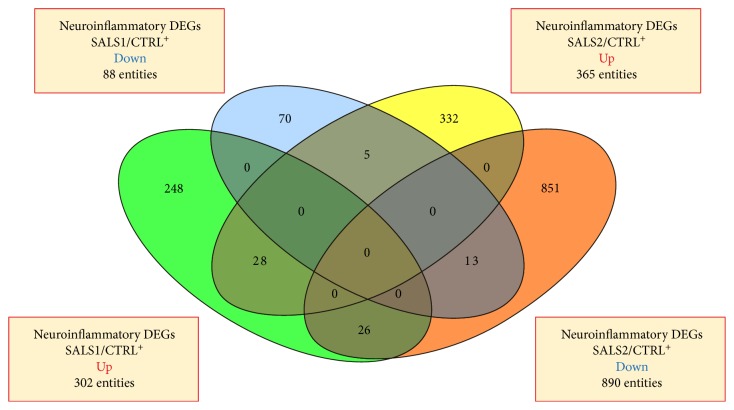
Venn diagrams of the total number of upregulated and downregulated inflammatory genes in the genes of SALS1 and SALS2 versus controls. Detailed information for the lists of genes differentially expressed in SALS1 and SALS2 is provided in Supplementary Tables 3–5.

Our results indicate that 1573 out of 2637 inflammation genes (probes) were differentially expressed between SALS patients and controls. In particular, a total of 390 immune/inflammation genes were found to be significantly differentially expressed in SALS1 (302 upregulated and 88 downregulated), while SALS2 patients showed significant changes of expression in 1255 genes (365 upregulated and 890 downregulated) (Figure 1(); Supplementary Tables 3–5). Although some of these genes (72) were differentially expressed in both pairwise comparisons, the majority of them were cluster-specific (Figure 1()).

### 3.2. Network Analysis and Characterization of Hub Genes

**Figure 2:**
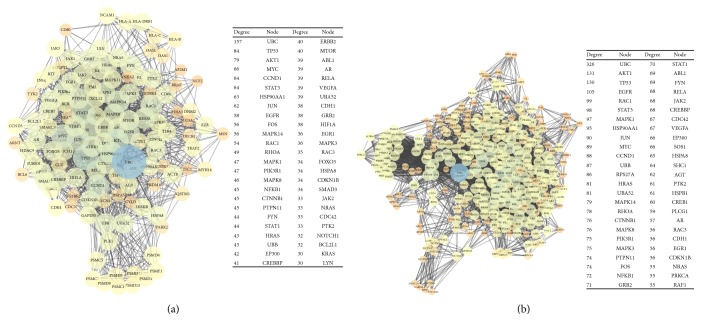
SALS-related PPI networks. (a) Graphical representation of the PPI network from inflammatory DEGs in SALS1. Nodes having a high degree are the ones that form most interactions with other nodes and were displayed as a big circle and dark colors. The right panel shows the top 50 nodes in PPI network order by descending degree value. (b) Graphical representation of the PPI network from inflammatory DEGs in SALS2. Nodes having a high degree are the ones that form most interactions with other nodes and were displayed as a big circle and dark colors. The right panel shows the top 50 nodes in PPI network order by descending degree value.

To gain further insights into the functional significance of inflammatory-related DEGs in SALS and prioritize putative genetic markers that might increase the susceptibility of patients affected by SALS, we mapped these genes into PPI networks for each SALS subgroup. Initially, the extended PPI network for SALS1 contained 448 gene signatures (nodes) with 25,512 interactions (edges), while the extended PPI network associated with SALS2 had 1123 nodes and 8667 interactions. Identifying hub genes as the DEGs with the highest node degree and ranking them for importance based on the node centrality measure reduced the SALS1-PPI network to 126 nodes connected through 1160 edges (with high values of betweenness) and the SALS2-PPI network to 327 nodes connected through 4867 edges (Figure 2() and Supplementary Tables 6 and 7). The information corresponding to the centralities of the top 50 ranked genes in both networks is represented in Figure 2().

Integrated analysis revealed that the two SALS-PPI networks shared similar genetic nodes and interactions. Although both, driving regulators and essential genes, are profoundly different between SALS patient subgroups, they are complementary and converge to similar immune/inflammatory signaling mechanisms within their respective subtypes (Figure 2). In particular, it was observed that *UBC* was the top-ranked gene within PPI networks for both SALS1 and SALS2 patients, with node degrees of 157 and 326, respectively, and thus constituted a superhub gene with a wide variety of cooperative partners. These data indicated that *UBC*, together with other key nodes (hubs) displaying the highest connectivity in both networks, such as *AKT1* and *TP53*, may play a critical role in activating the neuroinflammatory state in SALS patients and thus may represent potential genetic markers with direct or indirect involvement in ALS pathogenesis.

### 3.3. Functional Enrichment Analysis of Inflammatory DEGs in SALS Patient Subgroups

In order to better characterize specific inflammatory molecular signatures for SALS, we investigated whether DE inflammatory genes in SALS patients were enriched for certain specific biological functions and pathways, by using functional ontologies represented in IPA and MetaCore repositories (see Materials and Methods).

**Figure 3:**
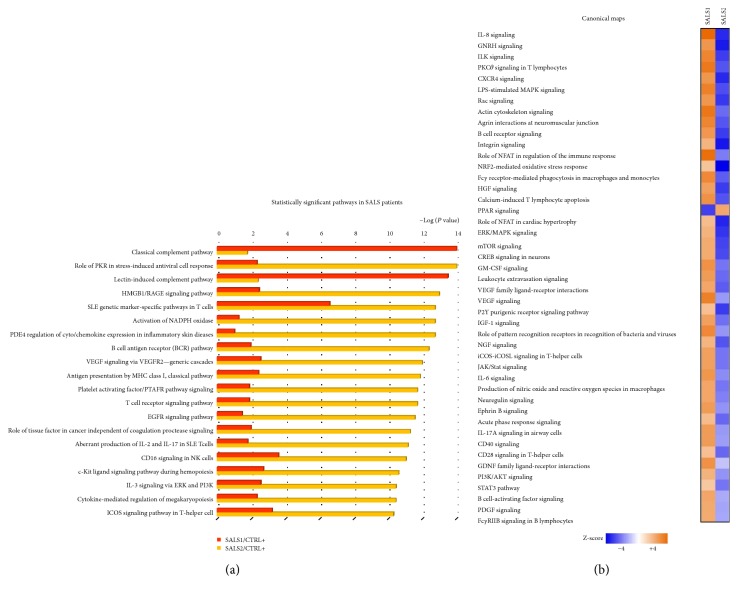
Functional enrichment analysis of inflammatory DEGs in SALS patients. (a) Representation of the top 20 most significantly enriched (*P* value < 0.05) canonical pathway maps associated with the neuroinflammatory DEGs genes in both SALS patient subgroups when compared to controls. A histogram of statistical significance (−log *P* value) is shown: the list is arranged in descending order with the most significant pathways at the top. The analysis was performed using the MetaCore pathway analysis suite. (b) The heat map from IPA of canonical signaling pathways (rows) most significantly enriched in neuroinflammatory genes is differentially expressed in the two SALS patient subgroups (columns). The score magnitudes are shown in a gradient color, from light to bright orange, for induced, and from light to bright blue, for suppressed pathway activity. Detailed information about pathway map enrichment analysis is described in Supplementary Table 8.

Comparison of both SALS patient subtypes with the total control group revealed a total of 585 significantly deregulated pathways, the majority of which were deregulated in the opposite way in the two SALS subtypes (Figure 3 and Supplementary Table 8). In particular, SALS1 was mainly characterized by increased expression of genes involved in the inflammatory response, including *complement system and antigen presentation pathway* (*P* value = 1.082*E*−14), *chemokines and cell adhesion* (*P* value = 2.649*E*−07), and *cytoskeletal remodeling* (*P* value = 9.824*E*−07), as well as a reduced expression of genes associated with the *apoptotic signaling* (*P* value = 1.145*E*−02). SALS2 patients, instead, showed an overall downregulation of inflammatory-related pathways, such as *HMGB1/RAGE signaling pathway* (*P* value = 1.203*E*−13) and *B cell antigen receptor pathway* (*P* value = 5.132*E*−13) as well as alteration of *oxidative stress* (*P* value = 2.133*E*−13). A detailed description of the most significant variations implicated in the inflammatory and immunological pathways affected in SALS is provided in the Supplementary Information section (Supplementary Discussion and Supplementary Figures 1–4).

## 4. Discussion

Inflammation and abnormal or hyperactive immune responses play a pivotal role in the pathogenesis and progression of several neurodegenerative diseases, including ALS. While the molecular basis of neuroinflammation in ALS are being defined, the tools and indicators for early diagnosis and effective treatment options remain incompletely characterized as well as their complex interplay within the major signaling cascades occurring during neuroinflammatory processes. Multiple anti-inflammatory compounds have been evaluated preclinically for their therapeutic potential in ALS showing promising results, but none of these have been proved to be effective in patients [40]. This failure may be mainly due to the absence of an effective stratification of ALS patients into subgroups with markedly different clinical, biological, and molecular features [41].

In the current study, we reanalyzed our previous dataset (E-MTAB-2325), consisting of whole genome expression profiles of 41 motor cortex samples from SALS and control patients, focusing on transcriptomic changes of multiple genes involved in various aspects of inflammation and immune responses, in order to investigate their potential contribution in SALS etiopathogenesis.

Although the use of postmortem brain tissues impedes deeper understanding of the pathophysiological processes ongoing in the diseased brain, they represent a valuable resource for human studies, providing valuable information that cannot be obtained by using other approaches on a living patient.

A total of 1573 inflammatory genes were differentially expressed between SALS patients and controls, the majority of which were cluster-specific, suggestive of a great divergence of the two SALS subgroups at the molecular level (Figure 1).

Components of inflammatory/immune responses are very numerous and interact with each other across multiple functional pathways, impeding the identification of genetic risk factors that effectively contribute to the neuroinflammatory process in ALS. To prioritize the identification of key molecular candidates that could be used for the discovery of therapeutic targets and diagnostic biomarkers, we performed a network analysis on shortlists of inflammatory DEGs in SALS patients (Figure 2). This machine learning-based approach revealed that, although profoundly different, the two subtype-specific SALS networks were complementary and converged to similar immune/inflammatory signaling mechanisms and driving genes, suggesting that in each SALS subtype, there is a “deterministic” path for aberrant immune/inflammatory responses driven by genomic alterations, and the networks could, therefore, provide “predictable” power for selective genomic alterations. In particular, *UBC* was identified as a superhub gene within both SALS-related PPI networks (Figures 2(a) and 2(b)). These findings are in line with the results of a previous network-based genomic analysis on peripheral motor nerves of SALS patients, which revealed a significant overrepresentation of pathways related to ubiquitin-protein ligase activity and identified *UBC* as the most relevant hub gene [50]. The functional significance and contribution of ubiquitin in ALS pathology are also supported by the presence of low expression levels and cytoplasmic inclusions of this protein in the spinal cord motor neurons of ALS patients, supporting its potential role as a biomarker for the disease [51–54]. Despite pharmacologic strategies, aimed to increase or replace the specific lost ubiquitin activity, that have been demonstrated to be effective in preventing abnormal protein accumulation in several human disorders, the clinical use of these treatments has proved to be particularly challenging due to their numerous potential off-target effects [55]. Therefore, there is a need to design therapies that selectively interfere with various components of the ubiquitin-proteasome system, offering new therapeutic perspectives for the treatment of various neurological diseases, including ALS.

To investigate the combined effects of multiple immune/inflammation dysregulations, we have searched for canonical signaling pathways significantly enriched in inflammatory DEGs and mapped them into IPA and MetaCore repositories of signaling pathways (Figures 3(a) and 3(b)). Functional classification of these DEGs showed that *antigen processing and presentation*, *complement system*, and *reactive oxygen species production* are the most significant immune/inflammation pathways deregulated in SALS, suggesting that these processes play a crucial role in the progressive degeneration and loss of motor neurons (Figure 3(a)). Scientific evidence about a pathogenic role for most of these pathways is already available in the literature [41, 56–60]; however, the contribution of the single inflammatory cascade to the distinct SALS subtypes still needs to be clarified. Notably, the majority of the identified signaling cascades were deregulated in the opposite way in the two SALS subtypes, supporting the existence of a specific molecular signature associated with the immune/inflammatory status in SALS patients (Figure 3(b)). In particular, SALS1 patients seem to be associated with an increased inflammatory phenotype while SALS2 patients show reduced expression levels in genes involved in immune response and inflammatory signaling pathways.

Interestingly, the analysis of deregulated inflammatory cascades reveals the involvement of a variety of genes that have been implicated, up to date, in the causation and/or susceptibility of ALS (Supplementary Tables 3 and 4). Among these, one of the striking observations is the differential expression of numerous ALS-linked genes (i.e., ANG, DCTN1, SQSTM1, and TBK1) involved in autophagy, a highly conserved and tightly regulated cellular self-degradative process whose alteration leads to an impaired clearance of toxic protein aggregates and/or of damaged mitochondria that represent some of the best characterized hallmarks of both SALS and FALS [61]. In particular, significantly reduced mRNA levels of TBK1, the most recently identified ALS gene, were observed in SALS2, confirming that reduced activity of this enzyme may result in impaired autophagy and contribute to the accumulation of protein aggregates in motor neurons and ALS pathology [62]. Given the implication that TBK1 plays a key role in ALS and the observation that some autophagy inducers, such as rapamycin, have been already shown to be promising ALS drug candidates, it seems worthwhile to explore TBK1 as a more defined target as well as envisage the use of its pharmacological activators for developing novel and targeted therapeutics for patients.

Other than confirming the role of previously ALS-linked genes, our analysis also identified novel potential candidate genes that deserve further investigation and validation for better establishing their role in ALS pathology. Among these, we distinguish the deregulated expression of a series of molecules implicated in antigen generation and/or trimming, sustaining the involvement of a dysfunction in protein turnover and ubiquitin-proteasome pathways in ALS (Supplementary Figure 1). In particular, overexpression of IMPAS-1 in SALS patients is supported by several studies that correlate high levels of this protease with the aberrant autophagic activity associated with numerous neurodegenerative diseases [63]. All together, these results suggest that deciphering the complex actions of altered protein recycling and degradation machinery networks may help to further elucidate the neuroinflammatory processes occurring in ALS.

## 5. Conclusion

Overall, our findings not only provide interesting insights into the role of inflammatory/immune responses in the pathogenesis of SALS but also underline the existence of molecular heterogeneity in the inflammatory status of different subtypes of SALS patients, providing a rationale for the specific use of potential cluster-specific biomarkers and therapeutic targets. However, it is necessary to take into consideration that deregulation of identified candidate genes in human postmortem tissues may be due to reactive changes that occur in the final stages of disease, impeding to distinguish causative factors from secondary degenerative changes ongoing in the diseased brain. Therefore, future functional and clinical investigation will be necessary to assess the potential role of these candidates in affecting the origins and/or progression of the disease, opening the way to the development of novel and more effective diagnostic, prognostic, and therapeutic applications.

## Supplementary Material

Supplementary Figure 1. Alterations in the antigen processing and presentation pathway associated with SALS patients. This figure illustrates genes differentially expressed in SALS patients that are involved in the antigen processing and presentation process. Each encoded protein is labeled with two thermometers (1) or (2) that indicate expression levels in SALS cluster 1 and 2, respectively. Upward thermometers have red color and indicate up-regulated signals in SALS patients, down-ward (blue) ones indicate down-regulated signals. Colored hexagons on the vectors between objects describe the type of interaction where B = binding, C = cleavage, CM = covalent modification, Cn = competition, CS = complex subunit, GR = group relation, IE = influence on expression, P = phosphorylation, T = transformation, TR = transcriptional regulation, Tn = transport and CR indicates that an object belongs to a group of related proteins. Lines indicate activation (green), inhibition (red) or unspecified (grey) interactions between the molecules. The object shapes correspond to molecule type and are described in the Supplementary Figure 5. Supplementary Figure 2. Alterations in immune and inflammatory signaling observed in SALS patients. Genes involved in immune and inflammatory signaling that were differentially expressed in SALS patients versus controls were mapped on pathway. Thermometers labeled with (1) or (2) indicate expression levels in SALS cluster 1 and 2, respectively. Up-ward thermometers have red color and indicate up-regulated signals in SALS patients, down-ward (blue) ones indicate down-regulated signals. Colored hexagons on the vectors between objects describe the type of interaction where B = binding, C = cleavage, CM = covalent modification, Cn = competition, CS = complex subunit, GR = group relation, IE = influence on expression, P = phosphorylation, T = transformation, TR = transcriptional regulation, Tn = transport and CR indicates that an object belongs to a group of related proteins. Lines indicate activation (green), inhibition (red) or unspecified (grey) interactions between the molecules. The object shapes correspond to molecule type and are described in the Supplementary Figure 5. Supplementary Figure 3. Alterations in complement system signaling pathways (classical, lectin and alternative) observed in SALS patients. Thermometers labeled with (1) or (2) indicate expression levels in SALS cluster 1 and 2, respectively. Up-ward thermometers have red color and indicate up-regulated signals in SALS patients, down-ward (blue) ones indicate down-regulated signals. Colored hexagons on the vectors between objects describe the type of interaction where B = binding, C = cleavage, CM = covalent modification, Cn = competition, CS = complex subunit, GR = group relation, IE = influence on expression, P = phosphorylation, T = transformation, TR = transcriptional regulation, Tn = transport and CR indicates that an object belongs to a group of related proteins. Lines indicate activation (green), inhibition (red) or unspecified (grey) interactions between the molecules. The object shapes correspond to molecule type and are described in the Supplementary Figure 5. Supplementary Figure 4. Alterations in cytokine signaling observed in SALS patients. Thermometers labeled with (1) or (2) indicate expression levels in SALS cluster 1 and 2, respectively. Up-ward thermometers have red color and indicate up-regulated signals in SALS patients, down-ward (blue) ones indicate down-regulated signals. Colored hexagons on the vectors between objects describe the type of interaction where B = binding, C = cleavage, CM = covalent modification, Cn = competition, CS = complex subunit, GR = group relation, IE = influence on expression, P = phosphorylation, T = transformation, TR = transcriptional regulation, Tn = transport and CR indicates that an object belongs to a group of related proteins. Lines indicate activation (green), inhibition (red) or unspecified (grey) interactions between the molecules. The object shapes correspond to molecule type and are described in the Supplementary Figure 5. Supplementary Figure 5. Legend describing symbols used in MetaCore pathway. Supplementary Table 1. Pathway enrichment analysis on the entire list of statistically deregulated genes in SALS patients versus controls. Supplementary Table 2. Pathway enrichment analysis on the entire list of statistically deregulated genes in SALS1 and SALS2 patients compared to controls. Supplementary Table 3. Neuroinflammatory genes differentially expressed in SALS1. Supplementary Table 4. Neuroinflammatory genes differentially expressed in SALS2. Supplementary Table 5. Neuroinflammatory genes differentially expressed in SALS1 and SALS2. Supplementary Table 6. List of genes included in the SALS1-related PPI network. Supplementary Table 7. List of genes included in the SALS2-related PPI network. Supplementary Table 8. Pathway enrichment analyses on the list of statistically deregulated neuroinflammatory genes in SALS patients.

## Abbreviations

SOD1: Superoxide dismutase 1
ALS2: Alsin
SETX: Senataxin
SPG11: Spastic paraplegia 11
FUS: Fused in sarcoma RNA binding protein
VAMP: Vesicle-associated membrane protein
VAPB: Associated protein B and C
ANG: Angiogenin
TARDBP: TAR DNA-binding protein
FIG4: FIG4 phosphoinositide 5-phosphatase
OPTN: Optineurin
ATXN2: Ataxin-2
C9ORF72: Chromosome 9 open reading frame 72
VEGF: Vascular endothelial growth factor
IFN-*γ*: Interferon gamma
TNF-*α*: Tumor necrosis factor
ILs: Interleukins
IL-1*β*: Interleukin 1 beta
UBC: Ubiquitin C
TBK1: TANK-binding kinase 1
SQSTM1: Sequestosome 1
DCTN1: Dynactin subunit 1
IMPAS-1: Histocompatibility minor 13.
