# Supplementary material for: Neuroinflammation and ALS: Transcriptomic Insights into Molecular Disease Mechanisms and Therapeutic Targets
Source: Mediators Inflamm. 2017 Sep 7;2017:7070469. doi: 10.1155/2017/7070469 (PMC5610842; doi:10.1155/2017/7070469)
Supplement: Supplementary file 1 — Supplementary Figure 1. Alterations in the antigen processing and presentation pathway associated with SALS patients. This figure illustrates genes differentially expressed in SALS patients that are involved in the antigen processing and presentation process. Each encoded protein is labeled with two thermometers (1) or (2) that indicate expression levels in SALS cluster 1 and 2, respectively. Upward thermometers have red color and indicate up-regulated signals in SALS patients, down-ward (blue) ones indicate down-regulated signals. Colored hexagons on the vectors between objects describe the type of interaction where B = binding, C = cleavage, CM = covalent modification, Cn = competition, CS = complex subunit, GR = group relation, IE = influence on expression, P = phosphorylation, T = transformation, TR = transcriptional regulation, Tn = transport and CR indicates that an object belongs to a group of related proteins. Lines indicate activation (green), inhibition (red) or unspecified (grey) interactions between the molecules. The object shapes correspond to molecule type and are described in the Supplementary Figure 5. Supplementary Figure 2. Alterations in immune and inflammatory signaling observed in SALS patients. Genes involved in immune and inflammatory signaling that were differentially expressed in SALS patients versus controls were mapped on pathway. Thermometers labeled with (1) or (2) indicate expression levels in SALS cluster 1 and 2, respectively. Up-ward thermometers have red color and indicate up-regulated signals in SALS patients, down-ward (blue) ones indicate down-regulated signals. Colored hexagons on the vectors between objects describe the type of interaction where B = binding, C = cleavage, CM = covalent modification, Cn = competition, CS = complex subunit, GR = group relation, IE = influence on expression, P = phosphorylation, T = transformation, TR = transcriptional regulation, Tn = transport and CR indicates that an object belongs to a group [file 7070469.f1.pdf]

Original Article *Mediators of Inflammation*

Special Issue: ***Mediators of Inflammation in Amyotrophic Lateral Sclerosis***

Neuroinflammation and ALS: Transcriptomic Insights into Molecular Disease Mechanisms and Therapeutic Targets

Giovanna Morello[a], Antonio Gianmaria Spampinato[a] and Sebastiano Cavallaro[a, *]

[a] *Institute of Neurological Sciences, Italian National Research Council, Catania, Italy.*

[*]Corresponding author: Sebastiano Cavallaro, M.D., Ph.D., Institute of Neurological Sciences (ISN), CNR, Via Paolo Gaifami, 18, 95125 Catania, Italy. Phone: +39.095.7338111; Fax: +39.095.7338110; E-mail: sebastiano.cavallaro@cnr.it

Supplementary Information

Supplementary Discussion

In the following paragraphs, we will provide a detailed analysis of inflammatory and immunological pathways affected in SALS, focusing attention on potential biomarkers and pharmacological targets for the development of more efficacious and individualized therapeutic interventions for ALS patients.

Antigen processing and presentation

Antigen processing and presentation is the process by which antigen-presenting cells (APCs), such as dendritic cells, macrophages and B cells, express antigen on their cell surface in a form recognizable by T lymphocytes and other immune-related cells. Previous evidence showed that dysregulation of genes related to the antigen-processing machinery seems to account for a number of intercellular mechanisms that are able to amplify the harmful non-autonomous cell toxicity in ALS animal models [1]. Consistent with this scenario, we observed deregulated expression of several genes encoding proteins involved in antigen processing and presentation as well as in the activation/modulation of adaptive immunity, mainly in SALS2 patients (Supplementary Figure 1).

Among these genes, we distinguish those involved in the formation and regulation of the proteasome system, the main intracellular proteolytic mechanism controlling protein turnover and the selective degradation of misfolded/abnormal proteins (Supplementary Figure 1) [2]. The accumulation of abnormal protein aggregates into damaged neurons represents a common hallmark in multiple neurodegenerative diseases, including ALS, and altered expression of multiple proteasome subunits was widely documented in both patients and animal models of ALS [3, 4]. In addition, other proteases implicated in antigen generation and/or trimming (TPP2, NPEPPS, BLMH, NRD1, THOP1, SPC, PC7 and IMPAS-1), were found deregulated in SALS patients, sustaining the involvement of a dysfunction in protein turnover and ubiquitin–proteasome pathways

in ALS (Supplementary Figure 1). In particular, NPEPPS has been recently identified as a major peptidase acting on neurotoxic protein substrates, including SOD1, and its expression was found significantly decreased in motor neurons of both ALS transgenic mice and patients, supporting its role in the disease pathogenesis [5]. Low expression levels of the gene encoding nardilysin convertase (*NRD1*) in SALS2 patients (Supplementary Figure 1) are in line with the evidence that the loss of this metalloendopeptidase in neurons leads to impaired motor activities and cognitive deficits by altering axonal maturation and myelination in the CNS [6]. Overexpression of *IMPAS-1* in SALS patients is supported by several studies that correlate high levels of this protease with the aberrant autophagic activity associated with numerous neurodegenerative diseases [7].

Several molecular chaperones have been already extensively implicated in the pathogenesis of ALS, playing an essential role in the folding and maturation of proteins [8]. Consistent with our results, deregulated levels of HSP70, HSP90 and their interacting protein CHIP were previously detected in the serum of FALS patients, suggesting that deregulation of this protein system may reflect the degeneration of motor neurons in both forms of the disease [9]. Interestingly, recent studies have reported that treatments with arimoclomol, a strong co-inducer of HSPs, significantly delays disease progression in ALS animal models, supporting it as a potentially efficacious therapy for ALS.

Antigen presentation is mediated by the major histocompatibility complex (MHC) class I and class II molecules that are responsible to deliver short peptides to the APC surface for recognition by CD8+ (cytotoxic) and CD4+ (helper) T cells, respectively. The main difference between these molecules is that the MHC class I pathway is usually fueled by endogenous antigen, while exogenous peptides reach the MHC class II pathway. SALS patients showed deregulated expression of genes encoding MHC class I and II molecules and different components of the peptide-loading complex (TAP1, TAP2, TPSN, PDIA3, CALR, CANX, UGCGL1, BCAP31, B2M, ENPL, COPII, BCAP31, ERAP1) (Supplementary Figure 1). Genes encoding MHC molecules were found to be differentially expressed in SALS patients, supporting previous findings that reported a dual activity of neuronal MHC in ALS-affected tissues [10]. In fact, although MHC seems to play an essential role in preserving the maximal efficiency of motor axon connectivity with target muscles, a marked activation of this molecular complex was associated with enhanced infiltration of immune cells in the CNS of ALS animal models at the onset and during disease progression [11]. Moreover, activated microglia and astrocytes, present in ALS and other neurodegenerative diseases, showed increased expression of MHC-class II molecules, promoting the release of nitric oxide and other soluble factors that enhance inflammatory response [12]. The pharmacological inhibition of MHC I expression by immunomodulatory agents, such as glatiramer acetate, has shown neuroprotective effects in several neurological conditions, including ALS [13].

<u>T-cell-antigen recognition</u>. The presentation of antigen in the context of MHC molecules serves as a signal to trigger T cell activation and initiate an immunogenic cascade that leads to cytolysis of the APCs. A global dysregulation of T-cell functions has been related to an increased disease progression, decreased survival as well as production of pro-inflammatory effectors in experimental ALS [14, 15]. Abnormalities in T lymphocytes were also found in the blood of ALS patients, although there are differences among studies that may be explained by the heterogeneity of the ALS cohorts and the limited numbers of patients examined [14, 16, 17]. Accordingly, we found that a significant number of membrane protein-encoding genes involved in T cell activation and proliferation (*CAV1*, *TRIM1*, *TOLLIP*, *DPP4/CD26*, *CD4, MIC2, CD45, ICOS, ICOS-L*) were differentially deregulated in SALS patients (increased in SALS1 and decreased in SALS2) (Supplementary Figure 1). Among these, decreased expression of *CD4* is in line with previous evidence demonstrating that the genetic depletion of *CD4* or, more generally, a lack of functional T cells accelerates motor neuron degeneration and diminishes the survival of ALS transgenic mice, confirming a neuroprotective role of CD4+ T cells in ALS [15]. Reduced expression of *CAV1* was associated with alterations of lymphocyte trafficking and synaptic transmission in the CNS, contributing to increased risk of neurodegenerative and age-related disorders [18, 19]. SALS1

patients showed an increased expression of the gene encoding CD99 (*MIC2*), a leukocyte surface glycoprotein involved in several biological processes, including the regulation of T cell activation and development (Supplementary Figure 1) [20]. Pharmacological studies showed that CD99 blockade *in vivo* decreases the accumulation of CNS inflammatory infiltrates, supporting the role of this protein as a possible target for controlling neuroinflammatory events [21]. Another potential therapeutic target for CNS inflammation is represented by the CD45 tyrosine phosphatase, whose expression levels were increased in the spinal cord of ALS mice as well as in activated microglial cells of murine models of other neurodegenerative diseases, including Alzheimer's (AD) [22-24].

Natural Killer-cell-antigen recognition. Natural Killer (NK) cells are activated by a range of soluble factors, including cytokines and type I interferons, but also by direct cell-to-cell contact between NK cell receptors and target cell ligands. Contrary to T cells, NK cells recognize MHC I molecules using cell inhibitory receptors (i.e., KIRs, KLRs and NKG2A), leading to inhibition of NK cell activities [25-27]. Although further investigation is required to understand the role of NK cells and their receptors in ALS, previous studies have demonstrated significant infiltrations of NK cells in the spinal cord of ALS patients and the consequent inhibition of neuroprotective T-cell responses [14, 28]. In our study, we found differential expression of some genes encoding NK inhibitory receptors (*NKG2A*, *KIR2DS1*, *KIR2DS2* and *KLRA1*) in SALS patients, indicating the dysfunctions in NK cell-mediated functions may be implicated in the immunopathogenesis of various neurodegenerative diseases, including ALS (Supplementary Figure 1) [29, 30]. In accordance with our results, increased expression of *NKG2A* was previously reported in PD patients, suggesting that high levels of this receptor may induce a chronic antigen-driven stimulation and dysregulated cytokine production, contributing to inflammatory and neurodegenerative events [31].

B-cell-antigen recognition. B cells recognize a specific antigen and initiate immune response through the B cell antigen receptor (BCRs) complex consisting of an antigen-binding subunit (the membrane immunoglobulin) and a signaling subunit, which is composed of a disulfide-linked heterodimer of Ig-α (CD79A) and Ig-β (CD79B) proteins [32]. Compelling evidence supports an important role of B cells in the pathogenesis of various neurological conditions, including ALS, not only as precursors of antibody-producing cells, but also as important regulators of the T-cell activation process through their participation in antigen presentation and cytokine production [33, 34].

Our results showed differential expression of genes encoding a component of BCR complex (CD79A) and some B cell co-receptors (FcγRIIB, ITGB1, CD45, CRACM1) in SALS2 patients (Supplementary Figure 1). Low expression levels of CD79 complex and various B cell regulators were also detected in PD patients and seem to be related to aberrant protein glycosylation and folding in the endoplasmic reticulum [35]. Decreased expression of FcγRIIB or its non-functional variants was associated with the development of inflammatory autoimmune diseases and FcγRII-deficient mice showed an impaired development of Purkinje neurons and poor rotarod performance [36-38]. In accordance with our results, up-regulated expression of *CRACM1* seems to be associated with excessive neuronal Ca^{2+} signaling and excitability, thus contributing to the pathogenesis of several neurological diseases, such as epilepsy, AD and Huntington's [39, 40].

Immune and inflammatory signaling cascades

Accumulating evidence indicates that many neurodegenerative diseases, including ALS, are characterized by the massive activation and proliferation of microglia and astrocytes as well as the accumulation of infiltrating blood-derived immune cells (i.e., T lymphocytes and NK cells) at the sites of neurodegeneration, playing critical functions during the disease course. Moreover, signs of activation of the innate and humoral immune response were largely described in both ALS transgenic animal models and in the spinal cord and cortex of ALS patients [41]. In accordance with these studies, we observed differential expression of multiple components of intracellular signaling pathways regulating innate and adaptive immune responses in SALS patients (Supplementary

Figure 2). Interestingly, the majority of these signaling cascades were increased in SALS1 and reduced in SALS2 patients, suggesting that diverse subgroups of ALS patients may respond differentially to therapies targeting innate and adaptive immune responses (Figure 4b).

Immunoreceptor signaling. Once activated, antigen receptors induce a complex series of signaling events that are fundamental for various immune functions, including cell activation, proliferation, gene transcription, cytokine secretion and clonal deletion, determining the direction of immune responses. Among neuroinflammatory DEGs in SALS patients, we distinguish the altered expression of some components of the Syk and Src family kinases (LCK, LYN, FYN, SYK and ZAP70) and their regulators (SHP1 and SHP2), which represent the first signaling molecules to be activated downstream of immune cell-specific receptors (Supplementary Figure 2). The biological functions of these inflammatory mediators are diverse and include immune cell-receptor signaling, CNS myelination, cell division and adhesion, platelet function, synaptic activity and plasticity. In accordance with our findings, lack of Syk and Src-family kinases has been associated to defects in actin polymerization and remodeling at the immune synapse [42]. In particular, decreased expression of *FYN* induces a reduction in brain myelination and structural defects in synapses and dendritic spines [43]. Moreover, deregulated expression of *FYN* and *LCK* was associated with AD pathology, and several drugs that aimed at maintaining the physiological functions of the Syk and Src family kinases (e.g., saracatinib or AZD0530) are currently under study for the treatment of this disease [43, 44]. Also, *ZAP70* was increased in AD patients, suggesting that activated ZAP70 may induce neuronal death through calcium-induced lymphocyte apoptosis and/or aberrant immune responses [45]. In SALS2, we also observed decreased expression of *SYK* together with the altered expression of two protein tyrosine phosphatases, SHP-1 and SHP-2, supporting their involvement in protecting neurons from genotoxic or oxidative insults responsible for neuronal degeneration [46-49]. Together, protein kinase and phosphatase cascades impinge on multiple downstream signaling pathways which have broad effects on gene transcription (ERK/MAPK), apoptosis regulation (AKT), Ca^{2+} mobilization (PLC-γ; PKC) and cytoskeletal function modulation (Vav/Rac/Rho and AKT), representing critical events for immune activation and inflammatory cytokine/cytolytic enzyme production.

MAPK/ERK signaling. Mitogen-activated protein kinases (MAPKs) are serine-threonine kinases that are activated in response to different types of oxidative stress and inflammatory conditions, and mediate intracellular signaling associated with a variety of cellular activities, including cell proliferation, differentiation, survival and death [50]. Signal transduction via this cascade is usually initiated by activating multifunctional intracellular molecules, including guanine nucleotide exchange factors and small G proteins, which induce the sequential activation of numerous protein kinases, leading to the phosphorylation and activation of transcription factors, such as c-Jun, ATF/CREB, and p53.

In our study, we found increased mRNA expression of several components of the MAPK family (MKK4, MEK1/2, MEK3, ERK1/2, p38, and JNK) and differential expression of some of their upstream activators (Grb2, SOS, VAVs, K-RAS, H-RAS, Tiam1, CDC42, Rac1 and RhoA) in both SALS subgroups (Supplementary Figure 2). Consistent with our results, several lines of evidence demonstrated that compromised MAPK signaling pathway plays a critical role in the pathogenesis of diverse human diseases, including cancer and neurodegenerative disorders, such as ALS [51]. In fact, aberrant expression and persistent activation of p38, ERK and JNK1 have been implicated in ALS pathogenesis through various mechanisms, such as the formation of abnormal intracellular inclusions, alterations in axonal transport and cytoskeletal remodeling, and the induction of motor neuron cell death [52-54]. Interestingly, the p38 MAPK inhibitor SB203580 protects motor neurons and proximal axons from excitotoxin-induced degeneration, prolonging survival of ALS mice [55].

Dysregulation of MAPK pathway upstream regulators was reported in a variety of neuronal traumas and neurodegenerative diseases, including ALS. Among these, RHOA, a member of the Rho GTPase family, plays an important role in neuronal cell survival and death by transducing

extracellular signals to the cytoskeleton [56]. In accordance with our data, low expression levels of *RHOA* were also detected in spinal cord motor neurons from SOD1-related ALS patients [57]. In addition, disruptions in Rac signaling have been identified as an underlying factor in the progression of early onset forms of ALS [58]. Differential expression of mRNA and protein levels of the adapter protein Grb2 were previously found in both pre-symptomatic and early symptomatic ALS mice, suggesting its potential role as a candidate for ALS biomarkers [59].

PI3K/AKT/mTOR pathway. In the nervous system, activation of the PI3K signaling and its downstream effectors, AKT and mTOR, is specifically involved in diverse cellular behaviors, including proliferation, survival, metabolism, trafficking, immunity and energy homeostasis [60]. Our findings showed decreased expression of genes encoding components of PI3K, AKT and mTOR signaling cascades in both SALS patient subgroups (Supplementary Figure 2). In line with this, recent studies reported a lack or a reduction of PI3K/Akt/mTOR protein levels in spinal cord motor neurons of asymptomatic ALS mice, suggesting that modifications of this signaling pathway may represent a potential risk for motor neuron cell death [59, 61]. In particular, reduced protein expression and phosphorylation of mTOR and its downstream signaling components seem to adversely affect the establishment, maintenance and functionality of neural networks, thus contributing to neuronal degeneration and abnormal neural development [62]. In this regard, recent studies have suggested that activation of PI3K/AKT/mTOR pathways may represent an effective therapeutic strategy to prevent or slow the progression of motor neuron degeneration [56].

PLC-γ/PKC signaling pathway. Calcium signaling plays an important functional role in motor neurons, and excessive calcium release from intracellular stores seems to be responsible for the selective vulnerability of motor neurons in ALS [63]. PLC signaling is one of the principal mechanisms of maintaining calcium homeostasis in neurons. Activated PLC leads to the formation of two second messengers: IP3, which causes the release of calcium ions from intracellular stores, and DAG that activates PKC, promoting the phosphorylation and activation of calcium/calmodulin-dependent protein kinases and phosphatases as well as transcription factors involved in the production of pro-inflammatory cytokines and growth factors [64].

In our study, deregulated expression of genes encoding various components of the PLC-γ/PKC signaling cascade was detected in both SALS patient subgroups (Supplementary Figure 2). These findings are corroborated by previous studies reporting an abnormal activity or expression of PLC-γ and PKC in the spinal cord of ALS patients that results in higher cytoplasmic calcium concentrations, leading to the inhibition of MAPK and cytokine/chemokine signaling and activation of ER stress response observed in ALS [65, 66]. Moreover, the pharmacological inhibition or genetic removal of PLC-γ prolongs survival of ALS animal models, indicating this signaling as a potential target for ALS therapy [67].

RAGE /TLR Signaling Pathway. Toll-like receptor (TLR) and receptor for advanced glycation end products (RAGE) are among the major components of the innate immune system. They are able to induce and/or amplify inflammatory reactions and were extensively implicated in ALS pathology [68]. TLRs initiate signaling cascades through recognition of a variety of molecules released by the injured tissue, such as endogenous HSPs and HMGB1, a potent proinflammatory cytokine-like mediator that, interacting also with RAGE receptors, coordinates cellular stress responses and plays a critical role in several cellular processes, including cell migration, neuronal growth and apoptotic cell death [69]. Activation of RAGE/TLR signaling pathways results in the sequential stimulation of several downstream effectors and transcription factors, which induce production and release of various proinflammatory mediators (i.e., cytokines, chemokines, NO and cell adhesion molecules), exacerbating the inflammatory response and subsequently leading to neuronal damage and apoptosis.

In our study, both SALS subgroups showed up-regulation of genes encoding various TLRs (TLR2, TLR4, TLR6, TLR10), while decreased expression of HMGB1/RAGE systems was specifically detected in SALS2 patients (Supplementary Figure 2). In line with this, previous studies revealed consistent up-regulation of TLRs and HMGB1/RAGE reduction in glial cells and

degenerating spinal cord motor neurons of patients and animal models of ALS [70-72]. In addition, it was shown that TLRs antagonism, as well as restoration of RAGE signals, exert neuroprotective effects in ALS pathology, significantly extending survival and improving motor functions in a mouse model of ALS [73-75]. Altogether, these results demonstrate that activation of these signaling pathways may contribute to motor neuron injury in ALS and suggest that their pharmacological inhibition may represent an effective therapeutic strategy to attenuate neurodegenerative processes.

Among various transcription factors that are activated by the TLR signaling cascade, NF-κB plays a role of fundamental importance in various cellular mechanisms, including the immune response, cytokine production, cellular responses to oxidative stress and synaptic plasticity. Decreased expression of *NF-κB* was found in SALS1, while increased expression of this gene was detected in SALS2 (Supplementary Figure 2). This discrepancy may be explained by the fact that, while low levels of NF-κB have been associated with a decreased neuroprotection, high levels of this protein complex might be responsible for microglial activation occurring during neuroinflammatory responses [76]. Moreover, although preclinical studies reported contrasting results, several NF-κB pharmacological inhibitors have shown neuroprotective effects in ALS, mainly by preventing apoptotic cell death, inflammation and oxidative damage as well as improving mitochondrial function [77].

Complement system

The complement system represents a bridge between innate and adaptive immune responses, participating in the recognition, trafficking and elimination of pathogenic microorganisms [78]. This enzymatic cascade consists of more than 30 different proteins, membrane-bound receptors and a series of complement regulatory proteins, such as clusterin, DAF and CD59 [79]. Depending on the activation trigger, the complement cascade can be activated through three pathways (classical, lectin or alternative) that converge into the production of bioactive peptides that can mediate a variety of pro-inflammatory responses.

Complement activation in the CNS exerts a physiological role in recognizing and eliminating apoptotic and necrotic cells, but erroneous activation or insufficient regulation of this system seems to play a role in the pathogenesis of several autoimmune and neurological diseases, including ALS [80-83]. Numerous clinical and animal studies have in fact demonstrated the presence of high mRNA and protein levels of members of the complement pathway in the serum, cerebrospinal fluid and neurological tissues (spinal cord and motor cortex) of ALS patients and animal models, suggesting that a complement-driven immune response might contribute to motor neuron injury [84].

Our analysis is largely supportive of the involvement of the complement system in ALS, revealing differential expression of numerous complement components, regulators and receptors in SALS patients (Supplementary Figure 3). In particular, when compared to controls, SALS1 patients showed increased mRNA levels of several serine proteases, constituting integral elements of the classical complement cascade (including C1s, C3/C3a-c, iC3b, C3dg and C4/C4a-b) and deregulated expression of some of their membrane receptors (CD21, CRIg and CR3 - also known as $\alpha_M\beta_2$-integrin) (Supplementary Figure 3). The lectin-induced and alternative complement pathways, instead, were mainly deregulated in SALS2, with increased mRNA levels of MASP-1 and complement factors B, D, H and I (Supplementary Figure 3). Down-regulated expression of C3 and C5 convertases was found in SALS1 patients, confirming that genetic mutations or altered assembly and activity of C3/C5 convertases can result in dysregulation of the alternative complement pathway [85]. Moreover, both SALS subgroups showed deregulated expression of complement regulatory/inhibitory molecules, including clusterin, properdin, DAF and CD59, in agreement with the observation that altered levels of these molecules could confer to neurons an increased susceptibility to complement-based lysis as well as damage from recruited immune cells (Supplementary Figure 3) [83].

It is interesting to note that increased levels of complement activation products have been detected in ALS animal models before the appearance of motor symptoms, remaining detectable at the symptomatic stage, suggesting that complement activation may precede neurodegeneration and play an early role in ALS pathogenesis. This could pave the way for new diagnostic markers as well as more personalized and targeted therapeutic approaches. In this regard, inhibition or modulation of the complement system has been recognized as a promising strategy in drug discovery for several inflammatory-related conditions, including ALS, PD and AD, even if suppressing pathogenic complement activity without compromising its defensive and immunomodulatory functions still remains a big challenge [83, 86, 87]. Further experiments are thus needed to better clarify the roles of the complement pathway in the ALS progression as well as its implication in diagnosis and targeted treatments for this disease.

Cytokine signaling

Cytokines are a class of small proteins, comprising chemokines, interferons (IFNs), interleukins (ILs), lymphokines, and growth factors (like VEGF, TGF-β, TNFs), which act as signaling molecules to regulate acute and chronic inflammation and modulate cellular activities such as growth, survival, and differentiation. Cytokines exert their functions by interacting with their receptors and activating a complex network of intracellular signaling cascades, including Rac1, MEK/ MAPK/ERK1 and PI3K/AKT/mTOR and JAK-STAT pathways. It is possible to classify cytokines, based on the nature of the immune response, in pro-inflammatory cytokines (i.e., IL-1, IL-6, IL-17, TNFα and IFN-γ), and anti-inflammatory cytokines (i.e., IL-4, IL-5) [88].

Deregulated expression of several cytokines, their receptors and downstream effectors was observed in SALS patients, primarily in SALS2 (Supplementary Figure 4).

Although the pathogenic role of cytokines in ALS is still unknown, previous studies have associated their abnormal expression to the clinical status [89-91]. In accordance with our results, a variety of proinflammatory cytokines and growth factors, such as TGF-β, IL-1, IL-2, IL-4, IL-5 and IL-15, have previously been reported to be elevated in cerebrospinal fluid of human ALS [91]. In addition, activation of EGF signaling as well as reductions in VEGF signaling pathways seem to play a role in the pathogenesis of ALS, by triggering quiescent astrocytes into reactive astrocytes and, consequently, activating the neurodegenerative process [92, 93]. In support of this hypothesis, the pharmacological inhibition of IL-1, IL-6, IL-8R/CXCR2 and EGFR/ErbB2 signaling, as well as the administration of VEGF activators, reduce inflammation, provide neuroprotection and retard the progression of ALS pathology [94-101].

One of the main mechanisms for cytokine and chemokine signal transduction is represented by the JAK-STAT pathway. Differential expression of genes encoding various components of JAK/STAT signaling was found in SALS patients (Supplementary Figure 4), in agreement with previous studies showing that the dysregulation of this pathway occurs in inflammatory and neurodegenerative diseases, such as ALS [102]. Activation of the JAK-STAT pathway is also associated with the increased expression of a series of molecules involved in cytoskeleton remodeling, cell adhesion and migration, including Fibronectin, MMPs, FAK-1, VCAM1/ICAM1 receptors as well as tight junction proteins, whose deregulated expression was found in patients affected by SALS (Supplementary Figure 4) and other neurological diseases, including AD [103, 104]. It is interesting to note that the pharmacological blockade of the JAK-STAT pathway or its downstream effectors (e.g., FAK1 and MMPs) triggers neuroprotective effects, slowing disease progression and increasing survival in ALS animal models [105-108]. Altogether, these findings suggest that deciphering the complex actions of altered cytokine and chemokine networks may help to further elucidate the neuroinflammatory processes occurring in ALS.

LIST OF ABBREVIATIONS

Nardilysin convertase (NRD1); tripeptidyl peptidase 2 (TPP2); aminopeptidase puromycin sensitive (NPEPPS); bleomycin hydrolase (BLMH); thimet oligopeptidase (THOP1); SEC11 homolog C, signal peptidase complex subunit (SPC); proprotein convertase subtilisin/kexin type 7 (PC7); histocompatibility minor 13 (Impas-1); heat shock proteins (HSPs); heat shock protein 70 gene family (HSP70); heat shock protein 90 gene family (HSP90); STIP1 homology and U-box containing protein 1 (CHIP); transporter 1, ATP binding cassette subfamily B member (TAP1); transporter 2, ATP binding cassette subfamily B member (TAP2); tapasin (TPSN); protein disulfide isomerase family A member 3 (PDIA3); calreticulin (CALR); calnexin (CANX); UDP-glucose glycoprotein glucosyltransferase 1 (UGCGL1); B-cell receptor-associated protein 31 (BCAP31); beta-2-microglobulin (B2M), endoplasmin (ENPL), coat protein complex II (COPII); endoplasmic reticulum aminopeptidase 1 (ERAP1); caveolin 1 (CAV1); T cell receptor associated transmembrane adaptor 1 (TRIM1); toll interacting protein (TOLLIP); dipeptidyl peptidase 4 (DPP4); T-cell surface glycoprotein CD4 (CD4); T-cell surface antigen CD99 (MIC2); protein tyrosine phosphatase, receptor type C (CD45); inducible T-cell costimulator (ICOS); inducible T-cell costimulator ligand (ICOS-L); killer cell immunoglobulin like receptors (KIRs); killer cell lectin like receptors (KLRs); killer cell lectin like receptor C1 (NKG2A); killer cell immunoglobulin like receptor, two Ig domains and short cytoplasmic tail 1 (KIR2DS1); killer cell immunoglobulin like receptor, two Ig domains and short cytoplasmic tail 2 (KIR2DS2); killer cell lectin like receptor A1, pseudogene (KLRA1); B-cell antigen receptor complex-associated protein alpha chain (CD79A); low affinity immunoglobulin gamma Fc region receptor II (FcγRIIB), integrin subunit beta 1 (ITGB1); ORAI calcium release-activated calcium modulator 1 (CRACM1); leukocyte C-terminal Src kinase (LCK); Lck/Yes-related novel protein tyrosine kinase (LYN); tyrosine-protein kinase Fyn (FYN); spleen associated tyrosine kinase (SYK); zeta chain of T cell receptor associated protein kinase 70 (ZAP70); protein tyrosine phosphatase, non-receptor type 6 (SHP1); protein tyrosine phosphatase, non-receptor type 11 (SHP2); mitogen-activated protein kinase kinase kinase 4 (MKK4); MAP kinase kinase (MEK); extracellular signal-regulated kinase (ERK); p38 mitogen-activated protein kinase (p38 MAPK); c-Jun N-terminal kinases (JNK); growth factor receptor bound protein 2 (Grb2); SOS Ras/Rho guanine nucleotide exchange factor (SOS); vav guanine nucleotide exchange factors (VAVs); V-Ki-ras2 Kirsten rat sarcoma viral oncogene homolog (K-RAS); Harvey rat sarcoma viral oncogene homolog (H-RAS); T-cell lymphoma invasion and metastasis 1 (Tiam1); cell division cycle 42 (CDC42); ras-related C3 botulinum toxin substrate 1 (Rac1); ras homolog family member A (RhoA); phosphoinositide-3-kinase (PI3K); serine/threonine kinase (AKT); mechanistic target of rapamycin (mTOR); phospholipase C (PLC); inositol 1,4,5-trisphosphate (IP3); 1,2-diacylglycerol (DAG); protein kinase C (PKC); high mobility group box 1 (HMGB1); nuclear factor kappa B subunit 1 (NF-kB); activator protein 1 (AP-1); cAMP responsive element binding protein (CREB1); Jun proto-oncogene, AP-1 transcription factor subunit (c-Jun); decay accelerating factor (DAF/CD55); V-set and immunoglobulin domain containing 4 (CRIg); complement C3d receptor 2 (CD21); mannan binding lectin serine peptidase 1 (MASP-1); transforming growth factor beta (TGF-β); Janus kinase (JAK); signal transducer and activator of transcription (STAT); epidermal growth factor (EGF); epidermal growth factor receptor (EGFR); erb-b2 receptor tyrosine kinase 2 (ErbB2); C-X-C motif chemokine receptor 2 (IL-8R/CXCR2); matrix metallopeptidases (MMPs); focal adhesion kinase 1 (FAK-1); vascular cell adhesion molecule 1 (VCAM1); intercellular adhesion molecule 1 (ICAM1).

Supplementary References List

1. de Oliveira, G. P.; Alves, C. J.; Chadi, G., Early gene expression changes in spinal cord from SOD1(G93A) Amyotrophic Lateral Sclerosis animal model. *Frontiers in cellular neuroscience* **2013,** *7*, 216.
2. Bendotti, C.; Marino, M.; Cheroni, C.; Fontana, E.; Crippa, V.; Poletti, A.; De Biasi, S., Dysfunction of constitutive and inducible ubiquitin-proteasome system in amyotrophic lateral sclerosis: implication for protein aggregation and immune response. *Progress in neurobiology* **2012,** *97* (2), 101-26.
3. Saris, C. G.; Groen, E. J.; Koekkoek, J. A.; Veldink, J. H.; van den Berg, L. H., Meta-analysis of gene expression profiling in amyotrophic lateral sclerosis: a comparison between transgenic mouse models and human patients. *Amyotrophic lateral sclerosis & frontotemporal degeneration* **2013,** *14* (3), 177-89.
4. Cheroni, C.; Marino, M.; Tortarolo, M.; Veglianese, P.; De Biasi, S.; Fontana, E.; Zuccarello, L. V.; Maynard, C. J.; Dantuma, N. P.; Bendotti, C., Functional alterations of the ubiquitin-proteasome system in motor neurons of a mouse model of familial amyotrophic lateral sclerosis. *Human molecular genetics* **2009,** *18* (1), 82-96.
5. Ren, G.; Ma, Z.; Hui, M.; Kudo, L. C.; Hui, K. S.; Karsten, S. L., Cu, Zn-superoxide dismutase 1 (SOD1) is a novel target of Puromycin-sensitive aminopeptidase (PSA/NPEPPS): PSA/NPEPPS is a possible modifier of amyotrophic lateral sclerosis. *Molecular neurodegeneration* **2011,** *6*, 29.
6. Ohno, M.; Hiraoka, Y.; Matsuoka, T.; Tomimoto, H.; Takao, K.; Miyakawa, T.; Oshima, N.; Kiyonari, H.; Kimura, T.; Kita, T.; Nishi, E., Nardilysin regulates axonal maturation and myelination in the central and peripheral nervous system. *Nature neuroscience* **2009,** *12* (12), 1506-13.
7. Moliaka, Y. K.; Grigorenko, A.; Madera, D.; Rogaev, E. I., Impas 1 possesses endoproteolytic activity against multipass membrane protein substrate cleaving the presenilin 1 holoprotein. *FEBS letters* **2004,** *557* (1-3), 185-92.
8. Kalmar, B.; Lu, C. H.; Greensmith, L., The role of heat shock proteins in Amyotrophic Lateral Sclerosis: The therapeutic potential of Arimoclomol. *Pharmacology & therapeutics* **2014,** *141* (1), 40-54.
9. Choi, J. S.; Cho, S.; Park, S. G.; Park, B. C.; Lee, D. H., Co-chaperone CHIP associates with mutant Cu/Zn-superoxide dismutase proteins linked to familial amyotrophic lateral sclerosis and promotes their degradation by proteasomes. *Biochemical and biophysical research communications* **2004,** *321* (3), 574-83.
10. Lampson, L. A.; Kushner, P. D.; Sobel, R. A., Major histocompatibility complex antigen expression in the affected tissues in amyotrophic lateral sclerosis. *Ann Neurol* **1990,** *28* (3), 365-72.
11. Nardo, G.; Trolese, M. C.; Bendotti, C., Major Histocompatibility Complex I Expression by Motor Neurons and Its Implication in Amyotrophic Lateral Sclerosis. *Frontiers in neurology* **2016,** *7*, 89.
12. Amor, S.; Puentes, F.; Baker, D.; van der Valk, P., Inflammation in neurodegenerative diseases. *Immunology* **2010,** *129* (2), 154-69.
13. Scorisa, J. M.; Freria, C. M.; Victorio, S. C.; Barbizan, R.; Zanon, R. G.; Oliveira, A. L., Glatiramer acetate treatment increases stability of spinal synapses and down regulates MHC I during the course of EAE. *International journal of biological sciences* **2011,** *7* (8), 1188-202.
14. Rentzos, M.; Evangelopoulos, E.; Sereti, E.; Zouvelou, V.; Marmara, S.; Alexakis, T.; Evdokimidis, I., Alterations of T cell subsets in ALS: a systemic immune activation? *Acta neurologica Scandinavica* **2012,** *125* (4), 260-4.
15. Beers, D. R.; Henkel, J. S.; Zhao, W.; Wang, J.; Huang, A.; Wen, S.; Liao, B.; Appel, S. H., Endogenous regulatory T lymphocytes ameliorate amyotrophic lateral sclerosis in mice and

correlate with disease progression in patients with amyotrophic lateral sclerosis. *Brain : a journal of neurology* **2011,** *134* (Pt 5), 1293-314.
16. Henkel, J. S.; Beers, D. R.; Wen, S.; Rivera, A. L.; Toennis, K. M.; Appel, J. E.; Zhao, W.; Moore, D. H.; Powell, S. Z.; Appel, S. H., Regulatory T-lymphocytes mediate amyotrophic lateral sclerosis progression and survival. *EMBO molecular medicine* **2013,** *5* (1), 64-79.
17. Mantovani, S.; Garbelli, S.; Pasini, A.; Alimonti, D.; Perotti, C.; Melazzini, M.; Bendotti, C.; Mora, G., Immune system alterations in sporadic amyotrophic lateral sclerosis patients suggest an ongoing neuroinflammatory process. *Journal of neuroimmunology* **2009,** *210* (1-2), 73-9.
18. Liu, Y.; Liang, Z.; Liu, J.; Zou, W.; Li, X.; Wang, Y.; An, L., Downregulation of caveolin-1 contributes to the synaptic plasticity deficit in the hippocampus of aged rats. *Neural regeneration research* **2013,** *8* (29), 2725-33.
19. Head, B. P.; Peart, J. N.; Panneerselvam, M.; Yokoyama, T.; Pearn, M. L.; Niesman, I. R.; Bonds, J. A.; Schilling, J. M.; Miyanohara, A.; Headrick, J.; Ali, S. S.; Roth, D. M.; Patel, P. M.; Patel, H. H., Loss of caveolin-1 accelerates neurodegeneration and aging. *PloS one* **2010,** *5* (12), e15697.
20. Sohn, H. W.; Choi, E. Y.; Kim, S. H.; Lee, I. S.; Chung, D. H.; Sung, U. A.; Hwang, D. H.; Cho, S. S.; Jun, B. H.; Jang, J. J.; Chi, J. G.; Park, S. H., Engagement of CD99 induces apoptosis through a calcineurin-independent pathway in Ewing's sarcoma cells. *The American journal of pathology* **1998,** *153* (6), 1937-45.
21. Winger, R. C.; Harp, C. T.; Chiang, M. Y.; Sullivan, D. P.; Watson, R. L.; Weber, E. W.; Podojil, J. R.; Miller, S. D.; Muller, W. A., Cutting Edge: CD99 Is a Novel Therapeutic Target for Control of T Cell-Mediated Central Nervous System Autoimmune Disease. *Journal of immunology* **2016,** *196* (4), 1443-8.
22. Rathke-Hartlieb, S.; Schmidt, V. C.; Jockusch, H.; Schmitt-John, T.; Bartsch, J. W., Spatiotemporal progression of neurodegeneration and glia activation in the wobbler neuropathy of the mouse. *Neuroreport* **1999,** *10* (16), 3411-6.
23. Townsend, K. P.; Vendrame, M.; Ehrhart, J.; Faza, B.; Zeng, J.; Town, T.; Tan, J., CD45 isoform RB as a molecular target to oppose lipopolysaccharide-induced microglial activation in mice. *Neuroscience letters* **2004,** *362* (1), 26-30.
24. Tan, J.; Town, T.; Mullan, M., CD45 inhibits CD40L-induced microglial activation via negative regulation of the Src/p44/42 MAPK pathway. *The Journal of biological chemistry* **2000,** *275* (47), 37224-31.
25. Borrego, F.; Kabat, J.; Kim, D. K.; Lieto, L.; Maasho, K.; Pena, J.; Solana, R.; Coligan, J. E., Structure and function of major histocompatibility complex (MHC) class I specific receptors expressed on human natural killer (NK) cells. *Molecular immunology* **2002,** *38* (9), 637-60.
26. Maghazachi, A. A., Role of chemokines in the biology of natural killer cells. *Current topics in microbiology and immunology* **2010,** *341*, 37-58.
27. Malhotra, A.; Shanker, A., NK cells: immune cross-talk and therapeutic implications. *Immunotherapy* **2011,** *3* (10), 1143-66.
28. Philips, T.; Rothstein, J. D., Glial cells in amyotrophic lateral sclerosis. *Experimental neurology* **2014,** *262 Pt B*, 111-20.
29. Pool, M.; Rambaldi, I.; Darlington, P. J.; Wright, M. C.; Fournier, A. E.; Bar-Or, A., Neurite outgrowth is differentially impacted by distinct immune cell subsets. *Molecular and cellular neurosciences* **2012,** *49* (1), 68-76.
30. Jadidi-Niaragh, F.; Shegarfi, H.; Naddafi, F.; Mirshafiey, A., The role of natural killer cells in Alzheimer's disease. *Scandinavian journal of immunology* **2012,** *76* (5), 451-6.
31. Poli, A.; Kmiecik, J.; Domingues, O.; Hentges, F.; Blery, M.; Chekenya, M.; Boucraut, J.; Zimmer, J., NK cells in central nervous system disorders. *Journal of immunology* **2013,** *190* (11), 5355-62.
32. Pieper, K.; Grimbacher, B.; Eibel, H., B-cell biology and development. *The Journal of allergy and clinical immunology* **2013,** *131* (4), 959-71.

33. McCombe, P. A.; Henderson, R. D., The Role of immune and inflammatory mechanisms in ALS. *Current molecular medicine* **2011,** *11* (3), 246-54.
34. Naor, S.; Keren, Z.; Bronshtein, T.; Goren, E.; Machluf, M.; Melamed, D., Development of ALS-like disease in SOD-1 mice deficient of B lymphocytes. *Journal of neurology* **2009,** *256* (8), 1228-35.
35. Kedmi, M.; Bar-Shira, A.; Gurevich, T.; Giladi, N.; Orr-Urtreger, A., Decreased expression of B cell related genes in leukocytes of women with Parkinson's disease. *Molecular neurodegeneration* **2011,** *6*, 66.
36. Comabella, M.; Montalban, X.; Kakalacheva, K.; Osman, D.; Nimmerjahn, F.; Tintore, M.; Lunemann, J. D., B cell expression of the inhibitory Fc gamma receptor is unchanged in early MS. *Journal of neuroimmunology* **2010,** *223* (1-2), 135-7.
37. Li, X.; Kimberly, R. P., Targeting the Fc receptor in autoimmune disease. *Expert opinion on therapeutic targets* **2014,** *18* (3), 335-50.
38. Nakamura, K.; Hirai, H.; Torashima, T.; Miyazaki, T.; Tsurui, H.; Xiu, Y.; Ohtsuji, M.; Lin, Q. S.; Tsukamoto, K.; Nishimura, H.; Ono, M.; Watanabe, M.; Hirose, S., CD3 and immunoglobulin G Fc receptor regulate cerebellar functions. *Molecular and cellular biology* **2007,** *27* (14), 5128-34.
39. Moccia, F.; Zuccolo, E.; Soda, T.; Tanzi, F.; Guerra, G.; Mapelli, L.; Lodola, F.; D'Angelo, E., Stim and Orai proteins in neuronal Ca(2+) signaling and excitability. *Frontiers in cellular neuroscience* **2015,** *9*, 153.
40. Vigont, V.; Kolobkova, Y.; Skopin, A.; Zimina, O.; Zenin, V.; Glushankova, L.; Kaznacheyeva, E., Both Orai1 and TRPC1 are Involved in Excessive Store-Operated Calcium Entry in Striatal Neurons Expressing Mutant Huntingtin Exon 1. *Frontiers in physiology* **2015,** *6*, 337.
41. Lewis, C. A.; Manning, J.; Rossi, F.; Krieger, C., The Neuroinflammatory Response in ALS: The Roles of Microglia and T Cells. *Neurology research international* **2012,** *2012*, 803701.
42. Lowell, C. A., Src-family and Syk kinases in activating and inhibitory pathways in innate immune cells: signaling cross talk. *Cold Spring Harbor perspectives in biology* **2011,** *3* (3).
43. Nygaard, H. B.; van Dyck, C. H.; Strittmatter, S. M., Fyn kinase inhibition as a novel therapy for Alzheimer's disease. *Alzheimer's research & therapy* **2014,** *6* (1), 8.
44. Hata, R.; Masumura, M.; Akatsu, H.; Li, F.; Fujita, H.; Nagai, Y.; Yamamoto, T.; Okada, H.; Kosaka, K.; Sakanaka, M.; Sawada, T., Up-regulation of calcineurin Abeta mRNA in the Alzheimer's disease brain: assessment by cDNA microarray. *Biochemical and biophysical research communications* **2001,** *284* (2), 310-6.
45. Ho Kim, J.; Franck, J.; Kang, T.; Heinsen, H.; Ravid, R.; Ferrer, I.; Hee Cheon, M.; Lee, J. Y.; Shin Yoo, J.; Steinbusch, H. W.; Salzet, M.; Fournier, I.; Mok Park, Y., Proteome-wide characterization of signalling interactions in the hippocampal CA4/DG subfield of patients with Alzheimer's disease. *Scientific reports* **2015,** *5*, 11138.
46. Krisenko, M. O.; Higgins, R. L.; Ghosh, S.; Zhou, Q.; Trybula, J. S.; Wang, W. H.; Geahlen, R. L., Syk Is Recruited to Stress Granules and Promotes Their Clearance through Autophagy. *The Journal of biological chemistry* **2015,** *290* (46), 27803-15.
47. Park, H.; Ahn, K. J.; Lee Kang, J.; Choi, Y. H., Protein-protein interaction between caveolin-1 and SHP-2 is dependent on the N-SH2 domain of SHP-2. *BMB reports* **2015,** *48* (3), 184-9.
48. Takai, S.; Yamada, M.; Araki, T.; Koshimizu, H.; Nawa, H.; Hatanaka, H., Shp-2 positively regulates brain-derived neurotrophic factor-promoted survival of cultured ventral mesencephalic dopaminergic neurons through a brain immunoglobulin-like molecule with tyrosine-based activation motifs/Shp substrate-1. *Journal of neurochemistry* **2002,** *82* (2), 353-64.
49. Chong, Z. Z.; Maiese, K., The Src homology 2 domain tyrosine phosphatases SHP-1 and SHP-2: diversified control of cell growth, inflammation, and injury. *Histology and histopathology* **2007,** *22* (11), 1251-67.

50. Kim, E. K.; Choi, E. J., Pathological roles of MAPK signaling pathways in human diseases. *Biochimica et biophysica acta* **2010,** *1802* (4), 396-405.
51. Kim, E. K.; Choi, E. J., Compromised MAPK signaling in human diseases: an update. *Archives of toxicology* **2015,** *89* (6), 867-82.
52. Gurney, M. E.; Pu, H.; Chiu, A. Y.; Dal Canto, M. C.; Polchow, C. Y.; Alexander, D. D.; Caliendo, J.; Hentati, A.; Kwon, Y. W.; Deng, H. X.; et al., Motor neuron degeneration in mice that express a human Cu,Zn superoxide dismutase mutation. *Science* **1994,** *264* (5166), 1772-5.
53. Holasek, S. S.; Wengenack, T. M.; Kandimalla, K. K.; Montano, C.; Gregor, D. M.; Curran, G. L.; Poduslo, J. F., Activation of the stress-activated MAP kinase, p38, but not JNK in cortical motor neurons during early presymptomatic stages of amyotrophic lateral sclerosis in transgenic mice. *Brain research* **2005,** *1045* (1-2), 185-98.
54. Bendotti, C.; Atzori, C.; Piva, R.; Tortarolo, M.; Strong, M. J.; DeBiasi, S.; Migheli, A., Activated p38MAPK is a novel component of the intracellular inclusions found in human amyotrophic lateral sclerosis and mutant SOD1 transgenic mice. *Journal of neuropathology and experimental neurology* **2004,** *63* (2), 113-9.
55. Dewil, M.; dela Cruz, V. F.; Van Den Bosch, L.; Robberecht, W., Inhibition of p38 mitogen activated protein kinase activation and mutant SOD1(G93A)-induced motor neuron death. *Neurobiology of disease* **2007,** *26* (2), 332-41.
56. Kirby, J.; Ning, K.; Ferraiuolo, L.; Heath, P. R.; Ismail, A.; Kuo, S. W.; Valori, C. F.; Cox, L.; Sharrack, B.; Wharton, S. B.; Ince, P. G.; Shaw, P. J.; Azzouz, M., Phosphatase and tensin homologue/protein kinase B pathway linked to motor neuron survival in human superoxide dismutase 1-related amyotrophic lateral sclerosis. *Brain : a journal of neurology* **2011,** *134* (Pt 2), 506-17.
57. Linseman, D. A.; Loucks, F. A., Diverse roles of Rho family GTPases in neuronal development, survival, and death. *Frontiers in bioscience : a journal and virtual library* **2008,** *13*, 657-76.
58. Kanekura, K.; Hashimoto, Y.; Kita, Y.; Sasabe, J.; Aiso, S.; Nishimoto, I.; Matsuoka, M., A Rac1/phosphatidylinositol 3-kinase/Akt3 anti-apoptotic pathway, triggered by AlsinLF, the product of the ALS2 gene, antagonizes Cu/Zn-superoxide dismutase (SOD1) mutant-induced motoneuronal cell death. *The Journal of biological chemistry* **2005,** *280* (6), 4532-43.
59. de Oliveira, G. P.; Maximino, J. R.; Maschietto, M.; Zanoteli, E.; Puga, R. D.; Lima, L.; Carraro, D. M.; Chadi, G., Early gene expression changes in skeletal muscle from SOD1(G93A) amyotrophic lateral sclerosis animal model. *Cellular and molecular neurobiology* **2014,** *34* (3), 451-62.
60. Brunet, A.; Datta, S. R.; Greenberg, M. E., Transcription-dependent and -independent control of neuronal survival by the PI3K-Akt signaling pathway. *Current opinion in neurobiology* **2001,** *11* (3), 297-305.
61. Yin, X.; Ren, M.; Jiang, H.; Cui, S.; Wang, S.; Jiang, H.; Qi, Y.; Wang, J.; Wang, X.; Dong, G.; Leeds, P.; Chuang, D. M.; Feng, H., Downregulated AEG-1 together with inhibited PI3K/Akt pathway is associated with reduced viability of motor neurons in an ALS model. *Molecular and cellular neurosciences* **2015,** *68*, 303-13.
62. Nicolini, C.; Ahn, Y.; Michalski, B.; Rho, J. M.; Fahnestock, M., Decreased mTOR signaling pathway in human idiopathic autism and in rats exposed to valproic acid. *Acta neuropathologica communications* **2015,** *3*, 3.
63. Grosskreutz, J.; Van Den Bosch, L.; Keller, B. U., Calcium dysregulation in amyotrophic lateral sclerosis. *Cell calcium* **2010,** *47* (2), 165-74.
64. Kopec, A.; Panaszek, B.; Fal, A. M., Intracellular signaling pathways in IgE-dependent mast cell activation. *Archivum immunologiae et therapiae experimentalis* **2006,** *54* (6), 393-401.
65. Hu, J. H.; Zhang, H.; Wagey, R.; Krieger, C.; Pelech, S. L., Protein kinase and protein phosphatase expression in amyotrophic lateral sclerosis spinal cord. *Journal of neurochemistry* **2003,** *85* (2), 432-442.

66. Tadic, V.; Prell, T.; Lautenschlaeger, J.; Grosskreutz, J., The ER mitochondria calcium cycle and ER stress response as therapeutic targets in amyotrophic lateral sclerosis. *Frontiers in cellular neuroscience* **2014,** *8*, 147.
67. Staats, K. A.; Van Helleputte, L.; Jones, A. R.; Bento-Abreu, A.; Van Hoecke, A.; Shatunov, A.; Simpson, C. L.; Lemmens, R.; Jaspers, T.; Fukami, K.; Nakamura, Y.; Brown, R. H., Jr.; Van Damme, P.; Liston, A.; Robberecht, W.; Al-Chalabi, A.; Van Den Bosch, L., Genetic ablation of phospholipase C delta 1 increases survival in SOD1(G93A) mice. *Neurobiology of disease* **2013,** *60*, 11-7.
68. Casula, M.; Iyer, A. M.; Spliet, W. G.; Anink, J. J.; Steentjes, K.; Sta, M.; Troost, D.; Aronica, E., Toll-like receptor signaling in amyotrophic lateral sclerosis spinal cord tissue. *Neuroscience* **2011,** *179*, 233-43.
69. Ray, R.; Juranek, J. K.; Rai, V., RAGE axis in neuroinflammation, neurodegeneration and its emerging role in the pathogenesis of amyotrophic lateral sclerosis. *Neuroscience and biobehavioral reviews* **2016,** *62*, 48-55.
70. Letiembre, M.; Liu, Y.; Walter, S.; Hao, W.; Pfander, T.; Wrede, A.; Schulz-Schaeffer, W.; Fassbender, K., Screening of innate immune receptors in neurodegenerative diseases: a similar pattern. *Neurobiol Aging* **2009,** *30* (5), 759-68.
71. Liu, Y.; Hao, W.; Dawson, A.; Liu, S.; Fassbender, K., Expression of amyotrophic lateral sclerosis-linked SOD1 mutant increases the neurotoxic potential of microglia via TLR2. *The Journal of biological chemistry* **2009,** *284* (6), 3691-9.
72. Lo Coco, D.; Veglianese, P.; Allievi, E.; Bendotti, C., Distribution and cellular localization of high mobility group box protein 1 (HMGB1) in the spinal cord of a transgenic mouse model of ALS. *Neuroscience letters* **2007,** *412* (1), 73-7.
73. De Paola, M.; Mariani, A.; Bigini, P.; Peviani, M.; Ferrara, G.; Molteni, M.; Gemma, S.; Veglianese, P.; Castellaneta, V.; Boldrin, V.; Rossetti, C.; Chiabrando, C.; Forloni, G.; Mennini, T.; Fanelli, R., Neuroprotective effects of toll-like receptor 4 antagonism in spinal cord cultures and in a mouse model of motor neuron degeneration. *Molecular medicine* **2012,** *18*, 971-81.
74. Zhao, W.; Beers, D. R.; Henkel, J. S.; Zhang, W.; Urushitani, M.; Julien, J. P.; Appel, S. H., Extracellular mutant SOD1 induces microglial-mediated motoneuron injury. *Glia* **2010,** *58* (2), 231-43.
75. Zhao, W.; Beers, D. R.; Appel, S. H., Immune-mediated mechanisms in the pathoprogression of amyotrophic lateral sclerosis. *Journal of neuroimmune pharmacology : the official journal of the Society on NeuroImmune Pharmacology* **2013,** *8* (4), 888-99.
76. Camandola, S.; Mattson, M. P., NF-kappa B as a therapeutic target in neurodegenerative diseases. *Expert opinion on therapeutic targets* **2007,** *11* (2), 123-32.
77. Feng, X. H.; Yuan, W.; Peng, Y.; Liu, M. S.; Cui, L. Y., Therapeutic effects of dl-3-n-butylphthalide in a transgenic mouse model of amyotrophic lateral sclerosis. *Chinese medical journal* **2012,** *125* (10), 1760-6.
78. Morley, B. J.; Walport, M. J., 2 - The Complement System. In *The Complement FactsBook*, Academic Press: London, 2000; pp 7-22.
79. Guo, R. F.; Ward, P. A., Role of C5a in inflammatory responses. *Annual review of immunology* **2005,** *23*, 821-52.
80. Bonifati, D. M.; Kishore, U., Role of complement in neurodegeneration and neuroinflammation. *Molecular immunology* **2007,** *44* (5), 999-1010.
81. Morgan, B. P., The role of complement in neurological and neuropsychiatric diseases. *Expert review of clinical immunology* **2015,** *11* (10), 1109-19.
82. Lee, J. D.; Kamaruzaman, N. A.; Fung, J. N.; Taylor, S. M.; Turner, B. J.; Atkin, J. D.; Woodruff, T. M.; Noakes, P. G., Dysregulation of the complement cascade in the hSOD1G93A transgenic mouse model of amyotrophic lateral sclerosis. *Journal of neuroinflammation* **2013,** *10*, 119.

83. Orsini, F.; De Blasio, D.; Zangari, R.; Zanier, E. R.; De Simoni, M. G., Versatility of the complement system in neuroinflammation, neurodegeneration and brain homeostasis. *Frontiers in cellular neuroscience* **2014,** *8*, 380.
84. Bahia El Idrissi, N.; Bosch, S.; Ramaglia, V.; Aronica, E.; Baas, F.; Troost, D., Complement activation at the motor end-plates in amyotrophic lateral sclerosis. *Journal of neuroinflammation* **2016,** *13* (1), 72.
85. Fervenza, F. C.; Sethi, S., Circulating complement levels and C3 glomerulopathy. *Clinical journal of the American Society of Nephrology : CJASN* **2014,** *9* (11), 1829-31.
86. Kolev, M. V.; Ruseva, M. M.; Harris, C. L.; Morgan, B. P.; Donev, R. M., Implication of complement system and its regulators in Alzheimer's disease. *Current neuropharmacology* **2009,** *7* (1), 1-8.
87. Melis, J. P.; Strumane, K.; Ruuls, S. R.; Beurskens, F. J.; Schuurman, J.; Parren, P. W., Complement in therapy and disease: Regulating the complement system with antibody-based therapeutics. *Molecular immunology* **2015,** *67* (2 Pt A), 117-30.
88. Turner, M. D.; Nedjai, B.; Hurst, T.; Pennington, D. J., Cytokines and chemokines: At the crossroads of cell signalling and inflammatory disease. *Biochimica et biophysica acta* **2014,** *1843* (11), 2563-2582.
89. Rentzos, M.; Rombos, A.; Nikolaou, C.; Zoga, M.; Zouvelou, V.; Dimitrakopoulos, A.; Alexakis, T.; Tsoutsou, A.; Samakovli, A.; Michalopoulou, M.; Evdokimidis, J., Interleukin-17 and interleukin-23 are elevated in serum and cerebrospinal fluid of patients with ALS: a reflection of Th17 cells activation? *Acta neurologica Scandinavica* **2010,** *122* (6), 425-9.
90. Malaspina, A.; Puentes, F.; Amor, S., Disease origin and progression in amyotrophic lateral sclerosis: an immunology perspective. *International immunology* **2015,** *27* (3), 117-29.
91. Tateishi, T.; Yamasaki, R.; Tanaka, M.; Matsushita, T.; Kikuchi, H.; Isobe, N.; Ohyagi, Y.; Kira, J., CSF chemokine alterations related to the clinical course of amyotrophic lateral sclerosis. *Journal of neuroimmunology* **2010,** *222* (1-2), 76-81.
92. Liu, B.; Chen, H.; Johns, T. G.; Neufeld, A. H., Epidermal growth factor receptor activation: an upstream signal for transition of quiescent astrocytes into reactive astrocytes after neural injury. *J Neurosci* **2006,** *26* (28), 7532-40.
93. Brockington, A.; Wharton, S. B.; Fernando, M.; Gelsthorpe, C. H.; Baxter, L.; Ince, P. G.; Lewis, C. E.; Shaw, P. J., Expression of vascular endothelial growth factor and its receptors in the central nervous system in amyotrophic lateral sclerosis. *J Neuropathol Exp Neurol* **2006,** *65* (1), 26-36.
94. Maier, A.; Deigendesch, N.; Muller, K.; Weishaupt, J. H.; Krannich, A.; Rohle, R.; Meissner, F.; Molawi, K.; Munch, C.; Holm, T.; Meyer, R.; Meyer, T.; Zychlinsky, A., Interleukin-1 Antagonist Anakinra in Amyotrophic Lateral Sclerosis--A Pilot Study. *PloS one* **2015,** *10* (10), e0139684.
95. Mizwicki, M. T.; Fiala, M.; Magpantay, L.; Aziz, N.; Sayre, J.; Liu, G.; Siani, A.; Chan, D.; Martinez-Maza, O.; Chattopadhyay, M.; La Cava, A., Tocilizumab attenuates inflammation in ALS patients through inhibition of IL6 receptor signaling. *American journal of neurodegenerative disease* **2012,** *1* (3), 305-15.
96. Boston-Howes, W.; Williams, E. O.; Bogush, A.; Scolere, M.; Pasinelli, P.; Trotti, D., Nordihydroguaiaretic acid increases glutamate uptake in vitro and in vivo: therapeutic implications for amyotrophic lateral sclerosis. *Exp Neurol* **2008,** *213* (1), 229-37.
97. Trieu, V. N.; Uckun, F. M., Genistein is neuroprotective in murine models of familial amyotrophic lateral sclerosis and stroke. *Biochem Biophys Res Commun* **1999,** *258* (3), 685-8.
98. Tovar-y-Romo, L. B.; Tapia, R., Delayed administration of VEGF rescues spinal motor neurons from death with a short effective time frame in excitotoxic experimental models in vivo. *ASN Neuro* **2012,** *4* (2).
99. Zheng, C.; Nennesmo, I.; Fadeel, B.; Henter, J. I., Vascular endothelial growth factor prolongs survival in a transgenic mouse model of ALS. *Ann Neurol* **2004,** *56* (4), 564-7.

100. Nefussy, B.; Drory, V. E., Moving toward a predictive and personalized clinical approach in amyotrophic lateral sclerosis: novel developments and future directions in diagnosis, genetics, pathogenesis and therapies. *The EPMA journal* **2010,** *1* (2), 329-41.
101. Gordon, P. H.; Cheung, Y. K.; Levin, B.; Andrews, H.; Doorish, C.; Macarthur, R. B.; Montes, J.; Bednarz, K.; Florence, J.; Rowin, J.; Boylan, K.; Mozaffar, T.; Tandan, R.; Mitsumoto, H.; Kelvin, E. A.; Chapin, J.; Bedlack, R.; Rivner, M.; McCluskey, L. F.; Pestronk, A.; Graves, M.; Sorenson, E. J.; Barohn, R. J.; Belsh, J. M.; Lou, J. S.; Levine, T.; Saperstein, D.; Miller, R. G.; Scelsa, S. N.; Combination Drug Selection Trial Study, G., A novel, efficient, randomized selection trial comparing combinations of drug therapy for ALS. *Amyotroph Lateral Scler* **2008,** *9* (4), 212-22.
102. Nicolas, C. S.; Amici, M.; Bortolotto, Z. A.; Doherty, A.; Csaba, Z.; Fafouri, A.; Dournaud, P.; Gressens, P.; Collingridge, G. L.; Peineau, S., The role of JAK-STAT signaling within the CNS. *Jak-Stat* **2013,** *2* (1), e22925.
103. Grace, E. A.; Busciglio, J., Aberrant activation of focal adhesion proteins mediates fibrillar amyloid beta-induced neuronal dystrophy. *J Neurosci* **2003,** *23* (2), 493-502.
104. Niebroj-Dobosz, I.; Janik, P.; Sokolowska, B.; Kwiecinski, H., Matrix metalloproteinases and their tissue inhibitors in serum and cerebrospinal fluid of patients with amyotrophic lateral sclerosis. *European journal of neurology* **2010,** *17* (2), 226-31.
105. Trieu, V. N.; Liu, R.; Liu, X. P.; Uckun, F. M., A specific inhibitor of janus kinase-3 increases survival in a transgenic mouse model of amyotrophic lateral sclerosis. *Biochem Biophys Res Commun* **2000,** *267* (1), 22-5.
106. Qin, H.; Buckley, J. A.; Li, X.; Liu, Y.; Fox, T. H., 3rd; Meares, G. P.; Yu, H.; Yan, Z.; Harms, A. S.; Li, Y.; Standaert, D. G.; Benveniste, E. N., Inhibition of the JAK/STAT Pathway Protects Against alpha-Synuclein-Induced Neuroinflammation and Dopaminergic Neurodegeneration. *The Journal of neuroscience : the official journal of the Society for Neuroscience* **2016,** *36* (18), 5144-59.
107. Kiaei, M.; Petri, S.; Kipiani, K.; Gardian, G.; Choi, D. K.; Chen, J.; Calingasan, N. Y.; Schafer, P.; Muller, G. W.; Stewart, C.; Hensley, K.; Beal, M. F., Thalidomide and lenalidomide extend survival in a transgenic mouse model of amyotrophic lateral sclerosis. *J Neurosci* **2006,** *26* (9), 2467-73.
108. Kiaei, M.; Kipiani, K.; Petri, S.; Choi, D. K.; Chen, J.; Calingasan, N. Y.; Beal, M. F., Integrative role of cPLA with COX-2 and the effect of non-steriodal anti-inflammatory drugs in a transgenic mouse model of amyotrophic lateral sclerosis. *Journal of neurochemistry* **2005,** *93* (2), 403-11.

Supplementary Figure and Table Legends

Supplementary Figure 1. Alterations in the antigen processing and presentation pathway associated with SALS patients.

This figure illustrates genes differentially expressed in SALS patients that are involved in the antigen processing and presentation process. Each encoded protein is labeled with two thermometers (1) or (2) that indicate expression levels in SALS cluster 1 and 2, respectively. Up-ward thermometers have red color and indicate up-regulated signals in SALS patients, down-ward (blue) ones indicate down-regulated signals. Colored hexagons on the vectors between objects describe the type of interaction where B = binding, C = cleavage, CM = covalent modification, Cn = competition, CS = complex subunit, GR = group relation, IE = influence on expression, P = phosphorylation, T = transformation, TR = transcriptional regulation, Tn = transport and CR indicates that an object belongs to a group of related proteins. Lines indicate activation (green), inhibition (red) or unspecified (grey) interactions between the molecules. The object shapes correspond to molecule type and are described in the Supplementary Figure 5.

Supplementary Figure 2. Alterations in immune and inflammatory signaling observed in SALS patients.

Genes involved in immune and inflammatory signaling that were differentially expressed in SALS patients versus controls were mapped on pathway. Thermometers labeled with (1) or (2) indicate expression levels in SALS cluster 1 and 2, respectively. Up-ward thermometers have red color and indicate up-regulated signals in SALS patients, down-ward (blue) ones indicate down-regulated signals. Colored hexagons on the vectors between objects describe the type of interaction where B = binding, C = cleavage, CM = covalent modification, Cn = competition, CS = complex subunit, GR = group relation, IE = influence on expression, P = phosphorylation, T = transformation, TR = transcriptional regulation, Tn = transport and CR indicates that an object belongs to a group of related proteins. Lines indicate activation (green), inhibition (red) or unspecified (grey) interactions between the molecules. The object shapes correspond to molecule type and are described in the Supplementary Figure 5.

Supplementary Figure 3. Alterations in complement system signaling pathways (classical, lectin and alternative) observed in SALS patients.

Thermometers labeled with (1) or (2) indicate expression levels in SALS cluster 1 and 2, respectively. Up-ward thermometers have red color and indicate up-regulated signals in SALS patients, down-ward (blue) ones indicate down-regulated signals. Colored hexagons on the vectors between objects describe the type of interaction where B = binding, C = cleavage, CM = covalent modification, Cn = competition, CS = complex subunit, GR = group relation, IE = influence on

expression, P = phosphorylation, T = transformation, TR = transcriptional regulation, Tn = transport and CR indicates that an object belongs to a group of related proteins. Lines indicate activation (green), inhibition (red) or unspecified (grey) interactions between the molecules. The object shapes correspond to molecule type and are described in the Supplementary Figure 5.

Supplementary Figure 4. Alterations in cytokine signaling observed in SALS patients. Thermometers labeled with (1) or (2) indicate expression levels in SALS cluster 1 and 2, respectively. Up-ward thermometers have red color and indicate up-regulated signals in SALS patients, down-ward (blue) ones indicate down-regulated signals. Colored hexagons on the vectors between objects describe the type of interaction where B = binding, C = cleavage, CM = covalent modification, Cn = competition, CS = complex subunit, GR = group relation, IE = influence on expression, P = phosphorylation, T = transformation, TR = transcriptional regulation, Tn = transport and CR indicates that an object belongs to a group of related proteins. Lines indicate activation (green), inhibition (red) or unspecified (grey) interactions between the molecules. The object shapes correspond to molecule type and are described in the Supplementary Figure 5.

Supplementary Figure 5. Legend describing symbols used in MetaCore pathway.

Supplementary Table 1. Pathway enrichment analysis on the entire list of statistically deregulated genes in SALS patients versus controls.

Supplementary Table 2. Pathway enrichment analysis on the entire list of statistically deregulated genes in SALS1 and SALS2 patients compared to controls.

Supplementary Table 3. Neuroinflammatory genes differentially expressed in SALS1.

Supplementary Table 4. Neuroinflammatory genes differentially expressed in SALS2.

Supplementary Table 5. Neuroinflammatory genes differentially expressed in SALS1 and SALS2.

Supplementary Table 6. List of genes included in the SALS1-related PPI network.

Supplementary Table 7. List of genes included in the SALS2-related PPI network.

Supplementary Table 8. Pathway enrichment analyses on the list of statistically deregulated neuroinflammatory genes in SALS patients.

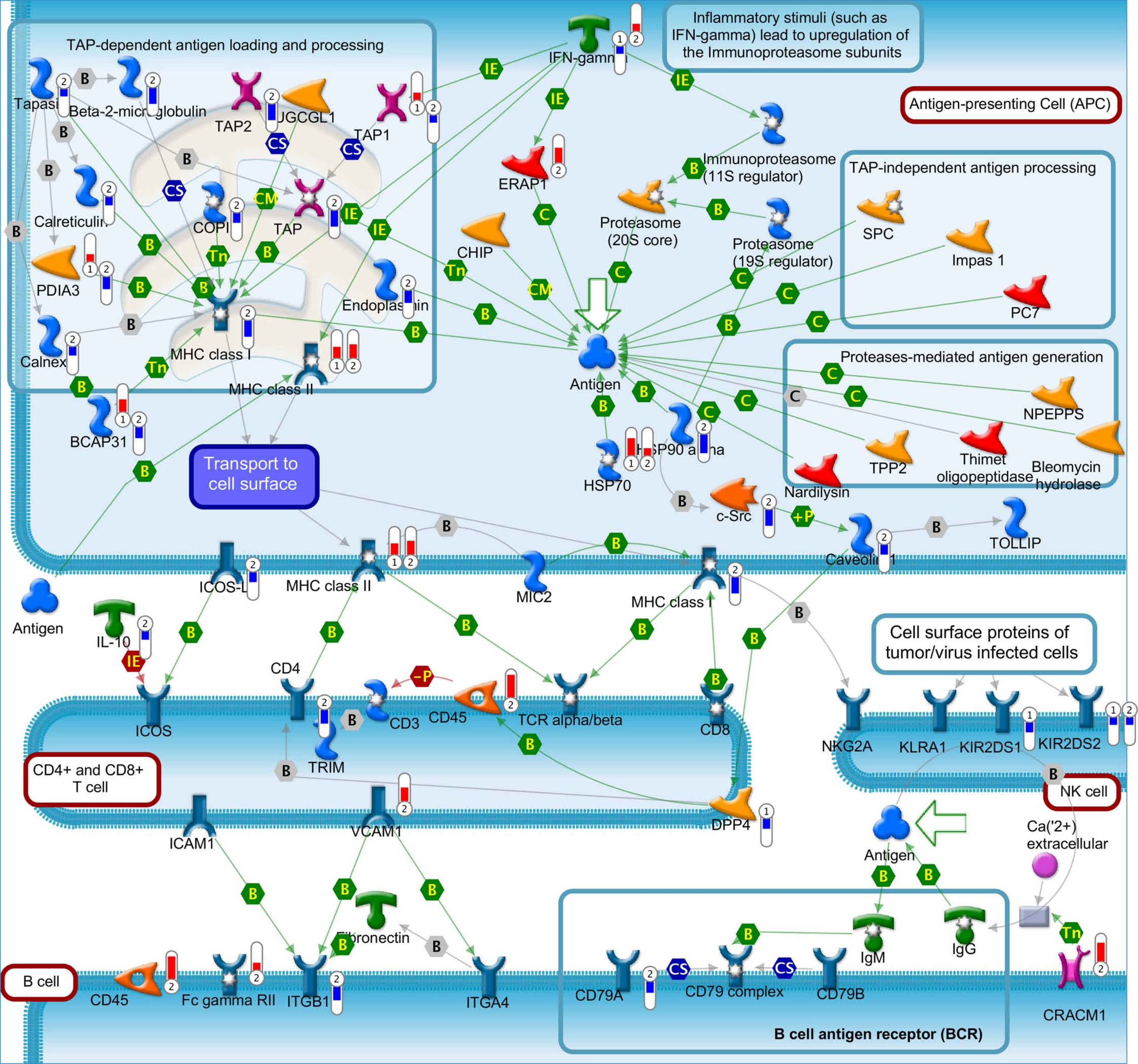

TAP-dependent antigen loading and processing
Inflammatory stimuli (such as IFN-gamma) lead to upregulation of the Immunoproteasome subunits
Antigen-presenting Cell (APC)
TAP2
TAP1
TAP
Calreticulin
PDIA3
COPI
MHC class I
MHC class II
BCAP31
ERAP1
CHIP
Immunoproteasome (11S regulator)
Proteasome (20S core)
Proteasome (19S regulator)
TAP-independent antigen processing
SPC
Impas 1
PC7
Antigen
Proteases-mediated antigen generation
NPEPPS
TPP2
Thimet oligopeptidase
Bleomycin hydrolase
Nardilysin
HSP70
c-Src
TOLLIP
Transport to cell surface
MIC2
IL-10
ICOS
CD4
CD3
CD45
TCR alpha/beta
CD8
TRIM
NKG2A
KLRA1
KIR2DS1
KIR2DS2
Cell surface proteins of tumor/virus infected cells
CD4+ and CD8+ T cell
NK cell
ICAM1
VCAM1
DPP4
Ca('2+) extracellular
IgM
IgG
B cell
Fc gamma RII
ITGB1
ITGA4
CD79A
CD79 complex
CD79B
CRACM1
B cell antigen receptor (BCR)

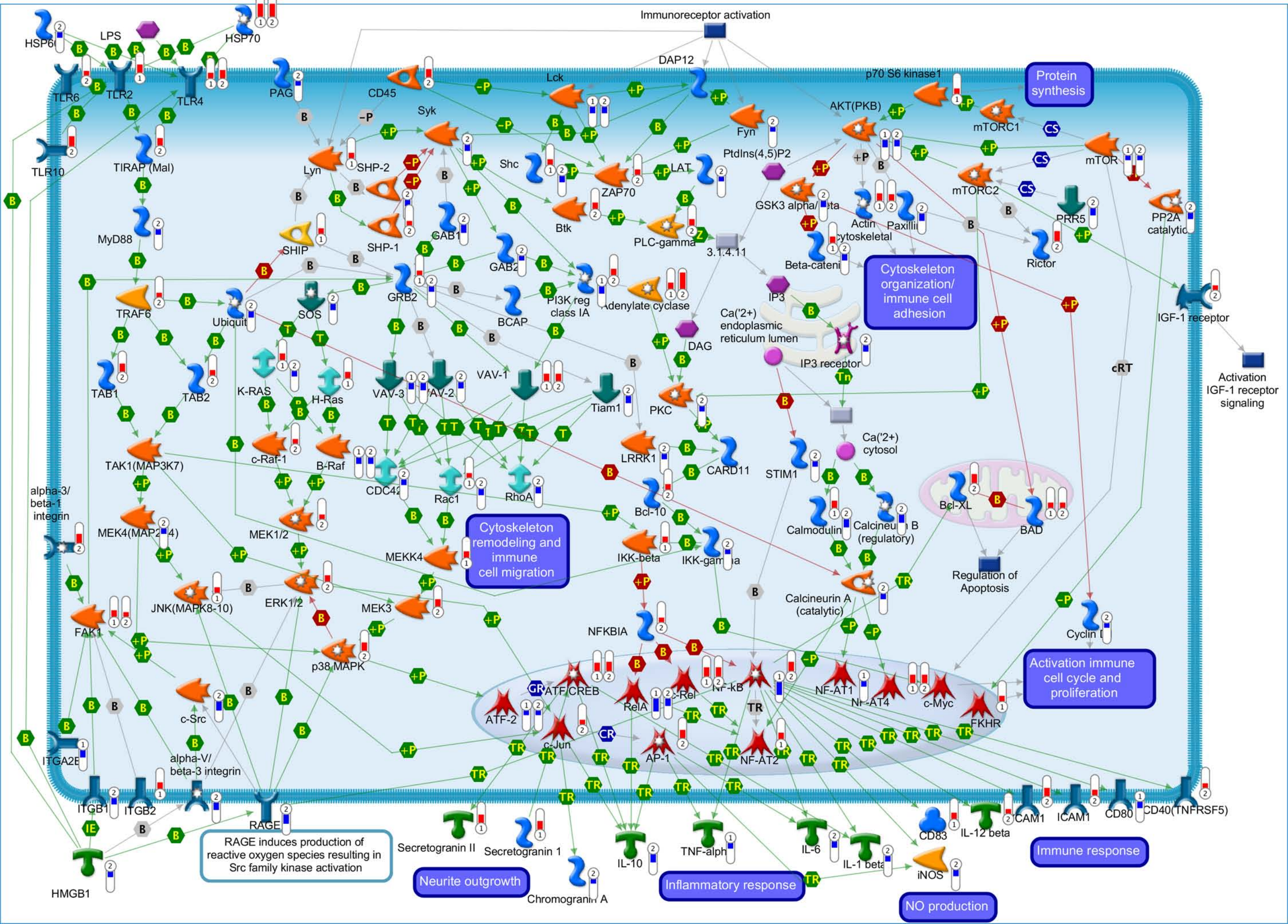
Immunoreceptor activation
HSP60
LPS
HSP70
TLR6
TLR2
TLR4
TLR10
PAG
CD45
DAP12
Lck
Syk
Fyn
p70 S6 kinase1
Protein synthesis
AKT(PKB)
mTORC1
mTOR
mTORC2
PRR5
PP2A catalytic
Rictor
TIRAP (Mal)
MyD88
Lyn
SHP-2
SHP-1
SHIP
Shc
ZAP70
LAT
Btk
PLC-gamma
GAB1
GAB2
PtdIns(4,5)P2
GSK3 alpha/beta
Actin cytoskeletal
Paxillin
Beta-catenin
3.1.4.11
IP3
Cytoskeleton organization/ immune cell adhesion
IGF-1 receptor
Activation IGF-1 receptor signaling
TRAF6
Ubiquitin
SOS
GRB2
BCAP
PI3K reg class IA
Adenylate cyclase
DAG
Ca('2+) endoplasmic reticulum lumen
IP3 receptor
TAB1
TAB2
K-RAS
H-Ras
VAV-3
VAV-2
VAV-1
Tiam1
PKC
Ca('2+) cytosol
TAK1(MAP3K7)
c-Raf-1
B-Raf
CDC42
Rac1
RhoA
LRRK1
CARD11
STIM1
alpha-3/ beta-1 integrin
MEK4(MAP2K4)
MEK1/2
MEKK4
Cytoskeleton remodeling and immune cell migration
Bcl-10
IKK-beta
IKK-gamma
Calmodulin
Calcineurin B (regulatory)
Bcl-XL
BAD
Regulation of Apoptosis
JNK(MAPK8-10)
ERK1/2
MEK3
FAK1
Calcineurin A (catalytic)
NFKBIA
Cyclin D
p38 MAPK
c-Src
ATF-2
ATF/CREB
c-Jun
RelA
c-Rel
NF-kB
AP-1
NF-AT1
NF-AT4
NF-AT2
c-Myc
FKHR
Activation immune cell cycle and proliferation
ITGA2B
ITGB1
ITGB2
alpha-V/ beta-3 integrin
RAGE
RAGE induces production of reactive oxygen species resulting in Src family kinase activation
HMGB1
Secretogranin II
Secretogranin 1
Chromogranin A
IL-10
TNF-alpha
IL-6
IL-1 beta
iNOS
CD83
IL-12 beta
VCAM1
ICAM1
CD80
CD40(TNFRSF5)
Neurite outgrowth
Inflammatory response
NO production
Immune response

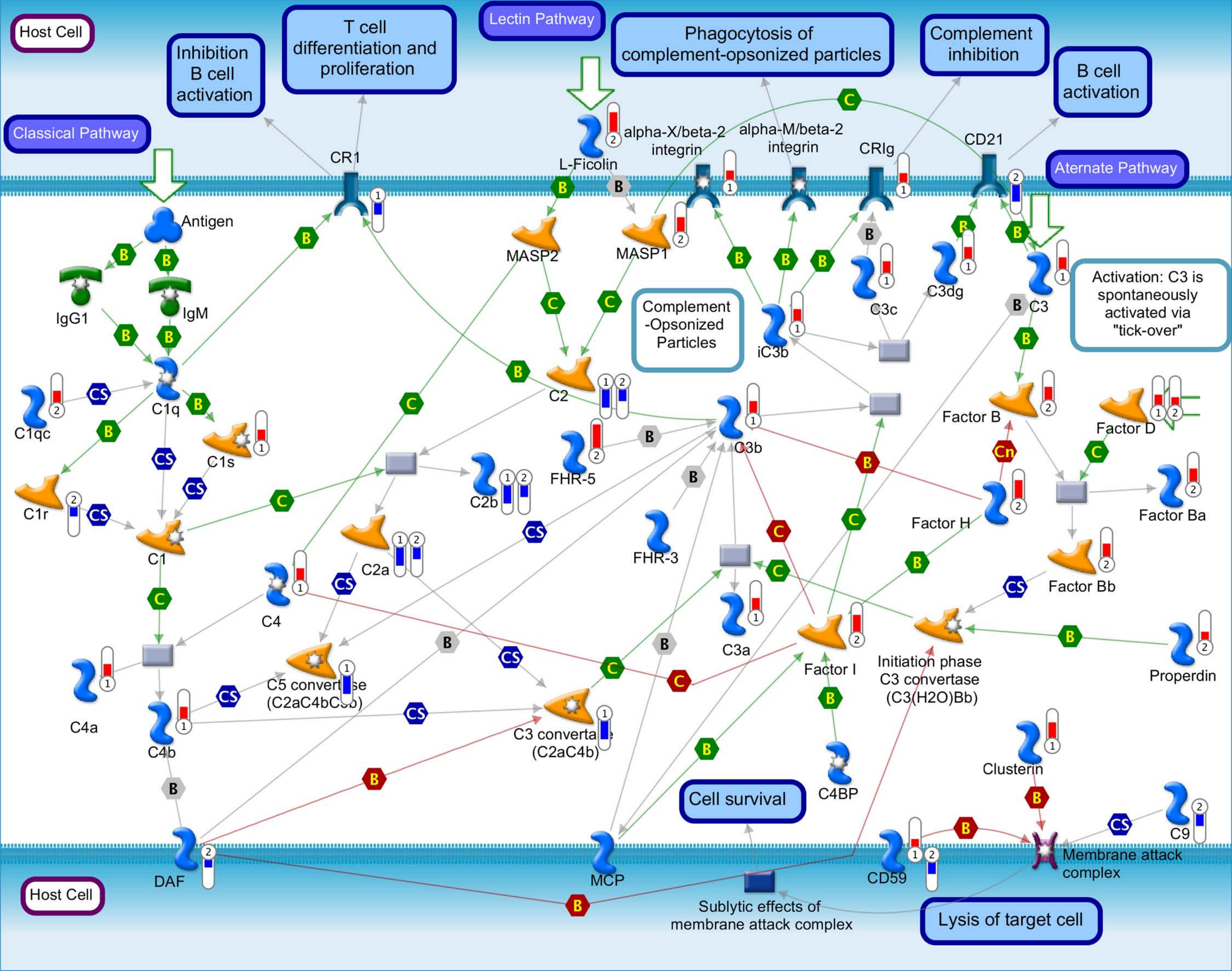
Host Cell
Inhibition B cell activation
T cell differentiation and proliferation
Lectin Pathway
Phagocytosis of complement-opsonized particles
Complement inhibition
B cell activation
Classical Pathway
CR1
alpha-X/beta-2 integrin
alpha-M/beta-2 integrin
CRIg
CD21
L-Ficolin
Aternate Pathway
Antigen
MASP2
MASP1
IgG1
IgM
C3c
C3dg
C3
Activation: C3 is spontaneously activated via "tick-over"
Complement -Opsonized Particles
iC3b
C1qc
C1q
C1s
C2
C3b
Factor B
Factor D
FHR-5
C2b
C1r
C1
Factor H
Factor Ba
FHR-3
C2a
C4
C3a
Factor Bb
Factor I
Initiation phase C3 convertase (C3(H2O)Bb)
Properdin
C4a
C5 convertase (C2aC4bC3b)
C4b
C3 convertase (C2aC4b)
Clusterin
C4BP
Cell survival
C9
DAF
MCP
CD59
Membrane attack complex
Sublytic effects of membrane attack complex
Lysis of target cell

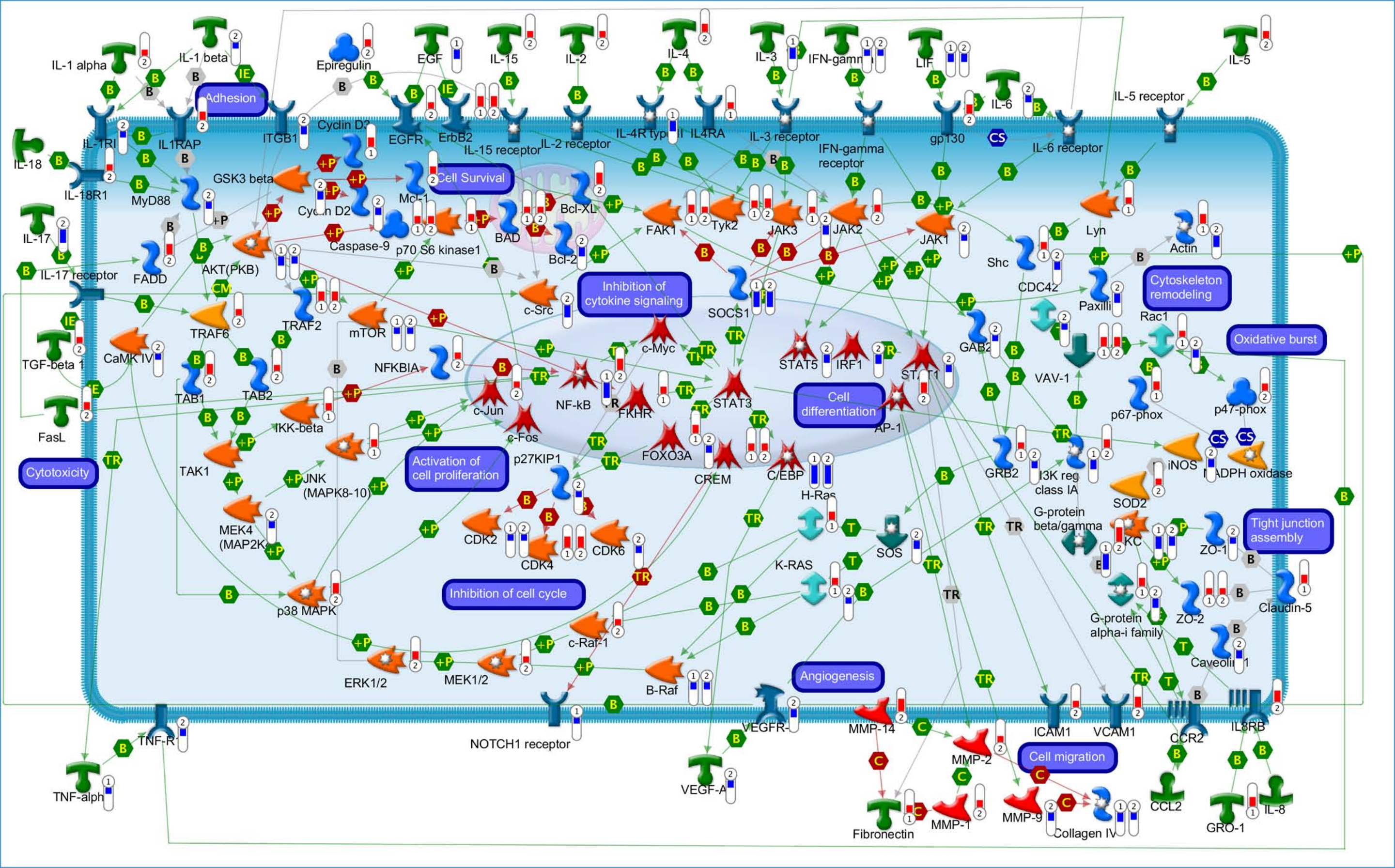
IL-1 alpha
IL-1 beta
Adhesion
Epiregulin
EGF
IL-15
IL-2
IL-4
IL-3
IFN-gamm
LIF
IL-6
IL-5
IL-5 receptor
IL-1RI
IL1RAP
ITGB1
EGFR
ErbB2
IL-15 receptor
IL-2 receptor
IL-4R typ
IL4RA
IL-3 receptor
IFN-gamma receptor
gp130
IL-6 receptor
IL-18
IL-18R1
MyD88
GSK3 beta
Cyclin D2
Cell Survival
Mcl-1
Bcl-XL
IL-17
AKT(PKB)
Caspase-9
p70 S6 kinase1
BAD
Bcl-2
FAK1
Tyk2
JAK3
JAK2
JAK1
Lyn
Actin
Shc
CDC42
Paxilli
Cytoskeleton remodeling
IL-17 receptor
FADD
TRAF2
c-Src
Inhibition of cytokine signaling
SOCS1
Rac1
Oxidative burst
TGF-beta 1
CaMK IV
TRAF6
mTOR
c-Myc
STAT5
IRF1
GAB2
TAB1
TAB2
NFKBIA
NF-kB
FKHR
STAT3
VAV-1
p67-phox
p47-phox
FasL
IKK-beta
c-Jun
c-Fos
Cell differentiation
AP-1
iNOS
ADPH oxidase
Cytotoxicity
TAK1
Activation of cell proliferation
p27KIP1
FOXO3A
CREM
C/EBP
H-Ras
GRB2
I3K reg class IA
SOD2
JNK (MAPK8-10)
MEK4 (MAP2K
CDK2
CDK4
CDK6
G-protein beta/gamma
KC
ZO-1
Tight junction assembly
SOS
K-RAS
Inhibition of cell cycle
p38 MAPK
c-Raf-1
ZO-2
Claudin-5
G-protein alpha-i family
Caveoli
ERK1/2
MEK1/2
B-Raf
Angiogenesis
TNF-R
NOTCH1 receptor
VEGFR-
MMP-14
ICAM1
VCAM1
CCR2
IL8RB
MMP-2
Cell migration
TNF-alph
VEGF-A
Fibronectin
MMP-1
MMP-9
Collagen IV
CCL2
GRO-1
IL-8

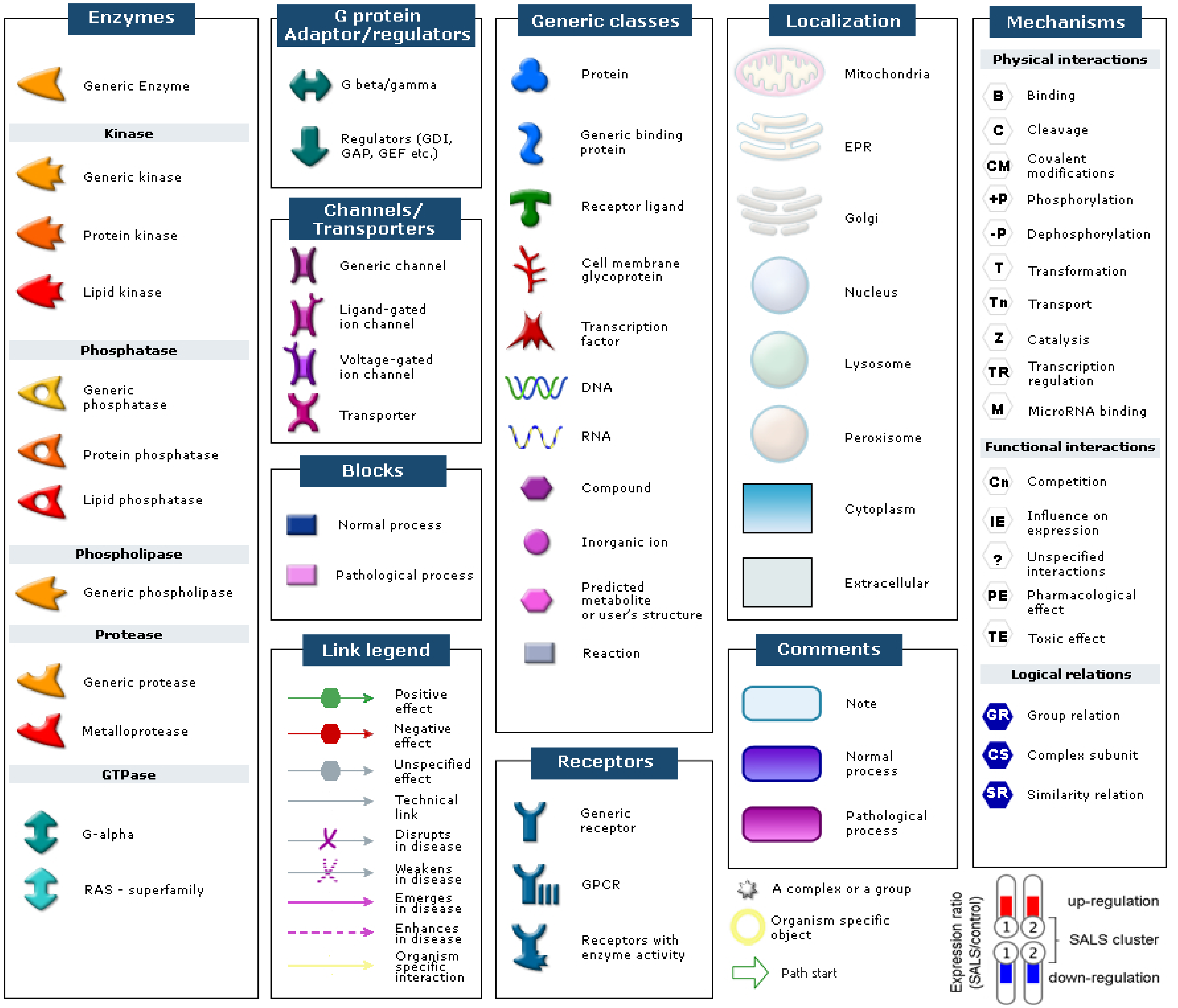
Enzymes
Generic Enzyme
Kinase
Generic kinase
Protein kinase
Lipid kinase
Phosphatase
Generic phosphatase
Protein phosphatase
Lipid phosphatase
Phospholipase
Generic phospholipase
Protease
Generic protease
Metalloprotease
GTPase
G-alpha
RAS - superfamily
G protein Adaptor/regulators
G beta/gamma
Regulators (GDI, GAP, GEF etc.)
Channels/ Transporters
Generic channel
Ligand-gated ion channel
Voltage-gated ion channel
Transporter
Blocks
Normal process
Pathological process
Link legend
Positive effect
Negative effect
Unspecified effect
Technical link
Disrupts in disease
Weakens in disease
Emerges in disease
Enhances in disease
Organism specific interaction
Generic classes
Protein
Generic binding protein
Receptor ligand
Cell membrane glycoprotein
Transcription factor
DNA
RNA
Compound
Inorganic ion
Predicted metabolite or user's structure
Reaction
Receptors
Generic receptor
GPCR
Receptors with enzyme activity
Localization
Mitochondria
EPR
Golgi
Nucleus
Lysosome
Peroxisome
Cytoplasm
Extracellular
Comments
Note
Normal process
Pathological process
A complex or a group
Organism specific object
Path start
Mechanisms
Physical interactions
B Binding
C Cleavage
CM Covalent modifications
+P Phosphorylation
-P Dephosphorylation
T Transformation
Tn Transport
Z Catalysis
TR Transcription regulation
M MicroRNA binding
Functional interactions
Cn Competition
IE Influence on expression
? Unspecified interactions
PE Pharmacological effect
TE Toxic effect
Logical relations
GR Group relation
CS Complex subunit
SR Similarity relation
Expression ratio (SALS/control)
up-regulation
1 2
SALS cluster
1 2
down-regulation

Supplementary Table 1. Pathway enrichment analysis on the entire list of statistically deregulated genes in SALS patients versus controls.

#	Maps	pValue	FDR	Ratio
1	Thrombopoetin signaling via JAK-STAT pathway	2.591E-04	3.388E-02	3/22
2	**Huntingtin-depended transcription deregulation in Huntington's Disease**	3.378E-04	3.388E-02	3/24
3	**Immune response_IL-4-induced regulators of cell growth, survival, differentiation and metabolism**	4.600E-04	3.388E-02	4/63
4	**Signal transduction_JNK pathway**	2.463E-03	1.361E-01	3/47
5	Prolactin receptor signaling	4.483E-03	1.781E-01	3/58
6	**Cytokines and Chemokines signaling**	6.075E-03	1.781E-01	4/127
7	**Immune response_Oncostatin M signaling via JAK-Stat**	7.008E-03	1.781E-01	2/22
8	**Immune response_IL-6 signaling pathway via JAK/STAT**	8.186E-03	1.781E-01	3/72
9	**Role of Parkin in the Ubiquitin-Proteasomal Pathway**	8.311E-03	1.781E-01	2/24
10	Glucocorticoid receptor signaling	9.001E-03	1.781E-01	2/25
11	Mitogenic action of Estradiol / ESR1 (nuclear) in breast cancer	1.122E-02	1.781E-01	2/28
12	Cell cycle_Role of SCF complex in cell cycle regulation	1.200E-02	1.781E-01	2/29
13	Role of Diethylhexyl Phthalate and Tributyltin in fat cell differentiation	1.200E-02	1.781E-01	2/29
14	Development_G-CSF-induced myeloid differentiation	1.281E-02	1.781E-01	2/30
15	**PTMs in IL-23 signaling pathway**	1.365E-02	1.781E-01	2/31
16	Putative pathways for stimulation of fat cell differentiation by Bisphenol A	1.450E-02	1.781E-01	2/32
17	Development_Transcription regulation of granulocyte development	1.450E-02	1.781E-01	2/32
18	Oxidative stress_Role of ASK1 under oxidative stress	1.628E-02	1.811E-01	2/34
19	**Growth hormone signaling via STATs and PLC/IP3**	1.721E-02	1.811E-01	2/35
20	Role of growth factor receptors transactivation by Hyaluronic acid / CD44 signaling in tumor progression	1.721E-02	1.811E-01	2/35
21	**EPO-induced Jak-STAT pathway**	1.815E-02	1.824E-01	2/36
22	Signal transduction_Cyclic AMP signaling	2.011E-02	1.852E-01	2/38
23	Cell cycle_Regulation of G1/S transition (part 1)	2.011E-02	1.852E-01	2/38
24	**TNF-alpha-induced Caspase-8 signaling**	2.536E-02	2.167E-01	2/43
25	**Immune response_IL-13 signaling via JAK-STAT**	2.647E-02	2.167E-01	2/44
26	**Apoptosis and survival_FAS signaling cascades**	2.647E-02	2.167E-01	2/44
27	**Signal transduction_PTMs in IL-12 signaling pathway**	3.111E-02	2.455E-01	2/48
28	**TGF-beta receptor signaling**	3.353E-02	2.556E-01	2/50
29	**Signal transduction_PKA signaling**	3.478E-02	2.562E-01	2/51
30	Development_Differentiation of white adipocytes	3.731E-02	2.660E-01	2/53
31	Tricarbonic acid cycle	4.125E-02	2.762E-01	2/56
32	Development_Keratinocyte differentiation	4.125E-02	2.762E-01	2/56
33	**Immune response_IL-5 signaling via JAK/STAT**	4.259E-02	2.768E-01	2/57
34	IGF family signaling in colorectal cancer	4.672E-02	2.869E-01	2/60
35	Regulation of lipid metabolism_G-alpha(q) regulation of lipid metabolism	4.813E-02	2.869E-01	2/61
36	Cell adhesion_Role of CDK5 in cell adhesion	5.073E-02	2.869E-01	1/9
37	**Immune response_IFN-alpha/beta signaling via JAK/STAT**	5.245E-02	2.869E-01	2/64
38	Renal tubulointerstitial injury in Lupus Nephritis	5.392E-02	2.869E-01	2/65
39	Ligand-independent activation of Androgen receptor in Prostate Cancer	5.690E-02	2.869E-01	2/67
40	Development_Role of nicotinamide in G-CSF-induced granulopoiesis	6.707E-02	3.294E-01	1/12
41	**Immune response_IFN-alpha/beta signaling via MAPKs**	7.263E-02	3.417E-01	2/77
42	Cell cycle_Nucleocytoplasmic transport of CDK/Cyclins	7.781E-02	3.417E-01	1/14
43	Glycolysis and gluconeogenesis	8.958E-02	3.417E-01	2/87
44	DNA damage_Role of SUMO in p53 regulation	9.369E-02	3.417E-01	1/17
45	GTP-XTP metabolism	9.487E-02	3.417E-01	2/90
46	Transcription_Assembly of RNA Polymerase II preinitiation complex on TATA-less promoters	9.892E-02	3.417E-01	1/18
47	**Immune response_IL-3 signaling via JAK/STAT, p38, JNK and NF-kB**	1.003E-01	3.417E-01	2/93
48	**Immune response_IFN-alpha/beta signaling via PI3K and NF-kB pathways**	1.021E-01	3.417E-01	2/94
49	**NOTCH-induced EMT**	1.041E-01	3.417E-01	1/19
50	Protein folding_Membrane trafficking and signal transduction of G-alpha (i) heterotrimeric G-protein	1.041E-01	3.417E-01	1/19

Supplementary Table 2. Pathway enrichment analysis on the entire list of statistically deregulated genes in SALS1 (n=4485) and SALS2 (16144) patients compared to controls.

#	Maps	pValue	min(pValue)	FDR	Ratio
1	Cytoskeleton remodeling_TGF, WNT and cytoskeletal remodeling	2.54E-09<br>3.15E-13	3.15E-13	3.70E-07<br>3.54E-11	37/111<br>72/111
2	Cytoskeleton remodeling_Cytoskeleton remodeling	1.70E-06<br>3.63E-12	3.63E-12	9.52E-05<br>3.63E-10	30/102<br>66/102
3	Transport_Clathrin-coated vesicle cycle	8.65E-03<br>3.38E-10	3.38E-10	3.69E-02<br>2.77E-08	16/71<br>48/71
4	**Signal transduction_mTORC1 downstream signaling**	5.61E-05<br>1.82E-09	1.82E-09	1.26E-03<br>1.37E-07	19/61<br>42/61
5	**Normal and pathological TGF-beta-mediated regulation of cell proliferation**	1.10E-03<br>3.05E-09	3.05E-09	9.62E-03<br>2.10E-07	14/33<br>27/33
6	Development_Positive regulation of STK3/4 (Hippo) pathway and negative regulation of YAP/TAZ function	1.17E-03<br>3.26E-09	3.26E-09	9.87E-03<br>2.10E-07	18/70<br>46/70
7	Neurogenesis_NGF/ TrkA MAPK-mediated signaling	4.23E-02<br>3.79E-09	3.79E-09	1.13E-01<br>2.12E-07	19/105<br>62/105
8	Development_Regulation of cytoskeleton proteins in oligodendrocyte differentiation and myelination	2.55E-05<br>4.13E-09	4.13E-09	6.75E-04<br>2.12E-07	19/58<br>40/58
9	Neurophysiological process_Dynein-dynactin motor complex in axonal transport in neurons	3.88E-07<br>4.14E-09	4.14E-09	3.77E-05<br>2.12E-07	21/54<br>38/54
10	**Immune response_M-CSF-receptor signaling pathway**	9.98E-02<br>4.24E-09	4.24E-09	2.08E-01<br>2.12E-07	14/81<br>51/81
11	**Cytokine-mediated regulation of megakaryopoiesis**	2.30E-03<br>9.20E-09	9.20E-09	1.52E-02<br>4.36E-07	15/57<br>39/57
12	**G-protein signaling_RhoA regulation pathway**	1.04E-01<br>1.04E-08	1.04E-08	2.15E-01<br>4.44E-07	7/34<br>27/34
13	**Chemotaxis_CXCR4 signaling pathway**	5.13E-03<br>1.04E-08	1.04E-08	2.55E-02<br>4.44E-07	10/34<br>27/34
14	Development_WNT signaling pathway. Part 1. Degradation of beta-catenin in the absence WNT signaling	6.79E-02<br>1.12E-08	1.12E-08	1.54E-01<br>4.56E-07	5/19<br>18/19
15	Apoptosis and survival_NGF/ TrkA PI3K-mediated signaling	1.10E-07<br>1.94E-08	1.94E-08	1.21E-05<br>7.35E-07	27/77<br>48/77
16	Development_Prolactin receptor signaling	3.96E-02<br>1.96E-08	1.96E-08	1.08E-01<br>7.35E-07	12/58<br>39/58
17	**Immune response_IL-3 signaling via ERK and PI3K**	1.84E-03<br>2.60E-08	2.60E-08	1.30E-02<br>9.17E-07	23/102<br>59/102
18	LRRK2 in neurons in Parkinson's disease	1.74E-06<br>2.65E-08	2.65E-08	9.52E-05<br>9.17E-07	15/33<br>26/33
19	**Cell adhesion_Chemokines and adhesion**	1.07E-06<br>2.98E-08	2.98E-08	7.81E-05<br>9.94E-07	30/100<br>58/100
20	**Immune response_Oncostatin M signaling via MAPK**	1.47E-01<br>3.65E-08	3.65E-08	2.73E-01<br>1.17E-06	7/37<br>28/37
21	**Immune response_IL-6 signaling pathway via JAK/STAT**	4.20E-03<br>4.79E-08	4.79E-08	2.24E-02<br>1.49E-06	17/72<br>45/72
22	**Signal transduction_mTORC2 downstream signaling**	5.60E-03<br>5.80E-08	5.80E-08	2.69E-02<br>1.74E-06	16/68<br>43/68
23	Transcription_Transcription regulation of aminoacid metabolism	1.72E-01<br>7.82E-08	7.82E-08	2.99E-01<br>2.27E-06	5/25<br>21/25
24	**Immune response_Antigen presentation by MHC class I, classical pathway**	1.27E-03<br>9.68E-08	9.68E-08	1.05E-02<br>2.72E-06	15/54<br>36/54
25	**Signal transduction_Activation of PKC via G-Protein coupled receptor**	8.30E-04<br>1.01E-07	1.01E-07	8.54E-03<br>2.76E-06	15/52<br>35/52
26	**Immune response_Role of PKR in stress-induced antiviral cell response**	7.83E-04<br>1.83E-07	1.83E-07	8.25E-03<br>4.83E-06	16/57<br>37/57
27	Tau pathology in Alzheimer disease	2.71E-02<br>1.96E-07	1.96E-07	8.30E-02<br>5.04E-06	12/55<br>36/55
28	**Immune response_HMGB1/RAGE signaling pathway**	8.31E-03<br>2.09E-07	2.09E-07	3.60E-02<br>5.21E-06	13/53<br>35/53
29	**IL-6 signaling in multiple myeloma**	1.53E-02<br>2.20E-07	2.20E-07	5.37E-02<br>5.36E-06	12/51<br>34/51
30	Regulation of degradation of deltaF508-CFTR in CF	2.10E-05<br>2.41E-07	2.41E-07	6.12E-04<br>5.71E-06	15/39<br>28/39
31	**Immune response_IL-3 signaling via JAK/STAT, p38, JNK and NF-kB**	7.27E-06<br>2.58E-07	2.58E-07	2.89E-04<br>5.94E-06	27/93<br>53/93
32	**Aberrant production of IL-2 and IL-17 in SLE T cells**	9.63E-04<br>3.53E-07	3.53E-07	9.06E-03<br>7.85E-06	16/58<br>37/58
33	DeltaF508-CFTR traffic / Sorting endosome formation in CF	6.69E-01<br>3.57E-07	3.57E-07	8.24E-01<br>7.85E-06	22/28<br>3/28
34	**Immune response_IL-4 signaling pathway**	5.61E-04<br>4.12E-07	4.12E-07	6.55E-03<br>8.82E-06	23/94<br>53/94
35	Ligand-independent activation of Androgen receptor in Prostate Cancer	5.70E-06<br>4.33E-07	4.33E-07	2.52E-04<br>9.06E-06	22/67<br>41/67
36	Development_TGF-beta-dependent induction of EMT via RhoA, PI3K and ILK	4.59E-07<br>2.91E-02	4.59E-07	4.02E-05<br>5.22E-02	19/46<br>21/46
37	**Signal transduction_ERK1/2 signaling pathway**	1.06E-02<br>4.74E-07	4.74E-07	4.16E-02<br>9.44E-06	9/32<br>24/32
38	Development_Adenosine A2B receptor signaling	1.68E-03<br>4.75E-07	4.75E-07	1.25E-02<br>9.44E-06	14/50<br>33/50
39	**Immune response_Antigen presentation by MHC class I: cross-presentation**	4.97E-04<br>4.82E-07	4.82E-07	6.26E-03<br>9.44E-06	24/99<br>55/99
40	Development_Epigenetic and transcriptional regulation of oligodendrocyte precursor cell differentiation and myelination	1.45E-03<br>5.15E-07	5.15E-07	1.11E-02<br>9.81E-06	11/34<br>25/34
41	**Immune response_IL-6 signaling pathway via MEK/ERK and PI3K/AKT cascades**	5.65E-03<br>5.26E-07	5.26E-07	2.69E-02<br>9.81E-06	17/74<br>44/74
42	Development_EPO-induced Jak-STAT pathway	5.90E-02<br>5.43E-07	5.43E-07	1.40E-01<br>9.81E-06	8/36<br>26/36
43	K-RAS signaling in pancreatic cancer	7.34E-02<br>5.45E-07	5.45E-07	1.64E-01<br>9.81E-06	9/44<br>30/44
44	Action of GSK3 beta in bipolar disorder	2.94E-01<br>5.65E-07	5.65E-07	4.44E-01<br>9.98E-06	4/23<br>19/23
45	Some pathways of EMT in cancer cells	6.25E-07<br>[illegible]	6.25E-07	4.97E-05<br>[illegible]	20/51<br>[illegible]

45	Some pathways of EMT in cancer cells	1.34E-05	[illegible]	1.15E-04	31/51
46	Neurophysiological process_GABA-A receptor life cycle	3.57E-02<br>9.04E-07	9.04E-07	1.01E-01<br>1.57E-05	7/27<br>21/27
47	Development_Regulation of epithelial-to-mesenchymal transition (EMT)	9.38E-06<br>9.63E-07	9.63E-07	3.33E-04<br>1.64E-05	21/64<br>39/64
48	**Signal transduction_JNK pathway**	7.90E-03<br>1.08E-06	1.08E-06	3.48E-02<br>1.80E-05	12/47<br>31/47
49	Cell adhesion_Histamine H1 receptor signaling in the interruption of cell barrier integrity	1.79E-03<br>1.15E-06	1.15E-06	1.30E-02<br>1.88E-05	13/45<br>30/45
50	Cytoskeleton remodeling_Hyaluronic acid/ CD44 signaling pathways	3.64E-03<br>1.20E-06	1.20E-06	2.04E-02<br>1.90E-05	12/43<br>29/43

Supplementary Table 3. Neuroinflammatory genes differentially expressed in SALS1.

Probe ID	p Value (Corr)	p Value	Fold Change (SALS1/CTRL^{+})*	GENE_SYMBOL
A_23_P166051	2.08E-02	1.18E-02	1.47	RBCK1
A_23_P329573	3.22E-02	1.93E-02	2.17	ITGB2
A_24_P343233	1.44E-05	1.23E-06	2.20	HLA-DRB1
A_23_P89249	7.67E-04	2.59E-04	1.99	ERBB2
A_23_P135769	6.60E-05	1.21E-05	3.04	ACTB
A_23_P24870	3.85E-02	2.38E-02	2.05	CD44
A_23_P309739	2.75E-02	1.61E-02	-1.46	ESR1
A_24_P362193	2.48E-05	2.98E-06	-3.52	CD84
A_23_P158725	5.34E-05	9.11E-06	1.76	SLC16A3
A_23_P145089	1.26E-06	9.38E-09	3.55	HSP90AB1
A_24_P50759	4.10E-06	1.22E-07	-1.40	**TNF**
A_23_P75283	1.02E-04	2.16E-05	1.93	RBP4
A_23_P4223	2.99E-06	5.79E-08	2.02	CALCOCO2
A_23_P79591	2.15E-06	3.16E-08	-2.16	APOB
A_23_P29495	3.09E-05	4.13E-06	2.02	CTNNB1
A_24_P161933	7.60E-04	2.56E-04	3.87	HLA-B
A_23_P315571	6.84E-05	1.27E-05	2.10	RFTN1
A_23_P120947	7.38E-06	3.33E-07	3.05	XRCC6
A_23_P369237	2.37E-02	1.37E-02	-1.39	ADIPOQ
A_23_P213114	1.46E-03	5.55E-04	-4.41	TEC
A_24_P281101	8.16E-06	4.17E-07	-1.91	ABL1
A_23_P70095	1.39E-02	7.41E-03	1.69	CD74
A_23_P101992	3.31E-02	1.99E-02	2.44	MARCO
A_23_P147875	2.88E-05	3.72E-06	2.12	CHID1
A_23_P156788	1.16E-02	6.02E-03	-1.59	STX11
A_24_P123616	3.81E-06	1.01E-07	4.67	HSPA1A
A_23_P70539	2.05E-02	1.16E-02	2.42	HLA-C
A_24_P7887	2.54E-04	6.75E-05	-1.79	EP300
A_23_P103361	1.17E-02	6.08E-03	-1.48	LCK
A_24_P943283	1.43E-03	5.37E-04	-1.64	DENND1B
A_23_P60387	3.63E-02	2.23E-02	-1.37	NOTCH1
A_23_P206510	9.25E-06	5.80E-07	1.80	GLG1
A_23_P202156	4.66E-02	2.99E-02	-1.47	NFKB2
A_23_P61646	2.05E-02	1.16E-02	1.81	STX4
A_23_P29036	5.59E-05	9.69E-06	1.71	IFNGR2
A_24_P390495	1.27E-02	6.69E-03	1.62	CX3CL1
A_24_P254079	6.81E-03	3.31E-03	2.10	ICAM5
A_23_P215913	1.03E-05	6.92E-07	3.90	CLU
A_23_P98183	1.68E-03	6.55E-04	2.13	HRAS
A_24_P360674	8.53E-06	4.75E-07	-4.42	CDKN2B
A_23_P81912	5.03E-06	1.64E-07	2.33	TUBB
A_23_P151294	1.01E-06	3.77E-09	-1.80	IFNG
A_23_P92499	1.86E-03	7.38E-04	2.06	TLR2
A_24_P287043	8.52E-05	1.70E-05	2.49	IFITM2
A_23_P99452	5.88E-06	2.02E-07	-1.75	BRCA2
A_32_P139894	1.91E-05	1.93E-06	1.74	ABL2

A_23_P65442	7.26E-05	1.37E-05	1.86	IRF9
A_24_P28295	1.44E-04	3.29E-05	1.97	RABGAP1L
A_24_P175059	6.57E-04	2.16E-04	1.48	ATG5
A_23_P140916	4.24E-06	1.30E-07	2.17	GLG1
A_23_P410998	2.60E-02	1.52E-02	2.34	RAB5B
A_32_P78528	7.74E-06	3.69E-07	2.89	TUBB
A_23_P428738	3.46E-03	1.52E-03	1.56	**ANG**
A_23_P29994	8.12E-06	4.08E-07	2.08	RBPJ
A_32_P204381	2.72E-03	1.16E-03	2.00	CIAPIN1
A_23_P101683	2.49E-02	1.45E-02	-1.96	CLC
A_23_P26777	1.16E-03	4.21E-04	2.02	NUP85
A_23_P42935	1.37E-02	7.30E-03	-1.32	BRAF
A_23_P57927	1.89E-02	1.06E-02	1.59	HYAL2
A_32_P120977	8.16E-06	4.15E-07	1.64	PRELID1
A_24_P134683	1.12E-05	7.84E-07	3.38	BCAP31
A_23_P6836	3.63E-02	2.23E-02	3.23	IP6K2
A_24_P69654	2.22E-06	3.44E-08	2.51	KLF6
A_24_P304549	1.55E-02	8.47E-03	3.13	LAMP1
A_23_P358370	3.21E-02	1.93E-02	-1.93	FOXE1
A_32_P194062	1.52E-05	1.37E-06	-7.05	TYR
A_23_P121253	1.57E-02	8.57E-03	-1.40	TNFSF10
A_23_P145264	9.01E-06	5.46E-07	2.63	HLA-F
A_23_P81241	1.41E-05	1.17E-06	1.91	NDFIP1
A_23_P408353	4.10E-05	6.25E-06	2.70	HLA-A
A_23_P167493	3.31E-05	4.66E-06	2.80	CARTPT
A_23_P25235	3.72E-03	1.65E-03	-2.93	CLEC4D
A_23_P501232	5.03E-04	1.58E-04	1.94	KCNAB2
A_23_P134100	6.34E-03	3.05E-03	-1.78	ULBP3
A_24_P214506	4.82E-03	2.23E-03	2.64	SEC13
A_23_P113716	7.07E-06	2.91E-07	2.11	HLA-C
A_23_P205746	9.69E-06	6.30E-07	1.71	EML1
A_23_P252471	2.73E-06	5.12E-08	1.98	PECAM1
A_24_P48078	4.12E-04	1.23E-04	1.76	CYLD
A_23_P39040	2.58E-02	1.50E-02	2.48	SMARCA4
A_32_P157228	2.00E-02	1.13E-02	-3.63	AHCTF1
A_24_P15270	1.64E-05	1.53E-06	2.10	BCR
A_23_P375147	1.71E-04	4.10E-05	1.75	RC3H2
A_24_P235643	3.55E-04	1.02E-04	2.09	CUEDC2
A_23_P46844	2.34E-03	9.65E-04	1.72	TRIM8
A_23_P68511	9.72E-04	3.44E-04	-3.63	ANGPT4
A_23_P147431	8.91E-04	3.09E-04	1.52	LYN
A_23_P130815	3.40E-03	1.49E-03	-1.84	KIR2DS2
A_24_P229055	2.37E-02	1.37E-02	2.10	SEMA4D
A_23_P55802	1.94E-05	1.97E-06	1.48	AP1M1
A_23_P143178	6.00E-03	2.86E-03	-3.71	LBP
A_23_P217866	2.20E-04	5.58E-05	1.84	IFI16
A_24_P276853	1.29E-03	4.79E-04	2.01	GON4L
A_23_P314024	3.40E-05	4.87E-06	2.47	HLA-F
A_23_P160720	1.38E-03	5.16E-04	1.62	BATF3
A_24_P252078	1.81E-02	1.01E-02	1.63	BTN3A2

A_24_P766716	2.55E-02	1.48E-02	1.72	CMKLR1
A_23_P250825	5.40E-03	2.54E-03	1.56	RABGEF1
A_23_P162918	1.30E-02	6.90E-03	3.29	SERPINA3
A_24_P303091	8.31E-05	1.64E-05	-4.26	CXCL10
A_23_P207582	7.09E-06	2.99E-07	-3.23	CCL16
A_23_P57856	1.10E-04	2.38E-05	2.09	**BCL6**
A_24_P264664	2.43E-05	2.84E-06	2.05	CHD2
A_24_P363005	1.67E-06	1.73E-08	2.37	UBE2D3
A_24_P103469	4.54E-03	2.08E-03	1.98	LST1
A_24_P791515	1.66E-05	1.58E-06	1.93	SYNCRIP
A_23_P2873	1.17E-05	8.44E-07	2.69	KLC1
A_23_P214821	7.10E-04	2.37E-04	3.82	EDN1
A_23_P18490	1.66E-03	6.44E-04	1.89	MAEA
A_23_P164035	1.98E-05	2.04E-06	3.38	PSMC5
A_32_P232865	6.76E-03	3.28E-03	3.14	BCR
A_23_P25644	6.68E-07	8.25E-10	1.69	PCID2
A_24_P233960	1.19E-05	8.67E-07	2.27	SNX27
A_23_P39088	3.77E-04	1.10E-04	1.96	PRMT1
A_23_P303149	1.71E-04	4.10E-05	1.83	FGFR2
A_23_P252163	2.93E-02	1.74E-02	1.67	DAPK1
A_24_P245322	3.96E-03	1.77E-03	2.39	PRKAR1A
A_23_P79161	3.88E-06	1.11E-07	1.54	PRELID1
A_23_P166633	1.22E-05	9.06E-07	1.86	ITGB5
A_24_P87763	1.60E-04	3.77E-05	3.16	EEF2
A_24_P322635	1.93E-03	7.74E-04	2.20	ELMO2
A_23_P87379	1.67E-03	6.49E-04	2.46	PDE2A
A_23_P155632	3.65E-03	1.62E-03	-2.86	CD80
A_23_P28434	3.87E-04	1.14E-04	1.94	VAMP8
A_23_P104318	3.67E-05	5.37E-06	1.98	DDIT4
A_32_P72822	4.25E-05	6.53E-06	-2.65	CCNB2
A_23_P134237	1.09E-03	3.95E-04	2.77	RARRES2
A_23_P101960	4.41E-03	2.01E-03	2.72	ZFP36L2
A_23_P56195	6.68E-07	1.26E-09	1.58	UBA52
A_23_P435501	9.88E-05	2.07E-05	2.16	SERINC3
A_23_P55319	3.77E-04	1.10E-04	1.67	FLOT2
A_23_P203053	2.04E-03	8.20E-04	1.70	NCAM1
A_23_P30122	4.59E-06	1.48E-07	-1.83	IL2
A_24_P319354	1.79E-02	9.93E-03	2.06	SUMO1
A_24_P337657	1.45E-04	3.32E-05	1.90	SRF
A_24_P397043	1.49E-05	1.31E-06	-1.83	ST6GAL1
A_23_P169331	1.37E-02	7.28E-03	1.78	TRAF2
A_23_P203994	1.66E-06	1.49E-08	1.99	PSMD9
A_23_P47135	2.97E-05	3.88E-06	1.97	NR1H3
A_23_P130531	1.22E-02	6.39E-03	2.86	CDC37
A_24_P222655	1.03E-02	5.29E-03	2.90	C1QA
A_32_P70158	2.52E-02	1.46E-02	2.70	LILRB3
A_24_P391568	2.87E-02	1.70E-02	1.90	ARNT
A_32_P217709	4.24E-06	1.29E-07	2.37	RAC1
A_32_P231446	1.44E-05	1.22E-06	2.03	HIPK1
A_23_P151614	5.97E-03	2.84E-03	-3.00	PSME1

A_32_P178800	8.83E-06	5.17E-07	-5.15	ITGA2
A_24_P262355	1.62E-04	3.81E-05	2.30	PHB
A_24_P259490	1.45E-03	5.50E-04	1.94	ARF1
A_24_P91310	2.28E-05	2.55E-06	2.27	PSMC4
A_24_P823684	4.51E-05	7.12E-06	2.31	HSP90AB1
A_23_P433071	3.88E-06	1.08E-07	2.03	BECN1
A_23_P80398	1.13E-03	4.10E-04	2.01	NCAPH2
A_23_P300150	1.24E-03	4.57E-04	1.88	NFATC1
A_24_P289726	5.90E-04	1.90E-04	2.62	PSMD3
A_32_P351968	8.98E-03	4.52E-03	2.69	HLA-DMB
A_23_P67708	8.75E-03	4.39E-03	1.81	TCF3
A_23_P81399	7.60E-06	3.51E-07	2.94	**SQSTM1**
A_24_P944827	1.81E-02	1.01E-02	1.53	ATG7
A_32_P86289	2.16E-02	1.23E-02	-3.27	CYP19A1
A_23_P116371	4.99E-02	3.22E-02	-5.29	MS4A1
A_24_P132518	2.05E-05	2.16E-06	1.83	IKBKB
A_23_P153311	2.03E-03	8.14E-04	1.74	TYK2
A_23_P38346	8.68E-06	4.99E-07	1.98	DHX58
A_23_P31921	4.35E-03	1.98E-03	2.69	ASS1
A_23_P42282	3.07E-05	4.08E-06	2.77	C4B
A_24_P351852	4.49E-03	2.05E-03	-1.69	LAT2
A_23_P201086	2.14E-03	8.67E-04	3.31	ARF1
A_23_P142380	6.68E-07	9.44E-10	1.79	AKAP8
A_23_P107565	2.37E-04	6.09E-05	3.73	AQP4
A_23_P12168	1.26E-06	9.20E-09	2.88	PSMD4
A_23_P18901	5.14E-04	1.63E-04	1.78	ATG12
A_23_P206960	7.26E-05	1.36E-05	1.66	SEC14L1
A_23_P257365	5.55E-03	2.62E-03	-3.12	GFI1
A_23_P115200	9.67E-04	3.42E-04	-4.34	FCRL4
A_23_P79911	1.23E-05	9.29E-07	2.14	PSMF1
A_23_P58443	1.88E-04	4.58E-05	2.09	ANKHD1-EIF4EBP3
A_23_P2492	9.50E-05	1.98E-05	2.59	C1S
A_23_P61149	2.19E-03	8.92E-04	1.73	INPP5D
A_23_P18903	6.47E-04	2.12E-04	1.91	HAVCR2
A_23_P137366	2.67E-04	7.22E-05	3.82	C1QB
A_23_P75889	2.43E-05	2.84E-06	2.11	PSMD13
A_23_P55706	3.40E-06	7.70E-08	1.82	RELB
A_32_P228775	4.99E-02	3.23E-02	2.10	PPP4R2
A_23_P122116	1.44E-02	7.79E-03	2.17	DDX41
A_23_P70843	2.31E-05	2.63E-06	1.85	BPGM
A_23_P25354	8.30E-03	4.12E-03	1.84	P2RX7
A_23_P7582	4.25E-05	6.57E-06	-1.59	TCF7
A_23_P503115	3.52E-04	1.01E-04	1.91	BCR
A_23_P136325	6.22E-03	2.98E-03	1.62	WIPF2
A_32_P94798	3.35E-05	4.75E-06	2.82	ANXA2
A_23_P332413	6.00E-06	2.21E-07	-5.21	SLFN13
A_23_P334892	4.21E-03	1.91E-03	-2.38	TMEM102
A_32_P63182	8.68E-06	4.99E-07	2.03	PDIA3
A_23_P73429	1.49E-05	1.31E-06	2.13	HCLS1
A_23_P217269	1.58E-03	6.09E-04	2.09	VSIG4

A_23_P87664	1.15E-02	5.95E-03	1.61	SART3
A_24_P412156	6.15E-05	1.10E-05	-4.66	CXCL12
A_23_P155624	7.30E-04	2.45E-04	2.48	AP2M1
A_32_P67533	6.00E-06	2.15E-07	1.40	L3MBTL3
A_23_P45025	7.30E-06	3.19E-07	2.15	MAPK10
A_23_P39116	4.13E-04	1.23E-04	2.38	LIG1
A_23_P329152	7.56E-03	3.71E-03	2.14	ILF3
A_24_P265523	2.09E-02	1.19E-02	-2.68	CR1
A_23_P94422	3.29E-05	4.58E-06	-3.71	MELK
A_23_P71268	1.55E-03	5.97E-04	2.25	AZGP1
A_23_P70384	5.12E-03	2.38E-03	1.97	RNF8
A_24_P184031	8.21E-06	4.26E-07	2.08	PHPT1
A_23_P95917	2.48E-06	4.06E-08	2.94	HLA-C
A_24_P44514	1.96E-06	2.48E-08	2.14	CIB1
A_23_P359091	5.39E-03	2.53E-03	1.73	SMAD3
A_23_P119196	9.27E-05	1.92E-05	2.14	KLF2
A_24_P216087	1.62E-03	6.25E-04	2.21	PAF1
A_23_P88404	2.58E-03	1.09E-03	2.52	TGFB3
A_23_P59005	1.64E-06	1.41E-08	2.05	TAP1
A_24_P329635	2.86E-04	7.89E-05	1.89	TSC1
A_23_P153745	9.62E-03	4.87E-03	2.12	IFI30
A_23_P52761	6.58E-03	3.18E-03	-2.65	MMP7
A_23_P375372	1.70E-04	4.05E-05	-5.31	FGA
A_23_P101407	2.65E-03	1.12E-03	2.74	C3
A_23_P60180	1.49E-05	1.30E-06	1.67	ABL1
A_24_P365365	1.22E-05	8.92E-07	1.96	TCF3
A_23_P210708	2.30E-05	2.60E-06	2.13	SIRPA
A_24_P337592	9.84E-03	5.01E-03	1.61	BTN2A2
A_24_P417984	8.69E-05	1.75E-05	2.30	PPP1R14B
A_32_P148058	4.76E-03	2.20E-03	-1.81	RUNX2
A_24_P50950	2.67E-05	3.31E-06	-4.17	CCDC88B
A_24_P206343	2.13E-02	1.21E-02	-1.83	MYO1G
A_23_P134426	8.86E-04	3.06E-04	3.08	GPNMB
A_23_P345575	3.03E-05	4.01E-06	1.97	**FOXO3**
A_23_P94501	8.45E-05	1.68E-05	2.40	ANXA1
A_23_P206819	1.81E-04	4.37E-05	-4.59	HSD3B7
A_23_P61371	3.40E-05	4.86E-06	1.70	TMEM173
A_23_P375494	4.78E-04	1.49E-04	2.63	CEBPA
A_24_P403244	9.64E-04	3.41E-04	4.52	PILRB
A_23_P45248	3.48E-02	2.12E-02	-4.11	SH2D1A
A_24_P393151	2.73E-02	1.60E-02	-3.63	LILRA1
A_23_P105957	1.21E-04	2.69E-05	2.13	ACTN1
A_23_P321703	1.71E-04	4.08E-05	1.57	BCL2A1
A_23_P85862	7.39E-03	3.61E-03	1.69	GON4L
A_23_P152181	5.09E-03	2.37E-03	1.72	POLR3E
A_24_P120934	2.66E-04	7.15E-05	2.09	GADD45G
A_24_P265506	1.35E-03	5.01E-04	-2.27	NTRK1
A_23_P115460	1.01E-06	4.08E-09	1.75	RPL22
A_32_P9700	2.37E-06	3.82E-08	1.94	PSMC1
A_23_P171249	1.67E-06	1.69E-08	1.98	IGBP1

A_23_P17560	6.07E-03	2.90E-03	3.32	KIF3B
A_23_P104804	2.89E-05	3.75E-06	2.69	ZBTB16
A_24_P346277	7.30E-06	3.15E-07	1.93	SRF
A_23_P86884	7.93E-03	3.92E-03	2.34	SART1
A_32_P201496	2.30E-05	2.59E-06	1.98	POLR3H
A_24_P8892	4.59E-06	1.46E-07	1.75	HDAC4
A_23_P119562	1.15E-02	5.97E-03	2.46	CFD
A_23_P151610	1.26E-06	7.59E-09	2.42	PSME1
A_24_P153416	1.56E-03	5.99E-04	-2.60	KIR2DS1
A_23_P47879	1.30E-05	1.03E-06	2.37	STAT6
A_23_P250251	2.34E-02	1.34E-02	-2.97	IFNA2
A_24_P128205	2.10E-05	2.23E-06	2.00	PSMD1
A_24_P394420	3.28E-02	1.97E-02	-5.10	SFXN1
A_23_P77971	3.71E-04	1.07E-04	-3.51	ITGA2B
A_23_P348831	2.69E-03	1.14E-03	-3.65	IL3
A_23_P155765	7.98E-06	3.93E-07	2.33	HMGB2
A_23_P142631	1.60E-06	1.33E-08	1.92	FKBP1B
A_23_P358944	3.60E-05	5.21E-06	1.69	PML
A_23_P256425	3.40E-02	2.06E-02	-2.21	ADAMDEC1
A_24_P107695	1.96E-04	4.83E-05	2.66	ACTN1
A_24_P402222	2.90E-02	1.72E-02	2.53	HLA-DRB3
A_24_P639441	2.05E-04	5.10E-05	1.95	CD59
A_24_P24343	2.54E-04	6.73E-05	-6.48	SIPA1L3
A_24_P269853	3.27E-02	1.97E-02	2.00	ACTR1A
A_24_P209772	5.99E-06	2.11E-07	2.45	DCTN3
A_24_P363679	1.99E-02	1.12E-02	2.51	ATP6V1H
A_24_P59667	2.22E-05	2.44E-06	1.66	JAK3
A_23_P127676	8.07E-06	4.01E-07	2.10	CTR9
A_23_P331598	1.50E-02	8.12E-03	-1.63	IPO7
A_23_P407074	4.36E-03	1.98E-03	2.07	DNM2
A_23_P21853	1.06E-04	2.27E-05	2.10	AP2A2
A_23_P500271	1.10E-02	5.70E-03	1.66	IRF5
A_23_P73593	2.30E-02	1.32E-02	1.80	MSN
A_24_P262407	9.14E-04	3.19E-04	2.40	THRA
A_24_P113295	5.48E-06	1.83E-07	2.46	AP2S1
A_24_P139943	9.09E-04	3.17E-04	1.77	HS1BP3
A_23_P2920	2.12E-04	5.32E-05	11.17	SERPINA3
A_23_P39074	5.31E-05	9.03E-06	1.60	RRAS
A_23_P19590	3.53E-06	8.93E-08	2.55	EZR
A_24_P363711	2.43E-04	6.30E-05	-4.49	DEFA6
A_23_P24031	3.47E-02	2.11E-02	-2.81	TLX1
A_23_P206760	6.92E-04	2.30E-04	3.10	HP
A_24_P97104	1.44E-03	5.44E-04	-2.55	DPP4
A_23_P158024	6.40E-06	2.48E-07	2.33	DCTN3
A_23_P75811	2.53E-04	6.68E-05	2.55	SLC3A2
A_23_P117494	1.01E-06	4.00E-09	2.16	MTHFD1
A_23_P128728	1.67E-03	6.51E-04	1.93	ARG2
A_23_P30848	4.12E-03	1.86E-03	3.04	HLA-E
A_23_P20704	1.94E-02	1.09E-02	-2.78	IFNA10
A_24_P111342	1.26E-05	9.77E-07	1.49	CASP9

A_23_P171255	1.74E-04	4.18E-05	1.78	IGBP1
A_24_P367900	1.17E-04	2.59E-05	-2.74	ANGPT2
A_23_P31006	3.40E-03	1.49E-03	3.01	HLA-DRB5
A_23_P257638	3.13E-06	6.38E-08	2.06	CIB1
A_24_P353794	4.32E-04	1.31E-04	2.05	GALNT2
A_23_P258769	1.76E-04	4.24E-05	2.63	HLA-DPB1
A_23_P128744	2.73E-02	1.60E-02	-2.15	BDKRB1
A_23_P87545	2.77E-05	3.49E-06	2.59	IFITM3
A_24_P12397	3.94E-02	2.45E-02	2.20	TREM2
A_23_P33752	7.09E-06	2.98E-07	2.12	SLC25A6
A_24_P278367	3.18E-06	6.65E-08	3.15	ACTR1A
A_24_P366652	2.15E-05	2.31E-06	-4.91	ITGAD
A_23_P119526	2.70E-02	1.58E-02	2.39	AP3D1
A_23_P7144	5.04E-04	1.59E-04	2.02	CXCL1
A_23_P70670	4.59E-06	1.48E-07	2.26	CD83
A_24_P221414	2.66E-05	3.30E-06	2.27	DYNC1I1
A_23_P4662	5.75E-03	2.73E-03	1.71	BCL3
A_24_P682285	6.08E-05	1.08E-05	3.53	HSPA1A
A_32_P137035	1.91E-03	7.63E-04	1.66	TRIM27
A_23_P203332	8.21E-06	4.22E-07	1.59	FAM111A
A_24_P226554	3.53E-06	8.86E-08	2.64	ACTB
A_32_P356316	1.12E-04	2.44E-05	1.75	HLA-DOA
A_32_P29759	1.26E-06	9.57E-09	2.60	IK
A_23_P433865	2.07E-03	8.33E-04	-4.47	C2
A_23_P54540	8.16E-03	4.04E-03	1.89	EIF2AK4
A_23_P12282	1.00E-03	3.58E-04	1.70	DYRK3
A_23_P73589	3.53E-06	8.63E-08	2.35	MSN
A_24_P165965	4.24E-04	1.27E-04	1.51	TUSC2
A_23_P119130	6.00E-06	2.16E-07	2.20	RPS19
A_32_P173481	3.40E-06	7.96E-08	1.95	IK
A_23_P207600	6.10E-06	2.30E-07	2.17	PSMD11
A_23_P104607	2.48E-03	1.03E-03	3.07	PSMC3
A_23_P127088	4.04E-05	6.15E-06	2.20	CUEDC2
A_23_P211985	8.64E-03	4.33E-03	1.87	SNRK
A_24_P82200	1.24E-02	6.50E-03	1.59	MEIS2
A_23_P137035	3.22E-05	4.41E-06	1.89	PIR
A_24_P472055	4.59E-03	2.11E-03	-2.14	RNF125
A_24_P943792	4.78E-04	1.49E-04	2.11	CD47
A_23_P259621	1.41E-03	5.31E-04	1.83	LAT2
A_24_P370472	1.53E-02	8.35E-03	2.68	HLA-DRB4
A_23_P166371	8.92E-03	4.48E-03	-1.99	VPREB3
A_23_P306507	2.68E-02	1.57E-02	1.84	KRAS
A_23_P138194	6.66E-04	2.20E-04	2.04	NCF2
A_23_P71624	1.97E-02	1.11E-02	-2.18	PAX5
A_23_P130040	7.08E-05	1.32E-05	1.88	PHB
A_23_P383377	2.24E-05	2.48E-06	-4.02	KLF1
A_23_P22096	1.34E-06	1.08E-08	2.16	PTK2
A_23_P348636	1.25E-02	6.55E-03	1.91	FOXJ1
A_23_P214627	8.72E-04	3.00E-04	2.23	AIF1
A_23_P125107	8.89E-06	5.35E-07	3.32	HLA-B

A_24_P388528	1.02E-04	2.15E-05	1.85	ST6GAL1
A_23_P381979	9.50E-05	1.98E-05	2.00	OGT
A_23_P37497	1.96E-04	4.81E-05	1.77	MYO1E
A_32_P156963	9.21E-06	5.73E-07	2.33	ACTG1
A_23_P217688	1.81E-05	1.80E-06	1.81	TSC22D3
A_23_P38959	8.64E-03	4.33E-03	1.66	VAV1
A_23_P48088	3.02E-04	8.42E-05	-1.64	CD27
A_24_P74753	2.23E-03	9.11E-04	2.40	ATP6AP1
A_24_P244356	9.26E-06	5.92E-07	1.81	NLRX1
A_23_P500676	2.10E-02	1.20E-02	-3.03	IL5RA
A_24_P88696	8.27E-03	4.10E-03	2.85	**SCG2**
A_24_P86993	1.74E-04	4.19E-05	2.19	JAM3
A_23_P133245	7.74E-06	3.70E-07	1.99	IK
A_24_P166443	1.49E-05	1.30E-06	2.99	HLA-DPB1
A_23_P361773	2.65E-02	1.54E-02	1.69	CCND3
A_32_P234459	2.53E-04	6.70E-05	4.30	HLA-H
A_24_P113674	7.30E-06	3.24E-07	2.57	HLA-B
A_24_P373174	3.68E-02	2.27E-02	-2.58	RAB27A
A_24_P226108	2.11E-04	5.30E-05	-6.01	RBM47
A_23_P39465	1.12E-04	2.43E-05	2.00	BST2
A_23_P200767	3.41E-04	9.71E-05	1.68	TROVE2
A_24_P224727	8.89E-05	1.81E-05	2.14	CEBPA
A_23_P63798	5.98E-06	2.09E-07	2.30	KLF6
A_24_P216165	2.09E-04	5.23E-05	-2.49	CEBPA
A_23_P111662	2.10E-05	2.23E-06	-5.16	ABCB5
A_24_P166042	3.79E-04	1.10E-04	1.99	IMPDH2
A_32_P186921	3.29E-05	4.59E-06	-1.42	ZNF616
A_23_P64525	7.95E-05	1.54E-05	-4.00	RAG2
A_23_P337242	1.26E-06	8.32E-09	-1.75	TGFBR2
A_24_P94916	1.78E-02	9.88E-03	1.88	LST1
A_23_P32404	1.79E-06	1.93E-08	1.87	ISG20
A_23_P31323	2.70E-06	4.61E-08	2.60	ACTB
A_24_P141688	1.10E-04	2.38E-05	1.59	PCBP2
A_23_P413641	6.00E-06	2.20E-07	1.78	PREX1
A_23_P163347	3.98E-04	1.17E-04	1.57	PIAS1
A_23_P390518	4.20E-04	1.26E-04	-2.06	TNFRSF11A
A_23_P312132	4.63E-02	2.94E-02	1.77	ITGAX
A_23_P158239	2.34E-02	1.35E-02	1.75	SHMT2
A_23_P112798	3.05E-03	1.31E-03	2.41	CRIP2
A_23_P501538	7.30E-06	3.22E-07	-2.04	HOXA3
A_23_P146644	5.59E-05	9.67E-06	3.00	ANXA2
A_23_P28279	2.25E-04	5.75E-05	2.26	ACTR1B
A_23_P368805	1.22E-05	9.06E-07	-1.79	HHLA2
A_32_P9543	1.21E-02	6.30E-03	-1.75	APOBEC3A

In bold are highlighted candidate genes already known to contribute to ALS susceptibility.
*Gene expression fold-changes are given on a linear scale.

Supplementary Table 4. Neuroinflammatory genes differentially expressed in SALS2.

Probe ID	p Value (Corr)	p Value	Fold Change (SALS2/CTRL[+])[*]	GENE_SYMBOL
A_23_P126540	3.37E-03	1.47E-03	2.20	ADORA3
A_23_P82886	6.84E-03	3.33E-03	1.58	DEFA6
A_23_P426292	1.03E-04	2.20E-05	-1.82	**MAPK14**
A_23_P48936	2.22E-05	2.44E-06	-1.70	SMAD3
A_23_P119478	3.66E-05	5.33E-06	-1.68	EBI3
A_24_P202522	1.56E-05	1.41E-06	-2.78	AQP4
A_23_P169629	3.49E-05	5.00E-06	-2.04	SHMT2
A_24_P129277	3.04E-03	1.30E-03	1.94	NOD1
A_23_P87973	1.53E-05	1.38E-06	-3.79	TRIM13
A_23_P7325	2.23E-05	2.45E-06	2.03	BST1
A_24_P295999	1.49E-04	3.45E-05	-3.87	CD4
A_32_P186474	2.55E-03	1.07E-03	-2.46	RACGAP1
A_24_P337700	1.10E-02	5.64E-03	-2.45	VNN1
A_32_P154830	8.21E-06	4.24E-07	-1.69	OSTM1
A_23_P89249	7.67E-04	2.59E-04	1.62	ERBB2
A_24_P139191	2.31E-05	2.62E-06	-3.19	ITCH
A_23_P54373	7.35E-05	1.39E-05	-2.96	RAB27A
A_24_P83615	4.47E-03	2.04E-03	-2.73	NLRP1
A_23_P308603	3.22E-05	4.41E-06	-4.30	SRC
A_23_P309739	2.75E-02	1.61E-02	-1.56	ESR1
A_24_P308096	1.63E-04	3.86E-05	-2.76	JAK3
A_23_P138760	2.44E-03	1.01E-03	1.57	CLCF1
A_23_P2990	3.94E-03	1.76E-03	3.17	CEBPE
A_32_P61757	2.49E-05	3.00E-06	-4.24	PTPN11
A_32_P530933	1.08E-02	5.53E-03	-3.37	PYGO1
A_23_P35456	1.11E-04	2.41E-05	-3.13	SH3PXD2A
A_23_P31945	1.42E-05	1.18E-06	-3.07	IL33
A_23_P84705	1.27E-05	9.92E-07	9.01	TNFRSF13B
A_23_P68740	1.71E-03	6.72E-04	-1.75	AIRE
A_23_P68155	7.93E-04	2.70E-04	-1.60	IFIH1
A_23_P208706	8.45E-05	1.68E-05	-2.51	**BAX**
A_23_P99985	2.04E-06	2.85E-08	-2.28	HMGB1
A_23_P35916	7.95E-05	1.55E-05	3.99	ATM
A_23_P135248	5.08E-04	1.61E-04	1.81	CCL27
A_23_P168836	7.40E-03	3.62E-03	2.30	PTK2B
A_23_P70688	6.08E-05	1.08E-05	-2.66	LY86
A_23_P167096	2.18E-04	5.51E-05	-2.00	VEGFC
A_24_P354800	2.43E-05	2.84E-06	-3.57	HLA-DOA
A_23_P343799	4.00E-04	1.18E-04	3.56	SOX6
A_23_P29885	2.56E-05	3.12E-06	-2.63	MAEA
A_23_P23303	6.68E-07	5.52E-10	4.12	EXO1
A_24_P134074	5.90E-05	1.03E-05	-2.88	RPS19
A_23_P1691	4.39E-06	1.36E-07	2.79	MMP1
A_23_P50907	1.61E-04	3.80E-05	-2.37	ITGAV
A_24_P142118	2.99E-03	1.28E-03	-1.81	THBS1
A_23_P70095	1.39E-02	7.41E-03	2.10	CD74
A_23_P101992	3.31E-02	1.99E-02	2.98	MARCO

A_23_P40174	4.13E-05	6.31E-06	-3.17	**MMP9**
A_23_P134454	8.55E-05	1.72E-05	-2.65	CAV1
A_23_P29939	1.94E-05	1.98E-06	-3.64	SNCA
A_23_P94879	6.68E-07	1.99E-10	3.82	F2
A_24_P151464	1.60E-05	1.48E-06	-4.52	**SOD1**
A_23_P62953	1.25E-02	6.59E-03	5.61	PBX1
A_23_P162874	1.59E-03	6.16E-04	-3.86	HSP90AA1
A_23_P70020	8.53E-06	4.75E-07	-2.80	PFDN1
A_24_P898583	1.64E-05	1.54E-06	-1.89	TRIM26
A_23_P376096	1.67E-02	9.20E-03	-1.70	TICAM1
A_23_P115792	9.20E-04	3.21E-04	-2.47	PLEKHA1
A_23_P205686	6.64E-06	2.63E-07	-2.28	**PSEN1**
A_23_P203191	5.23E-05	8.83E-06	1.81	APOA1
A_24_P343695	3.89E-06	1.13E-07	2.19	RET
A_23_P107073	1.49E-05	1.31E-06	-2.42	RPA1
A_23_P212617	4.58E-05	7.29E-06	-2.04	TFRC
A_24_P227927	4.51E-04	1.39E-04	2.05	IL21R
A_32_P181077	1.56E-04	3.65E-05	-2.32	DOCK8
A_23_P63668	7.62E-06	3.58E-07	-1.75	IFIT5
A_23_P140967	1.20E-03	4.40E-04	-3.13	MEFV
A_23_P111571	7.97E-05	1.55E-05	-7.00	HOXA3
A_24_P92683	2.91E-03	1.24E-03	-1.91	IGHA1
A_23_P213706	1.96E-06	2.39E-08	6.64	IL4
A_23_P207596	6.84E-04	2.27E-04	-3.35	NF1
A_23_P106024	4.36E-05	6.82E-06	-1.53	JAG2
A_23_P43049	1.18E-05	8.55E-07	-3.65	DCTN6
A_32_P115015	6.84E-05	1.26E-05	-2.23	CDC42
A_23_P7313	3.02E-04	8.43E-05	-2.80	SPP1
A_23_P6272	9.09E-05	1.87E-05	-2.15	ICOSLG
A_23_P103361	1.17E-02	6.08E-03	-1.66	LCK
A_24_P184535	8.52E-05	1.70E-05	-1.91	C12orf4
A_23_P117546	1.02E-02	5.22E-03	-1.66	SOS2
A_24_P943283	1.43E-03	5.37E-04	-2.34	DENND1B
A_24_P274270	1.26E-06	9.85E-09	-2.00	STAT1
A_23_P77847	8.52E-05	1.71E-05	-2.87	GRB2
A_23_P72096	3.33E-05	4.70E-06	2.08	IL1A
A_24_P291016	1.88E-03	7.48E-04	1.80	ABL1
A_32_P151102	3.39E-05	4.83E-06	2.10	MSN
A_23_P83277	1.02E-04	2.15E-05	-1.81	IL11RA
A_24_P158089	2.45E-05	2.89E-06	-2.46	SERPINE1
A_24_P93948	1.26E-05	9.74E-07	-2.71	KIF5A
A_24_P916496	3.48E-04	9.97E-05	-3.11	**PRKCA**
A_24_P100742	1.22E-04	2.73E-05	2.80	ADD1
A_24_P929754	5.12E-03	2.38E-03	-2.10	MKNK2
A_24_P852756	9.26E-04	3.24E-04	-3.29	HLA-DQA2
A_23_P215406	8.71E-06	5.05E-07	-3.88	RAC1
A_24_P260443	7.44E-06	3.38E-07	-2.49	THBS4
A_23_P57036	1.28E-03	4.73E-04	1.43	CD40
A_23_P10291	1.24E-04	2.78E-05	-1.91	CTSE
A_23_P153616	6.60E-05	1.20E-05	-2.36	MADCAM1

A_23_P394605	2.48E-02	1.44E-02	-1.72	SEC24A
A_24_P295010	4.04E-05	6.13E-06	-4.85	SERPINB9
A_32_P214943	9.76E-03	4.96E-03	2.27	PCBP2
A_23_P209712	4.49E-03	2.05E-03	-2.03	SP100
A_23_P171324	3.30E-05	4.61E-06	2.86	L1CAM
A_23_P373028	1.80E-03	7.10E-04	-1.82	CACNA1C
A_24_P56388	9.09E-05	1.87E-05	-2.34	HIF1A
A_23_P502312	2.15E-04	5.43E-05	-2.23	CD97
A_23_P37535	1.25E-04	2.81E-05	-3.39	RAB8B
A_24_P278008	5.30E-05	9.00E-06	-2.41	DCTN6
A_24_P932233	3.15E-04	8.86E-05	-2.39	TET2
A_23_P71037	4.07E-04	1.20E-04	-2.21	IL6
A_23_P126735	2.54E-04	6.71E-05	-2.48	IL10
A_23_P69491	3.53E-06	9.01E-08	-3.34	RHOA
A_23_P253524	1.01E-04	2.12E-05	-2.37	CENPE
A_23_P170273	2.90E-04	8.00E-05	-1.83	SEC31A
A_24_P254079	6.81E-03	3.31E-03	2.96	ICAM5
A_24_P209765	3.53E-06	8.95E-08	-2.83	DCTN3
A_23_P143242	1.46E-03	5.55E-04	-1.58	CEBPB
A_23_P257895	1.28E-02	6.78E-03	1.88	MAPK1
A_23_P58293	1.01E-06	4.34E-09	-3.49	UBE2D3
A_24_P246196	3.40E-03	1.48E-03	2.18	CLEC4M
A_24_P156769	2.87E-05	3.69E-06	-5.63	MPL
A_23_P154306	1.96E-06	2.36E-08	-3.67	TANK
A_23_P254091	3.28E-06	7.28E-08	-2.14	KIF2A
A_23_P167537	3.41E-04	9.71E-05	-2.79	CPLX2
A_23_P48570	5.59E-03	2.64E-03	-1.73	DHRS2
A_23_P85250	7.92E-03	3.90E-03	-1.53	CD24
A_23_P81912	5.03E-06	1.64E-07	-2.15	TUBB
A_23_P24433	2.86E-04	7.89E-05	-2.85	CTSF
A_23_P151294	1.01E-06	3.77E-09	1.94	IFNG
A_23_P26810	6.00E-06	2.20E-07	1.90	**TP53**
A_23_P79628	3.14E-05	4.22E-06	-4.02	PSME4
A_23_P2960	7.30E-06	3.17E-07	1.92	**AKT1**
A_23_P209269	2.30E-05	2.58E-06	-3.15	PPM1B
A_32_P223777	8.83E-06	5.17E-07	-2.09	IL6ST
A_23_P89410	6.77E-05	1.24E-05	-2.07	BECN1
A_23_P62920	1.26E-05	9.68E-07	-3.34	**KIFAP3**
A_23_P121064	4.78E-04	1.49E-04	-2.12	PTX3
A_23_P300033	2.78E-04	7.56E-05	-1.67	PDGFRA
A_23_P372255	5.34E-05	9.14E-06	-2.62	ITPKB
A_23_P144054	3.14E-05	4.21E-06	-2.88	PRKCD
A_23_P36397	1.67E-05	1.60E-06	-2.63	CYP27B1
A_23_P99540	3.48E-04	9.96E-05	-2.52	ZFP36L1
A_23_P55281	1.63E-03	6.30E-04	3.92	HOXB7
A_23_P106682	1.07E-04	2.31E-05	-2.08	EMP2
A_23_P149545	7.60E-03	3.74E-03	-2.10	HIST2H2BE
A_23_P91619	6.02E-05	1.06E-05	-3.98	MIF
A_23_P250462	2.67E-05	3.34E-06	-2.77	ATP6AP1
A_23_P52278	1.79E-05	1.77E-06	3.80	KIF11

A_23_P72050	2.22E-06	3.47E-08	-2.20	PTK2
A_23_P30736	3.76E-03	1.67E-03	-2.77	HLA-DOB
A_23_P252062	1.82E-03	7.19E-04	2.02	PPARG
A_24_P167473	1.98E-05	2.04E-06	-2.06	ARPC3
A_23_P12113	1.68E-03	6.56E-04	5.01	FLVCR1
A_23_P348894	2.61E-04	6.98E-05	-2.18	SEC23A
A_23_P407718	3.63E-02	2.23E-02	-1.62	TRIM59
A_23_P87216	3.30E-05	4.61E-06	-2.95	SLC3A2
A_23_P20970	3.18E-06	6.67E-08	-2.10	ATG12
A_23_P104689	1.46E-05	1.26E-06	-3.54	RELA
A_23_P77228	4.22E-05	6.47E-06	-1.54	CRTC3
A_23_P54206	3.06E-04	8.58E-05	3.21	TSHR
A_24_P38702	1.73E-02	9.54E-03	-2.12	NKX2-3
A_23_P10873	2.52E-04	6.62E-05	-3.24	TLR1
A_23_P210210	4.24E-04	1.28E-04	-2.62	EPAS1
A_23_P127438	3.03E-05	4.01E-06	-2.95	FAU
A_23_P81492	9.66E-06	6.26E-07	-2.78	RPS14
A_24_P337746	5.21E-05	8.77E-06	-1.69	RABGEF1
A_23_P216756	1.16E-03	4.23E-04	3.22	FCN2
A_23_P44768	3.95E-05	5.90E-06	-2.30	**TBK1**
A_23_P67466	1.04E-05	7.07E-07	-2.49	PSMD8
A_23_P95960	6.31E-05	1.14E-05	-1.97	POLR3D
A_23_P218770	1.56E-03	6.02E-04	-1.59	RAC2
A_23_P123343	8.34E-04	2.85E-04	-1.55	NUDCD1
A_24_P412976	2.79E-05	3.52E-06	-4.16	TMEM143
A_23_P140916	4.24E-06	1.30E-07	-2.04	GLG1
A_23_P103522	2.81E-03	1.20E-03	4.96	**SELL**
A_23_P377750	4.10E-04	1.22E-04	4.17	FGF10
A_23_P396135	8.80E-03	4.42E-03	-2.07	NKX2-5
A_23_P305677	6.90E-06	2.80E-07	-1.98	UBE2D2
A_24_P76809	3.73E-03	1.66E-03	-1.76	PTPRO
A_23_P9255	3.62E-05	5.27E-06	-2.60	SYK
A_24_P388322	8.97E-03	4.51E-03	2.13	COLEC11
A_24_P71973	1.45E-04	3.32E-05	-2.12	**KDR**
A_23_P311616	1.72E-03	6.76E-04	2.35	JMJD6
A_23_P29994	8.12E-06	4.08E-07	-2.15	RBPJ
A_23_P112602	1.52E-05	1.37E-06	-1.91	TRIM62
A_23_P51231	1.81E-03	7.18E-04	4.30	RUNX3
A_23_P140301	2.22E-05	2.44E-06	-2.48	PSMA3
A_23_P139820	8.42E-05	1.67E-05	-2.86	SLC11A2
A_23_P162486	4.25E-05	6.56E-06	1.78	PTPN6
A_23_P206212	7.90E-07	1.70E-09	3.12	THBS1
A_23_P40470	3.70E-03	1.64E-03	4.23	HIST1H2BE
A_23_P369815	2.61E-04	7.02E-05	2.20	FASLG
A_24_P133253	3.11E-03	1.34E-03	-1.79	KITLG
A_23_P167250	1.46E-03	5.53E-04	2.30	IL21
A_23_P417891	1.33E-04	3.00E-05	-3.35	ARHGEF7
A_24_P365767	7.27E-05	1.38E-05	-2.94	CYBB
A_23_P329593	3.98E-03	1.79E-03	-2.00	SEC24A
A_23_P34788	9.14E-04	3.19E-04	-2.08	KIF2C

A_24_P918175	1.84E-04	4.45E-05	3.11	TBK1
A_24_P263793	1.93E-04	4.73E-05	1.59	FCN1
A_23_P99883	1.20E-04	2.65E-05	-3.04	PDIA3
A_23_P17837	3.40E-05	4.87E-06	5.45	APOL1
A_23_P42935	1.37E-02	7.30E-03	-1.45	BRAF
A_24_P934487	5.64E-03	2.67E-03	5.00	STXBP2
A_24_P305597	3.35E-05	4.77E-06	-1.72	ADRM1
A_23_P206177	1.97E-04	4.86E-05	-2.50	SNAP23
A_32_P120977	8.16E-06	4.15E-07	-1.68	PRELID1
A_24_P200023	2.83E-04	7.78E-05	-1.94	IL1R1
A_32_P4364	3.35E-05	4.75E-06	-3.10	BCAP31
A_23_P500244	4.00E-05	6.04E-06	-1.95	TRPV1
A_23_P502142	1.33E-05	1.07E-06	-2.28	FYN
A_23_P423457	7.78E-04	2.63E-04	-2.23	SERINC5
A_23_P5778	1.61E-03	6.23E-04	3.64	RAB17
A_23_P340728	6.72E-06	2.67E-07	-2.10	**PSEN1**
A_23_P204689	1.66E-05	1.59E-06	-2.35	CLEC2D
A_23_P387552	5.35E-04	1.70E-04	-2.65	RARG
A_24_P237265	4.78E-05	7.85E-06	-1.87	MAPK1
A_24_P418418	8.02E-05	1.56E-05	-2.92	RPS17
A_24_P236437	2.48E-05	2.96E-06	-3.36	DYNC1LI2
A_23_P26965	1.19E-04	2.62E-05	3.21	CCL13
A_23_P108785	1.44E-05	1.24E-06	-2.62	ACTR3
A_24_P917783	3.25E-02	1.96E-02	-2.01	RUNX1
A_32_P89837	1.07E-04	2.31E-05	-2.16	TRAF3
A_24_P933151	2.32E-06	3.68E-08	2.67	HOXB6
A_23_P101783	2.99E-03	1.28E-03	-1.96	IL12RB1
A_23_P218456	9.04E-06	5.52E-07	-2.93	ILF3
A_24_P927507	2.74E-03	1.17E-03	-2.35	OSBPL1A
A_23_P250564	8.40E-06	4.47E-07	-2.71	PRKCE
A_23_P301138	5.09E-05	8.46E-06	-3.96	IP6K2
A_23_P121253	1.57E-02	8.57E-03	-1.53	TNFSF10
A_32_P162187	2.25E-04	5.71E-05	-2.70	C2
A_23_P204208	2.33E-05	2.66E-06	5.05	KLRD1
A_23_P81805	1.95E-03	7.80E-04	-1.79	**VEGFA**
A_24_P400729	1.81E-04	4.37E-05	-1.85	PPP4R2
A_23_P13899	1.58E-05	1.45E-06	-3.20	GAPDH
A_23_P165968	1.40E-02	7.53E-03	4.31	SEMG1
A_23_P142455	1.24E-05	9.44E-07	-2.63	SIRT2
A_23_P256375	7.09E-06	2.97E-07	-2.13	STX4
A_23_P372910	2.34E-03	9.65E-04	-2.92	RORC
A_23_P404965	1.63E-05	1.52E-06	-3.92	GNL1
A_24_P145377	1.32E-04	2.97E-05	-5.35	HSP90AA1
A_23_P152655	9.26E-06	5.90E-07	-1.92	ICAM2
A_23_P134764	1.06E-02	5.43E-03	1.68	LY6D
A_23_P218442	2.06E-05	2.18E-06	-4.19	CEACAM6
A_23_P22671	1.26E-06	8.49E-09	-2.48	VAMP7
A_24_P401990	1.77E-03	6.96E-04	-2.31	ERCC2
A_23_P4885	1.65E-04	3.92E-05	1.54	AP2A1
A_23_P207981	6.84E-03	3.33E-03	4.34	SOCS6

A_23_P347198	1.86E-03	7.38E-04	-1.89	SP3
A_23_P168928	5.75E-04	1.85E-04	-2.10	CYP11B1
A_24_P322756	1.04E-02	5.33E-03	3.58	**TRPM2**
A_23_P258340	1.24E-05	9.43E-07	-4.60	PPIA
A_23_P75056	1.14E-05	8.08E-07	2.66	GATA3
A_23_P212844	2.68E-03	1.13E-03	5.93	TACC3
A_23_P253317	2.67E-04	7.20E-05	-1.96	GPR171
A_23_P4286	6.12E-04	1.98E-04	-2.26	XAF1
A_23_P30024	6.58E-06	2.57E-07	-2.71	NFKB1
A_23_P34888	1.47E-04	3.40E-05	-2.28	CHIA
A_32_P226149	5.89E-06	2.04E-07	-2.75	YWHAZ
A_24_P178011	3.18E-05	4.30E-06	-4.31	MYC
A_24_P930276	1.67E-03	6.51E-04	-3.34	NFE2L1
A_23_P315651	1.70E-04	4.07E-05	-4.77	CYBA
A_23_P26890	1.44E-03	5.44E-04	-2.50	MMP28
A_23_P126716	1.05E-04	2.25E-05	-4.90	ATPIF1
A_23_P11739	4.78E-05	7.84E-06	1.95	MIXL1
A_23_P133432	3.28E-06	7.31E-08	-4.35	SKP1
A_24_P8371	3.20E-05	4.33E-06	-2.68	SPNS2
A_23_P251945	2.15E-05	2.30E-06	-2.25	DCTN4
A_23_P79836	3.21E-06	6.91E-08	-3.32	SERINC3
A_24_P933908	3.91E-03	1.75E-03	2.95	GPNMB
A_23_P500282	4.45E-04	1.36E-04	-1.94	PACS1
A_23_P19543	2.43E-05	2.85E-06	-2.63	SRPK1
A_23_P136347	2.57E-03	1.08E-03	-2.99	EPS8
A_32_P11499	8.78E-07	2.12E-09	-2.61	UBE2N
A_23_P502464	7.60E-06	3.51E-07	-2.27	NOS2
A_23_P414855	3.23E-03	1.40E-03	1.80	PURB
A_24_P32935	7.47E-03	3.66E-03	-1.92	FOLR2
A_32_P68459	3.31E-05	4.64E-06	-5.00	PPIA
A_32_P517749	6.84E-03	3.33E-03	-1.86	RPS6KA3
A_24_P50245	2.36E-05	2.71E-06	-3.31	HLA-DMA
A_23_P146943	1.92E-04	4.69E-05	-4.56	ATP1B1
A_23_P126278	1.22E-02	6.39E-03	2.46	CHIT1
A_32_P175934	9.35E-05	1.94E-05	10.02	CD48
A_23_P113716	7.07E-06	2.91E-07	-2.38	HLA-C
A_24_P150361	1.66E-06	1.51E-08	-3.09	HSP90B1
A_24_P393711	1.82E-03	7.22E-04	4.24	ABL1
A_23_P201238	1.43E-04	3.25E-05	-2.24	SNX27
A_23_P145301	8.96E-04	3.12E-04	3.31	NFKBIL1
A_23_P11372	8.02E-05	1.57E-05	-2.51	HPRT1
A_23_P102471	9.29E-06	5.97E-07	-3.31	MSH2
A_23_P55828	2.45E-05	2.88E-06	2.16	CCL25
A_24_P389218	6.39E-03	3.08E-03	3.43	ARHGEF5
A_23_P204702	9.94E-07	3.21E-09	-3.43	TMBIM6
A_23_P32463	2.88E-05	3.71E-06	-2.23	EXOSC4
A_23_P342009	2.83E-04	7.77E-05	-2.28	**HFE**
A_23_P210176	5.04E-05	8.35E-06	-2.23	ITGA6
A_23_P158481	1.53E-04	3.58E-05	3.21	CYP2C19
A_23_P376060	2.73E-05	3.44E-06	-3.48	IKZF3

A_23_P61748	8.19E-05	1.61E-05	-2.45	COL4A3BP
A_23_P87036	2.57E-06	4.28E-08	2.82	APOA4
A_23_P71774	1.33E-02	7.03E-03	2.12	IFNB1
A_24_P344251	3.49E-04	9.99E-05	6.06	ITGAX
A_23_P37441	1.14E-05	8.06E-07	-4.39	B2M
A_23_P114783	5.52E-05	9.49E-06	-2.60	**PARP1**
A_23_P69368	7.21E-04	2.41E-04	2.89	TF
A_24_P300777	4.53E-05	7.18E-06	-1.85	ADAM8
A_23_P214444	1.68E-05	1.63E-06	-4.37	TAP2
A_32_P209960	3.36E-03	1.46E-03	-4.27	CIITA
A_24_P141481	1.11E-05	7.70E-07	-2.21	CD59
A_24_P311926	9.26E-06	5.92E-07	-2.60	HLA-G
A_32_P140656	3.39E-03	1.48E-03	-3.01	IL6ST
A_23_P109143	6.08E-05	1.08E-05	-3.42	PRNP
A_23_P105264	9.39E-03	4.75E-03	-3.03	ETV6
A_24_P418717	7.58E-05	1.45E-05	3.75	BMPR1A
A_23_P411296	1.29E-05	1.01E-06	-1.86	CEBPB
A_23_P11995	3.70E-06	9.54E-08	-2.72	PRDX1
A_23_P306148	2.69E-04	7.28E-05	4.30	PML
A_23_P346309	8.59E-03	4.29E-03	-2.09	**BAX**
A_23_P34345	3.56E-03	1.57E-03	3.66	VCAM1
A_23_P130815	3.40E-03	1.49E-03	-1.99	KIR2DS2
A_23_P61945	1.00E-04	2.11E-05	-2.75	MITF
A_23_P139786	2.17E-03	8.79E-04	-1.91	OASL
A_23_P254801	1.47E-03	5.57E-04	1.81	PLCG1
A_23_P109928	7.30E-06	3.26E-07	-3.41	PSMD6
A_23_P54991	2.98E-05	3.93E-06	-2.89	DYNLL2
A_23_P500034	6.28E-04	2.04E-04	1.94	GLI2
A_23_P86470	2.93E-04	8.11E-05	-1.69	CH25H
A_23_P397933	9.56E-04	3.37E-04	3.58	MASP1
A_24_P162483	2.20E-05	2.39E-06	7.02	ANK1
A_23_P164536	2.72E-04	7.39E-05	-2.48	PIK3C3
A_24_P261760	1.45E-04	3.31E-05	3.59	KLRG1
A_32_P177024	3.67E-05	5.37E-06	-2.78	SBDS
A_23_P85503	4.24E-04	1.28E-04	-2.53	PGLYRP4
A_24_P276853	1.29E-03	4.79E-04	2.16	GON4L
A_23_P47034	4.12E-03	1.86E-03	-1.57	HHEX
A_24_P397817	6.68E-07	3.63E-10	5.80	LEP
A_23_P168761	1.50E-05	1.32E-06	-2.74	PTPRZ1
A_24_P252078	1.81E-02	1.01E-02	1.76	BTN3A2
A_23_P91019	6.23E-06	2.39E-07	-2.12	PRKRA
A_23_P100872	7.92E-03	3.91E-03	2.58	HOXB3
A_23_P215900	2.28E-05	2.55E-06	-2.30	SCARA3
A_24_P45446	1.19E-02	6.20E-03	-1.56	GBP4
A_23_P14769	1.04E-02	5.31E-03	1.97	FES
A_23_P86635	2.19E-02	1.26E-02	1.97	DCLRE1C
A_23_P373031	8.09E-05	1.58E-05	-3.75	CACNA1C
A_23_P92410	9.05E-05	1.85E-05	-2.66	**CASP3**
A_23_P147185	1.02E-05	6.71E-07	-2.26	CANX
A_23_P38505	2.47E-04	6.46E-05	-2.60	CXCL16

A_24_P766716	2.55E-02	1.48E-02	1.88	CMKLR1
A_23_P52697	2.89E-05	3.76E-06	-2.02	CD248
A_24_P389517	9.84E-06	6.45E-07	-2.07	HNRNPK
A_24_P41850	3.69E-04	1.07E-04	3.48	MASP1
A_23_P314202	3.93E-03	1.75E-03	4.13	PAPD4
A_23_P207582	7.09E-06	2.99E-07	3.23	CCL16
A_23_P212179	2.24E-03	9.14E-04	3.72	HRH1
A_24_P403734	4.26E-05	6.60E-06	-2.36	ZNF385A
A_23_P38519	2.11E-02	1.20E-02	-1.93	ITGB3
A_23_P134419	3.62E-04	1.04E-04	1.74	ZP3
A_23_P6263	3.03E-04	8.48E-05	-2.24	MX2
A_24_P101201	1.70E-04	4.05E-05	-3.28	PDIA3
A_24_P103469	4.54E-03	2.08E-03	2.83	LST1
A_23_P149345	3.20E-05	4.35E-06	-2.95	PTPN22
A_23_P134147	1.45E-04	3.34E-05	-1.66	ZBTB24
A_23_P253345	1.16E-03	4.24E-04	-2.58	C8orf4
A_24_P367432	6.76E-04	2.24E-04	3.26	IGHV1-69
A_24_P245589	6.19E-05	1.12E-05	2.93	PLVAP
A_23_P392501	4.58E-05	7.28E-06	-2.95	TNRC6C
A_23_P17575	1.95E-05	1.99E-06	-3.21	AHCY
A_23_P11729	3.18E-04	8.97E-05	2.31	ZBTB7B
A_23_P160638	1.33E-02	7.05E-03	2.15	BGLAP
A_23_P204536	1.41E-05	1.16E-06	-2.10	SENP1
A_23_P326885	3.73E-02	2.30E-02	-2.12	SIX4
A_23_P213745	7.62E-05	1.46E-05	-2.48	CXCL14
A_23_P202905	9.55E-05	1.99E-05	4.12	TIRAP
A_23_P34093	1.09E-03	3.92E-04	2.34	G6PD
A_32_P25273	4.60E-05	7.37E-06	-3.28	HSPD1
A_23_P101141	5.33E-03	2.50E-03	-2.20	RNF125
A_23_P8913	1.68E-05	1.61E-06	-2.81	CA2
A_23_P67332	4.24E-05	6.51E-06	-1.58	NR1H2
A_23_P149281	3.89E-06	1.13E-07	3.38	EPHA2
A_32_P232865	6.76E-03	3.28E-03	3.78	BCR
A_23_P163787	2.73E-06	5.00E-08	2.55	**MMP2**
A_24_P402080	1.44E-05	1.21E-06	-2.02	MBP
A_23_P25644	6.68E-07	8.25E-10	-2.29	PCID2
A_23_P85693	1.02E-03	3.63E-04	2.64	GBP2
A_23_P250353	1.46E-05	1.25E-06	-2.57	HERC6
A_23_P99027	2.15E-05	2.29E-06	-2.80	PTPN11
A_23_P72737	2.20E-05	2.38E-06	-2.63	IFITM1
A_24_P249072	1.71E-05	1.68E-06	-1.60	BTN2A2
A_24_P92472	1.52E-05	1.36E-06	3.99	CFI
A_23_P79066	2.41E-04	6.23E-05	-2.30	SPPL2B
A_23_P367676	4.00E-06	1.18E-07	-2.72	SIN3A
A_23_P204654	1.47E-04	3.40E-05	11.61	KITLG
A_23_P2801	2.70E-02	1.58E-02	-1.73	ELF1
A_23_P102122	1.81E-05	1.81E-06	-2.79	ARPC2
A_23_P79161	3.88E-06	1.11E-07	-1.64	PRELID1
A_23_P165007	1.95E-02	1.10E-02	-2.57	RASGRP4
A_23_P116286	2.47E-05	2.93E-06	-2.81	AMPD3

A_24_P380919	5.10E-05	8.54E-06	-2.22	HNRNPK
A_23_P206585	6.08E-05	1.08E-05	-4.04	**PRKCB**
A_23_P158484	1.77E-05	1.75E-06	9.51	CYP2C19
A_23_P37191	3.13E-06	6.39E-08	-3.28	PSMB5
A_24_P209047	7.59E-04	2.56E-04	6.03	IL5
A_23_P7961	1.48E-03	5.66E-04	-3.21	PGC
A_24_P269779	1.54E-02	8.41E-03	3.00	CHST3
A_23_P164773	1.17E-05	8.46E-07	2.57	FCER2
A_23_P19592	2.50E-05	3.03E-06	-1.56	PGM3
A_24_P388252	5.24E-05	8.86E-06	-4.56	PPP3R1
A_23_P59657	6.00E-06	2.19E-07	-2.43	CUL1
A_23_P56069	2.73E-06	5.07E-08	-1.69	LSM14A
A_23_P214798	3.81E-06	1.01E-07	-3.48	SYNCRIP
A_23_P202334	1.66E-05	1.59E-06	-3.71	FGFR2
A_24_P57367	3.51E-02	2.14E-02	3.49	AHCY
A_23_P44112	8.96E-05	1.83E-05	-1.90	LAT
A_23_P155539	3.32E-04	9.40E-05	2.15	POU1F1
A_23_P104199	1.55E-04	3.62E-05	-1.70	ITGB1
A_23_P347070	1.26E-06	6.41E-09	-2.71	PAG1
A_23_P119778	7.30E-06	3.22E-07	-2.00	SLC39A10
A_23_P334083	1.65E-03	6.40E-04	3.75	TRIM14
A_23_P124108	1.34E-02	7.10E-03	-2.59	ITGAM
A_24_P110937	4.69E-03	2.16E-03	2.20	PCID2
A_24_P370682	2.78E-04	7.58E-05	-3.13	TRIM11
A_24_P98524	9.26E-06	5.90E-07	-4.02	PPP3CB
A_23_P210048	5.93E-05	1.04E-05	-4.28	HDAC4
A_23_P66682	4.00E-05	6.06E-06	-3.35	HOXB6
A_23_P163390	1.03E-04	2.19E-05	-2.21	CHRNA7
A_23_P97990	4.45E-04	1.36E-04	-3.10	HTRA1
A_23_P348194	4.11E-03	1.85E-03	2.49	PRKCE
A_24_P209571	1.41E-05	1.14E-06	-3.59	YWHAZ
A_23_P518	6.51E-04	2.13E-04	7.60	VTCN1
A_23_P402319	3.23E-03	1.40E-03	-2.13	GAPT
A_23_P17663	2.95E-05	3.86E-06	-2.66	MX1
A_23_P56195	6.68E-07	1.26E-09	-2.21	UBA52
A_32_P116203	1.39E-02	7.40E-03	1.99	NCF1
A_23_P124252	8.32E-06	4.35E-07	-2.08	CAMK1D
A_24_P373096	5.59E-05	9.70E-06	3.92	TGFB3
A_23_P381976	9.03E-03	4.55E-03	-2.34	OGT
A_23_P41999	3.40E-02	2.06E-02	-1.47	SMAP1
A_23_P250910	1.41E-05	1.17E-06	-2.02	UBQLN1
A_23_P142361	8.96E-04	3.11E-04	3.11	PIK3R2
A_23_P143143	7.51E-04	2.52E-04	-3.14	ID2
A_23_P203053	2.04E-03	8.20E-04	2.21	NCAM1
A_23_P79518	2.05E-02	1.16E-02	-1.64	IL1B
A_24_P108401	1.47E-03	5.60E-04	-3.72	KLK3
A_23_P356755	3.14E-05	4.20E-06	-1.91	CEBPG
A_23_P369919	2.73E-06	5.14E-08	-3.28	RAB4A
A_23_P30122	4.59E-06	1.48E-07	2.11	IL2
A_32_P11477	2.04E-06	2.80E-08	-3.59	FAU

A_24_P196534	7.72E-05	1.49E-05	-3.09	PKNOX1
A_24_P772061	1.30E-05	1.03E-06	-4.31	PPIA
A_23_P76529	5.26E-03	2.46E-03	-1.97	ITGB7
A_23_P45185	4.63E-03	2.13E-03	-1.90	FIGF
A_24_P381901	1.16E-02	6.00E-03	-2.14	CX3CL1
A_23_P169331	1.37E-02	7.28E-03	1.56	TRAF2
A_24_P62708	3.07E-04	8.61E-05	-3.75	PRKACB
A_23_P47709	7.61E-03	3.74E-03	-2.46	FOLR2
A_23_P156562	9.21E-06	5.75E-07	-2.54	CRIP3
A_23_P117298	3.49E-02	2.12E-02	1.59	F7
A_23_P122027	2.58E-05	3.17E-06	-4.56	KIF3A
A_23_P150609	6.45E-04	2.11E-04	-2.69	IGF2
A_23_P128503	6.05E-04	1.95E-04	2.94	IL26
A_23_P168692	2.44E-04	6.37E-05	2.70	DBNL
A_23_P251562	1.45E-04	3.34E-05	-2.96	TUSC2
A_24_P379379	3.18E-02	1.91E-02	2.84	CAPZA1
A_24_P222655	1.03E-02	5.29E-03	2.34	C1QA
A_23_P63584	1.38E-04	3.15E-05	-2.48	AHCTF1
A_32_P7721	2.04E-06	2.76E-08	-4.57	RORA
A_23_P129956	7.25E-06	3.10E-07	-2.69	DUSP3
A_24_P135856	4.93E-04	1.54E-04	2.66	RHD
A_23_P301133	1.22E-05	9.01E-07	-4.16	IP6K2
A_23_P119143	6.59E-05	1.20E-05	-2.10	ICAM5
A_23_P209740	2.30E-05	2.58E-06	-2.91	PSMD1
A_32_P92295	8.41E-06	4.59E-07	-2.85	RPL13A
A_23_P401106	3.81E-04	1.11E-04	-3.45	PDE2A
A_24_P239676	1.47E-03	5.59E-04	-2.13	HLA-DQB1
A_32_P151933	3.22E-03	1.39E-03	-1.52	FECH
A_23_P98282	8.34E-06	4.40E-07	-2.79	SPTBN2
A_23_P74290	8.86E-03	4.45E-03	3.00	GBP5
A_32_P217709	4.24E-06	1.29E-07	-2.22	RAC1
A_23_P75523	2.16E-05	2.32E-06	-2.88	CD59
A_24_P272061	1.18E-05	8.52E-07	-3.25	RPL13A
A_24_P42755	3.36E-02	2.03E-02	-1.73	FLT1
A_23_P134125	4.58E-05	7.31E-06	-1.95	MAP3K5
A_23_P120845	2.68E-05	3.35E-06	-4.08	XBP1
A_24_P397928	1.60E-05	1.48E-06	-2.13	CTSB
A_23_P408708	8.32E-06	4.36E-07	-3.06	BNIP3L
A_23_P73239	2.78E-04	7.57E-05	-2.05	NCKAP1
A_23_P23759	2.45E-03	1.02E-03	5.62	CFHR5
A_23_P88119	6.46E-05	1.17E-05	-3.67	HSPH1
A_23_P205567	9.12E-06	5.61E-07	-2.18	PRKCH
A_24_P356406	8.69E-05	1.75E-05	-2.99	PAFAH1B1
A_23_P15705	2.73E-06	5.05E-08	-2.52	PSMB6
A_23_P420873	3.15E-04	8.88E-05	-2.52	NR1D1
A_23_P134484	3.65E-03	1.62E-03	6.02	CALCR
A_24_P245298	2.82E-04	7.75E-05	-3.67	TNFSF12
A_23_P29225	1.67E-06	1.75E-08	-2.82	FBXO7
A_23_P38446	1.96E-06	2.25E-08	-2.31	TNFAIP1
A_23_P106741	2.02E-05	2.11E-06	-3.75	PSMD7

A_24_P940979	5.71E-03	2.71E-03	2.35	LGALS8
A_23_P68007	1.91E-05	1.93E-06	-2.38	ATP1B3
A_23_P121480	9.09E-05	1.87E-05	-3.40	CD200
A_23_P37375	1.17E-04	2.57E-05	-2.85	RPS6KA5
A_23_P51926	1.32E-02	7.01E-03	1.74	PTAFR
A_24_P304071	4.46E-02	2.83E-02	2.74	IFIT2
A_24_P329600	5.74E-06	1.94E-07	-3.68	UBQLN1
A_23_P92042	8.40E-06	4.56E-07	-4.83	ITPR1
A_23_P123112	1.71E-05	1.67E-06	2.21	ABCB4
A_32_P162183	1.06E-04	2.28E-05	-2.25	C2
A_24_P165998	1.70E-04	4.05E-05	4.39	GATA2
A_23_P209129	3.55E-04	1.02E-04	8.53	LAIR2
A_23_P254594	6.70E-03	3.25E-03	3.27	GNRH1
A_23_P107623	2.81E-04	7.70E-05	5.95	SERPINB12
A_23_P106002	1.98E-05	2.04E-06	-2.31	NFKBIA
A_23_P24211	2.08E-03	8.37E-04	2.73	MMP21
A_23_P154235	5.24E-04	1.67E-04	-2.19	NMI
A_23_P120254	5.53E-06	1.86E-07	-2.58	DUSP22
A_24_P12573	2.15E-02	1.23E-02	4.24	CCL26
A_24_P345679	5.17E-03	2.41E-03	-1.74	MLF1
A_24_P355944	3.90E-05	5.80E-06	-1.87	EFNB2
A_23_P69012	6.52E-05	1.18E-05	3.11	CCR8
A_23_P36364	7.57E-05	1.44E-05	-4.72	THY1
A_23_P305680	1.43E-03	5.41E-04	-3.15	IGF1R
A_24_P383480	7.19E-03	3.51E-03	4.18	PROS1
A_23_P88630	1.68E-05	1.62E-06	-2.64	BLM
A_23_P6099	2.94E-04	8.15E-05	-4.01	PLCB1
A_24_P408206	1.27E-03	4.71E-04	1.64	PRKACA
A_23_P213326	6.12E-05	1.09E-05	-1.99	ANKHD1
A_32_P203917	4.10E-03	1.85E-03	-2.81	C9
A_23_P407565	3.54E-04	1.02E-04	-3.21	CX3CR1
A_23_P366376	8.89E-06	5.32E-07	3.38	TDGF1
A_23_P321892	5.04E-03	2.34E-03	-3.09	HRG
A_23_P137139	2.82E-02	1.66E-02	2.26	BTK
A_23_P163178	1.56E-05	1.42E-06	-5.84	CALM1
A_24_P338121	8.69E-05	1.75E-05	4.89	UBQLN1
A_23_P102988	7.99E-04	2.72E-04	7.14	TXNRD2
A_24_P342178	2.56E-04	6.84E-05	-2.94	STAT5B
A_23_P26783	2.21E-04	5.60E-05	-4.09	PSMD3
A_23_P156928	6.32E-03	3.04E-03	1.69	GPLD1
A_24_P125335	2.90E-04	8.03E-05	-3.80	CCL13
A_24_P838748	5.34E-03	2.51E-03	-2.66	WIPF2
A_23_P147025	7.85E-05	1.52E-05	-3.80	RAB33A
A_24_P294851	3.18E-04	8.98E-05	7.10	TRIM38
A_23_P38346	8.68E-06	4.99E-07	-2.07	DHX58
A_24_P359856	4.38E-05	6.86E-06	2.73	HDAC4
A_23_P31921	4.35E-03	1.98E-03	3.27	ASS1
A_23_P72568	2.62E-05	3.24E-06	-3.77	SNX4
A_24_P84419	1.19E-02	6.20E-03	-2.34	VAV2
A_23_P104193	1.33E-05	1.07E-06	-2.66	ITGB1

A_23_P365614	7.55E-04	2.54E-04	-1.83	NOTCH4
A_23_P94412	4.80E-03	2.22E-03	3.19	PDCD1LG2
A_23_P47955	7.91E-04	2.68E-04	3.58	OAS3
A_24_P122050	1.21E-02	6.31E-03	2.34	PSMF1
A_24_P408704	1.17E-03	4.28E-04	6.69	DOCK2
A_24_P149314	1.96E-05	2.01E-06	7.54	ULBP2
A_23_P103334	1.46E-02	7.90E-03	-2.36	RC3H1
A_23_P317636	4.62E-03	2.13E-03	-2.38	ADD2
A_23_P407614	7.88E-04	2.67E-04	3.59	PYDC1
A_23_P152782	4.52E-04	1.39E-04	-1.99	IFI35
A_23_P154108	1.08E-06	4.91E-09	-3.23	DYNC1I2
A_24_P364335	1.26E-06	7.76E-09	-3.55	CLEC2D
A_24_P29277	8.40E-06	4.46E-07	-2.36	COL4A3BP
A_32_P165611	2.50E-05	3.02E-06	-2.86	KIF13B
A_23_P62652	2.45E-02	1.42E-02	2.73	SLAMF1
A_23_P1206	2.46E-05	2.92E-06	-3.17	RPS24
A_24_P313186	8.53E-06	4.70E-07	-4.31	CALM1
A_23_P85903	4.90E-02	3.16E-02	1.61	TLR5
A_23_P362659	1.60E-05	1.49E-06	-2.59	MYD88
A_24_P199774	2.48E-05	2.95E-06	4.08	TINAGL1
A_23_P117721	1.52E-05	1.35E-06	-2.65	RPS17
A_23_P142380	6.68E-07	9.44E-10	-1.81	AKAP8
A_24_P347488	1.13E-04	2.47E-05	-3.35	PSMC1
A_23_P34018	2.62E-03	1.11E-03	-2.72	RPL39
A_24_P51517	9.36E-04	3.29E-04	1.83	FGR
A_23_P77073	2.22E-03	9.08E-04	-1.93	SPPL2A
A_23_P23584	5.37E-05	9.20E-06	-2.43	CTNNBIP1
A_23_P143845	9.21E-06	5.71E-07	-2.93	TIPARP
A_24_P304760	2.69E-02	1.57E-02	2.45	SIN3A
A_23_P212034	5.10E-05	8.54E-06	-3.75	DLG1
A_23_P325155	2.36E-04	6.05E-05	2.86	CD200R1
A_23_P330262	7.93E-03	3.91E-03	2.11	PIP5K1C
A_24_P923591	2.05E-04	5.09E-05	6.09	PPIL2
A_23_P329112	1.07E-03	3.83E-04	1.75	JAK3
A_23_P77876	6.68E-07	1.08E-09	-2.48	PSMD12
A_24_P933319	1.02E-04	2.16E-05	-3.92	RAB3B
A_23_P29555	1.62E-04	3.82E-05	-2.83	SEC13
A_24_P100228	1.65E-02	9.06E-03	2.26	XBP1
A_23_P86216	1.67E-06	1.75E-08	-3.15	PSMA5
A_23_P251173	9.94E-07	2.70E-09	-2.95	EIF2AK1
A_23_P433588	2.93E-04	8.12E-05	-5.57	EFNB3
A_23_P259741	4.78E-04	1.49E-04	-4.31	SATB1
A_23_P134827	3.40E-06	7.90E-08	-2.81	ASH2L
A_23_P71867	2.04E-06	2.81E-08	-1.78	IL11RA
A_32_P181638	6.37E-04	2.08E-04	-3.93	BVES
A_23_P143987	1.30E-02	6.85E-03	-2.12	ATG7
A_24_P381494	2.81E-05	3.60E-06	-2.21	SLC11A2
A_23_P203215	1.81E-02	1.01E-02	-1.72	CXCR5
A_24_P108451	7.63E-05	1.46E-05	-3.93	GPI
A_24_P345993	8.40E-06	4.55E-07	-2.40	CANX

A_24_P56467	3.35E-05	4.75E-06	-2.64	GMPR2
A_24_P20607	3.64E-04	1.05E-04	2.91	CXCL11
A_23_P420209	1.82E-04	4.42E-05	3.92	GCNT3
A_24_P257539	1.57E-02	8.59E-03	-1.83	FBXW11
A_23_P217236	2.60E-05	3.21E-06	-1.84	HMGB3
A_23_P163079	2.87E-02	1.70E-02	4.61	GCH1
A_24_P944519	1.52E-02	8.26E-03	-2.47	PDE4D
A_23_P417282	1.89E-04	4.60E-05	-1.77	IGF1R
A_23_P164022	6.19E-05	1.12E-05	-3.54	MYO1C
A_24_P65722	1.10E-02	5.66E-03	2.26	LILRB4
A_23_P141405	1.46E-05	1.26E-06	-3.44	NME2
A_24_P932418	1.50E-04	3.50E-05	-2.76	AP2A2
A_24_P190894	9.27E-05	1.92E-05	-3.24	ARF1
A_24_P335620	2.05E-04	5.13E-05	-2.18	SLC7A5
A_23_P152804	6.11E-06	2.32E-07	-2.83	NME1
A_24_P64781	3.39E-02	2.05E-02	-4.30	KITLG
A_24_P941643	1.03E-05	6.88E-07	-6.55	PLCB1
A_23_P120942	6.04E-05	1.07E-05	-4.25	XRCC6
A_32_P104075	1.40E-05	1.13E-06	-2.13	PAK2
A_32_P17765	7.62E-04	2.57E-04	2.26	LGR4
A_23_P89798	1.81E-05	1.81E-06	-2.44	FECH
A_24_P124672	1.22E-05	9.03E-07	-3.97	DYNLL1
A_23_P90497	1.94E-04	4.75E-05	2.01	LILRA4
A_23_P374862	6.10E-03	2.91E-03	-1.79	CD55
A_23_P3562	3.28E-05	4.56E-06	-2.05	SLC7A6OS
A_23_P128993	1.12E-02	5.81E-03	1.76	GZMH
A_23_P200598	1.97E-04	4.85E-05	-1.88	MUL1
A_23_P132526	3.58E-02	2.20E-02	2.02	PIK3R4
A_23_P69109	6.94E-04	2.31E-04	-2.13	PLSCR1
A_23_P365719	2.43E-05	2.84E-06	-2.14	TAPBP
A_24_P28657	4.42E-04	1.35E-04	-1.67	AHCTF1
A_24_P722216	7.26E-05	1.37E-05	-3.31	DHTKD1
A_23_P121037	4.35E-04	1.32E-04	8.19	PODXL2
A_23_P61881	1.80E-04	4.34E-05	-2.46	ARIH2
A_32_P99275	5.52E-05	9.50E-06	-1.92	YTHDF2
A_23_P132159	2.56E-03	1.07E-03	-1.91	USP18
A_23_P46819	5.73E-05	9.96E-06	-2.68	BTRC
A_24_P310894	1.66E-05	1.57E-06	-3.58	CAPZA1
A_24_P166663	1.22E-05	8.92E-07	-2.99	CDK6
A_32_P220307	4.57E-04	1.41E-04	-3.86	RPL39
A_24_P285158	1.22E-05	9.10E-07	-4.24	FCRLB
A_23_P57497	3.68E-03	1.63E-03	2.79	MYH9
A_23_P151915	2.14E-02	1.22E-02	3.64	GCNT3
A_23_P214208	5.83E-05	1.02E-05	-2.64	CNR1
A_23_P143120	5.42E-03	2.55E-03	-2.21	ADAM17
A_23_P258418	1.05E-03	3.76E-04	4.17	TNIP2
A_23_P406438	2.80E-05	3.56E-06	-3.53	SRPK2
A_23_P6762	1.41E-05	1.16E-06	-2.57	JAGN1
A_32_P96807	2.50E-04	6.55E-05	-2.68	RC3H1
A_24_P404822	2.43E-05	2.82E-06	-2.41	APLN

A_23_P45871	8.84E-06	5.20E-07	-2.53	IFI44L
A_23_P500601	1.26E-06	6.64E-09	-2.10	TRIM4
A_24_P246173	8.68E-06	4.92E-07	-3.22	MYO9B
A_23_P51187	2.41E-04	6.22E-05	-3.27	**PRKCZ**
A_23_P74278	1.24E-05	9.55E-07	-2.25	PDE4B
A_32_P460973	5.83E-05	1.02E-05	-2.79	HLA-E
A_23_P37727	4.47E-04	1.37E-04	-3.72	CX3CL1
A_23_P360167	1.44E-05	1.23E-06	-2.39	DCTN2
A_32_P7502	8.95E-04	3.10E-04	2.49	CD96
A_23_P6464	3.52E-03	1.55E-03	-2.29	PLA2G6
A_23_P501732	3.03E-04	8.47E-05	4.72	FCN2
A_24_P284893	6.58E-06	2.58E-07	-1.94	PSMB2
A_23_P39735	1.10E-05	7.51E-07	3.11	IL18R1
A_23_P113245	8.68E-06	4.95E-07	-4.38	RBPJ
A_24_P143574	7.60E-04	2.56E-04	3.26	SIRPB1
A_24_P221424	2.37E-04	6.11E-05	1.69	ZNF784
A_23_P170820	1.56E-04	3.66E-05	-2.92	PUM1
A_23_P23438	1.75E-03	6.88E-04	-2.67	SEMA4A
A_23_P12392	1.36E-03	5.09E-04	6.09	PTPRC
A_23_P41344	2.58E-05	3.15E-06	4.66	EREG
A_23_P120125	5.30E-05	8.98E-06	-2.61	COLEC11
A_23_P200670	1.59E-03	6.14E-04	1.52	WDR78
A_24_P935033	8.27E-05	1.63E-05	9.48	IL6ST
A_23_P211299	6.66E-04	2.20E-04	-2.38	PKNOX1
A_23_P433753	4.68E-05	7.57E-06	-3.36	PRKAR1A
A_23_P136325	6.22E-03	2.98E-03	2.10	WIPF2
A_23_P138352	7.41E-03	3.62E-03	2.60	WNT2B
A_23_P166122	4.14E-04	1.24E-04	-2.92	POLR3F
A_23_P338479	7.67E-04	2.59E-04	-2.66	CD274
A_32_P66881	6.11E-05	1.09E-05	-1.75	TLR4
A_23_P24004	4.92E-05	8.12E-06	-2.72	IFIT2
A_24_P208081	2.25E-04	5.73E-05	2.58	PLA2G2F
A_23_P153390	2.73E-03	1.16E-03	-1.62	CLEC4G
A_23_P255126	1.53E-03	5.88E-04	2.95	GAB3
A_23_P67288	1.46E-05	1.27E-06	-2.34	CALR
A_23_P93360	6.25E-04	2.03E-04	-3.87	AGER
A_23_P32253	6.89E-06	2.78E-07	-1.87	NFIL3
A_23_P350574	2.19E-03	8.90E-04	-1.78	FCRLB
A_24_P105771	7.98E-06	3.95E-07	-2.12	ACTR3
A_24_P131752	8.16E-06	4.13E-07	-3.56	DLG1
A_23_P156117	2.73E-04	7.41E-05	-3.57	**CYFIP2**
A_24_P382319	6.29E-03	3.02E-03	-4.19	CEACAM1
A_23_P421483	1.08E-03	3.88E-04	6.26	CEACAM6
A_23_P128974	2.76E-02	1.62E-02	2.18	BATF
A_23_P120744	4.13E-05	6.30E-06	-3.48	MCM3AP
A_32_P15017	1.40E-02	7.49E-03	6.03	RICTOR
A_23_P134176	4.00E-05	6.06E-06	-1.95	**SOD2**
A_23_P111288	1.26E-05	9.81E-07	-2.78	TMEM14C
A_24_P481844	8.19E-05	1.61E-05	4.67	HLA-DMB
A_23_P83192	7.77E-06	3.74E-07	-2.48	PHPT1

A_23_P106080	3.83E-03	1.71E-03	3.14	RNASE7
A_23_P118306	4.87E-05	8.03E-06	-3.06	DNAJA3
A_23_P354341	2.94E-04	8.17E-05	3.01	CD160
A_32_P67533	6.00E-06	2.15E-07	-1.48	L3MBTL3
A_23_P166280	3.16E-05	4.26E-06	-2.69	ICOSLG
A_23_P156687	4.27E-05	6.63E-06	3.03	CFB
A_23_P166087	1.22E-05	9.15E-07	-3.54	RASSF2
A_24_P322741	1.32E-03	4.91E-04	-2.48	IL10RB
A_23_P45059	8.71E-06	5.04E-07	-4.49	DOCK1
A_23_P31188	2.33E-05	2.66E-06	3.39	GIMAP1
A_32_P310335	3.10E-04	8.71E-05	-2.14	JAM2
A_24_P233570	4.77E-05	7.78E-06	-3.34	PSMB1
A_24_P85317	4.38E-05	6.86E-06	3.07	CHD2
A_23_P201193	8.18E-04	2.80E-04	2.70	TSPAN2
A_24_P98109	5.45E-04	1.74E-04	-2.50	SNX10
A_24_P13032	1.47E-04	3.42E-05	-2.11	UBE2D1
A_23_P219144	3.09E-05	4.13E-06	-2.85	CLTA
A_24_P940815	3.39E-03	1.48E-03	-2.16	SGPL1
A_24_P252497	3.62E-04	1.04E-04	-2.32	TRIB1
A_32_P35969	4.12E-03	1.86E-03	-2.01	CHRFAM7A
A_24_P940006	2.81E-05	3.60E-06	-3.87	EFNB3
A_23_P501996	2.92E-05	3.80E-06	-3.16	UBE2V1
A_23_P34402	2.48E-05	2.98E-06	-3.44	NCSTN
A_23_P99942	1.66E-03	6.45E-04	-2.06	LRRK1
A_23_P143526	3.21E-06	6.82E-08	-3.29	**S100B**
A_32_P11471	4.70E-05	7.64E-06	-3.35	FAU
A_24_P273972	2.79E-02	1.64E-02	4.59	CFH
A_24_P355876	4.10E-04	1.22E-04	-3.11	TMBIM6
A_24_P381625	1.98E-05	2.05E-06	-2.27	PSMC6
A_23_P71268	1.55E-03	5.97E-04	3.23	AZGP1
A_23_P7185	6.80E-05	1.25E-05	5.34	STAP1
A_23_P86917	5.09E-05	8.46E-06	-2.49	FADD
A_23_P107735	1.07E-04	2.32E-05	-3.18	CD79A
A_24_P319364	2.46E-05	2.90E-06	-1.86	F11R
A_24_P38951	9.52E-04	3.34E-04	-3.53	RELT
A_23_P252193	5.37E-05	9.20E-06	-3.68	ITGA9
A_23_P164307	1.96E-04	4.82E-05	-3.63	MINK1
A_32_P26092	9.78E-04	3.47E-04	1.82	TNFRSF4
A_23_P29566	3.42E-02	2.07E-02	1.85	ARPC4
A_23_P215599	7.53E-06	3.44E-07	-3.11	SRPK2
A_23_P201551	1.03E-05	6.92E-07	-3.37	VAV3
A_23_P5761	4.65E-04	1.44E-04	-3.24	NFE2L2
A_23_P124642	2.00E-05	2.07E-06	-2.91	RASGRP1
A_23_P212508	4.35E-05	6.78E-06	-3.78	TF
A_23_P165691	7.35E-06	3.30E-07	-2.96	PSMD14
A_23_P206396	2.98E-05	3.92E-06	-2.25	CKLF
A_23_P10127	2.80E-05	3.54E-06	-3.84	SFRP1
A_24_P184031	8.21E-06	4.26E-07	-2.08	PHPT1
A_23_P87310	4.74E-04	1.47E-04	-2.20	LMO1
A_24_P246787	2.54E-03	1.06E-03	-2.33	REST

A_23_P54006	3.11E-06	6.10E-08	-2.60	HECTD1
A_23_P26847	2.37E-04	6.12E-05	-2.09	SOX9
A_23_P370005	2.44E-03	1.01E-03	2.27	RIPK1
A_23_P69058	1.67E-06	1.73E-08	-3.14	MLH1
A_23_P359091	5.39E-03	2.53E-03	1.67	SMAD3
A_23_P55251	7.38E-03	3.61E-03	2.74	ITGA3
A_24_P72420	3.26E-03	1.41E-03	-1.98	ELMO1
A_23_P19352	1.87E-04	4.54E-05	2.54	CNPY3
A_23_P148047	4.51E-03	2.07E-03	2.27	PTGER4
A_23_P500300	7.42E-05	1.41E-05	4.11	TRIM15
A_24_P48403	7.98E-06	3.92E-07	-3.60	YES1
A_23_P372874	1.87E-04	4.54E-05	-2.40	S100A13
A_23_P70867	1.71E-03	6.69E-04	-2.01	SBDS
A_24_P334445	3.83E-05	5.69E-06	-2.91	MAEA
A_23_P92623	4.32E-04	1.31E-04	-3.55	**PPP3CA**
A_23_P59005	1.64E-06	1.41E-08	-2.03	TAP1
A_24_P29876	6.78E-06	2.71E-07	-2.55	AGPAT5
A_23_P369960	3.84E-04	1.12E-04	3.58	NCAPH2
A_23_P97265	6.60E-05	1.20E-05	-2.88	GPATCH4
A_24_P89708	4.41E-04	1.34E-04	3.77	IMPDH1
A_23_P132444	3.81E-05	5.63E-06	-3.25	TCEA1
A_32_P820503	5.35E-06	1.77E-07	-3.28	FTH1
A_24_P148762	3.66E-04	1.06E-04	5.26	IL1RAP
A_24_P169234	2.30E-03	9.45E-04	2.13	ZAP70
A_24_P941824	2.12E-06	3.01E-08	-2.66	KIF3B
A_23_P154840	8.45E-05	1.68E-05	-4.56	**SOD1**
A_24_P418809	8.56E-06	4.83E-07	-3.04	GNAS
A_24_P100419	2.68E-04	7.23E-05	10.63	EDA
A_32_P197489	6.92E-05	1.29E-05	-3.40	KLF13
A_24_P116017	1.49E-03	5.71E-04	3.86	PSMD9
A_24_P146892	2.68E-03	1.13E-03	-2.53	ORAI1
A_24_P173823	7.98E-06	3.88E-07	-2.58	PBX1
A_23_P125423	2.56E-04	6.80E-05	-2.19	C1R
A_24_P13230	8.79E-05	1.78E-05	-2.54	RAB6A
A_23_P113311	4.29E-05	6.67E-06	-3.74	P4HTM
A_23_P140807	8.53E-06	4.68E-07	-2.86	PSMB10
A_23_P364517	8.71E-05	1.76E-05	-3.32	SELK
A_23_P393425	1.21E-03	4.46E-04	-2.01	PAPD4
A_23_P75921	6.72E-04	2.22E-04	-1.59	TRAF6
A_23_P55649	2.55E-02	1.49E-02	-2.46	FPR2
A_32_P7118	8.12E-06	4.07E-07	-2.88	PSMC6
A_24_P337592	9.84E-03	5.01E-03	1.81	BTN2A2
A_23_P67669	1.57E-05	1.44E-06	2.44	PGLYRP2
A_24_P381962	7.93E-05	1.54E-05	-2.94	AP1G1
A_23_P35906	3.22E-05	4.41E-06	-1.95	CASP4
A_23_P55477	4.54E-03	2.08E-03	-1.98	ADORA2B
A_24_P299507	3.03E-04	8.48E-05	3.02	REST
A_24_P326082	1.17E-05	8.46E-07	-2.43	HLA-E
A_23_P49021	1.02E-05	6.72E-07	-3.21	WDR61
A_24_P286465	1.17E-04	2.58E-05	-2.78	PURB

A_23_P502170	1.50E-02	8.13E-03	2.61	DYNC2LI1
A_23_P351215	1.11E-05	7.67E-07	9.48	SKIL
A_23_P126992	3.61E-04	1.04E-04	-2.40	ABI1
A_23_P51361	1.02E-04	2.18E-05	3.18	TXLNA
A_32_P12639	9.94E-07	3.18E-09	-2.94	PSMD12
A_24_P298409	1.11E-05	7.67E-07	-2.86	HLA-C
A_23_P34644	1.11E-05	7.79E-07	2.08	FCGR2B
A_23_P404536	6.77E-05	1.25E-05	1.70	ENPP3
A_24_P381199	4.63E-04	1.43E-04	6.66	TRIM6
A_23_P146468	1.41E-05	1.15E-06	5.75	VAV2
A_24_P172304	3.26E-05	4.49E-06	3.34	ADAMTS13
A_23_P41159	3.77E-03	1.68E-03	2.85	PPP4R2
A_24_P246943	1.66E-05	1.59E-06	-3.70	PPIA
A_24_P31275	3.38E-04	9.59E-05	-2.83	ATP1B2
A_24_P159866	9.78E-03	4.97E-03	3.27	STK11
A_23_P88781	8.52E-05	1.71E-05	-2.95	CIAPIN1
A_24_P235248	1.22E-04	2.72E-05	-3.19	PTPRZ1
A_23_P203900	1.37E-04	3.12E-05	-3.51	SCARB1
A_23_P126825	2.40E-05	2.77E-06	-2.35	SLC16A1
A_23_P80382	1.04E-03	3.72E-04	-2.26	PRR5
A_24_P286013	7.91E-06	3.83E-07	-2.37	UBE2K
A_23_P27215	1.79E-05	1.78E-06	-3.27	UBB
A_23_P385690	2.07E-02	1.18E-02	-3.39	WNT3A
A_24_P353638	2.40E-03	9.97E-04	3.57	SLAMF7
A_23_P54878	2.30E-02	1.32E-02	-3.23	ITGAL
A_23_P368624	3.31E-02	2.00E-02	-2.37	CHRNB2
A_23_P73837	1.14E-02	5.90E-03	3.04	TLR8
A_24_P82142	6.05E-06	2.26E-07	-2.05	TCF12
A_24_P115774	2.80E-05	3.55E-06	-1.83	BIRC2
A_23_P100576	2.66E-04	7.16E-05	-2.43	PSMB3
A_24_P940599	4.69E-03	2.16E-03	2.75	SYNCRIP
A_24_P261326	3.93E-05	5.87E-06	-2.44	ACTR1A
A_24_P227211	1.94E-02	1.09E-02	-2.56	KLC2
A_23_P112103	1.79E-03	7.06E-04	2.58	GSDMD
A_24_P228700	1.19E-02	6.20E-03	6.16	THOC5
A_23_P147388	1.30E-05	1.02E-06	-2.74	KIF13B
A_24_P114739	3.51E-06	8.39E-08	-2.98	CAMK2D
A_24_P65779	1.96E-06	2.46E-08	-1.68	UBA52
A_23_P115460	1.01E-06	4.08E-09	-1.92	RPL22
A_32_P9700	2.37E-06	3.82E-08	-2.19	PSMC1
A_23_P502553	4.15E-03	1.88E-03	-2.39	TRIM35
A_24_P251040	4.14E-02	2.58E-02	-2.66	ARTN
A_24_P392201	6.94E-04	2.31E-04	2.68	TBC1D10C
A_23_P59507	1.88E-02	1.05E-02	-2.40	SH2B2
A_23_P34744	7.68E-05	1.48E-05	-2.11	CTSK
A_23_P52425	4.47E-04	1.37E-04	9.41	NKX2-3
A_24_P273666	3.17E-05	4.27E-06	-4.49	GNAS
A_23_P380181	5.02E-05	8.32E-06	-4.25	LMO4
A_23_P8981	1.22E-05	9.21E-07	-2.09	STAR
A_23_P171249	1.67E-06	1.69E-08	-1.84	IGBP1

A_23_P17560	6.07E-03	2.90E-03	6.29	KIF3B
A_32_P69368	3.25E-05	4.46E-06	-2.80	ID2
A_23_P62932	4.10E-04	1.22E-04	-4.28	ATP1B1
A_23_P8513	3.82E-05	5.65E-06	-4.92	SNX10
A_23_P9362	2.40E-05	2.76E-06	-2.78	PSMB7
A_23_P151471	4.59E-06	1.47E-07	-1.99	CUL4A
A_24_P346277	7.30E-06	3.15E-07	-2.09	SRF
A_23_P156531	3.28E-06	7.15E-08	-3.58	PSMB1
A_23_P374844	2.79E-04	7.63E-05	-4.05	GAL
A_23_P169576	1.15E-05	8.18E-07	-3.23	EXOC6
A_23_P49097	2.05E-04	5.09E-05	4.17	IL17C
A_24_P180165	3.26E-03	1.41E-03	3.02	IL1RAP
A_23_P86599	1.80E-02	1.00E-02	-1.92	DMBT1
A_23_P310350	2.25E-04	5.74E-05	-1.82	SHPK
A_23_P24616	2.44E-04	6.34E-05	-3.48	SIAE
A_23_P55373	5.15E-04	1.63E-04	4.70	ALOX15
A_23_P30666	1.56E-05	1.42E-06	-3.33	TNFRSF21
A_23_P113204	6.14E-05	1.10E-05	-4.34	FGF3
A_23_P24389	4.82E-02	3.10E-02	3.01	CCDC88B
A_23_P84565	5.59E-05	9.69E-06	-2.95	POLR3D
A_24_P8892	4.59E-06	1.46E-07	-1.77	HDAC4
A_24_P269814	2.30E-04	5.88E-05	-2.37	PLEKHA1
A_23_P119562	1.15E-02	5.97E-03	1.98	CFD
A_23_P113634	9.31E-04	3.26E-04	-2.12	CBFB
A_23_P131785	6.21E-05	1.12E-05	5.20	BPI
A_23_P6935	3.96E-05	5.94E-06	-2.43	CD47
A_23_P151610	1.26E-06	7.59E-09	-1.90	PSME1
A_23_P41217	1.43E-03	5.38E-04	3.46	CD200R1
A_23_P341471	3.41E-06	8.07E-08	-2.38	UBE2N
A_23_P365817	3.51E-05	5.06E-06	-2.58	PPP1R14B
A_24_P913210	1.09E-02	5.61E-03	-2.45	SLC11A2
A_24_P134266	1.36E-03	5.07E-04	-2.60	BSG
A_23_P101636	2.86E-03	1.22E-03	4.26	KIR3DL2
A_23_P250122	3.55E-05	5.13E-06	-2.35	FAM20C
A_23_P142631	1.60E-06	1.33E-08	-2.36	FKBP1B
A_23_P136635	3.81E-06	1.00E-07	-3.63	AP3B1
A_23_P165028	1.19E-02	6.20E-03	2.03	DAPK3
A_24_P408424	8.40E-06	4.56E-07	-1.90	MYH9
A_23_P397371	1.79E-03	7.06E-04	-2.38	POLM
A_24_P362317	8.53E-06	4.79E-07	-2.34	ADAR
A_23_P10442	3.88E-06	1.10E-07	-4.01	OSBPL1A
A_23_P97054	3.40E-03	1.49E-03	5.70	PLA2G2F
A_23_P203743	1.11E-05	7.77E-07	-1.80	GAB2
A_24_P11506	1.09E-03	3.92E-04	5.70	KYNU
A_24_P402222	2.90E-02	1.72E-02	2.29	HLA-DRB3
A_23_P212500	2.44E-04	6.34E-05	-4.30	TF
A_23_P14019	1.89E-03	7.51E-04	4.41	APOBEC1
A_24_P63950	1.85E-02	1.03E-02	6.42	AP1S1
A_23_P324327	2.33E-05	2.66E-06	-2.84	GPRC5B
A_24_P113109	9.09E-05	1.87E-05	-3.98	UBB

A_23_P349928	1.52E-05	1.37E-06	-3.30	SP100
A_24_P402588	4.64E-05	7.46E-06	-3.75	BCL11A
A_23_P420196	3.08E-03	1.32E-03	-3.23	SOCS1
A_23_P169351	1.01E-04	2.14E-05	-4.46	SH3GL2
A_23_P143365	5.08E-04	1.60E-04	9.18	CST9L
A_23_P17345	1.35E-04	3.06E-05	-2.29	MAFB
A_23_P167081	4.60E-04	1.42E-04	3.15	REST
A_24_P418239	3.88E-06	1.09E-07	-3.54	RPS14
A_23_P138157	2.14E-04	5.38E-05	3.19	OTUD7B
A_23_P397391	2.54E-02	1.47E-02	-2.58	FFAR2
A_24_P124992	2.73E-06	5.07E-08	-3.30	PSMA4
A_23_P90143	7.26E-05	1.37E-05	-2.75	RPL13A
A_23_P92842	2.04E-05	2.14E-06	-2.44	SAR1B
A_24_P500621	2.02E-03	8.09E-04	-2.91	HIPK2
A_32_P95397	2.59E-05	3.19E-06	-3.70	ITGB1
A_32_P208120	3.48E-04	9.95E-05	-2.55	CAMK1D
A_23_P115998	1.22E-03	4.48E-04	3.67	SPI1
A_23_P214554	2.53E-04	6.65E-05	5.06	TRIM15
A_23_P150286	1.32E-05	1.06E-06	-2.83	PSMA1
A_23_P350467	8.42E-05	1.67E-05	16.04	MAPKAPK2
A_23_P428819	2.70E-06	4.64E-08	-2.85	AKIRIN2
A_32_P193322	7.09E-06	2.99E-07	-2.45	RICTOR
A_23_P164528	9.05E-05	1.85E-05	-2.01	WDR7
A_24_P95038	1.16E-04	2.55E-05	-4.17	PPIA
A_23_P250671	1.06E-04	2.29E-05	-2.82	**GPX1**
A_23_P50146	4.68E-02	3.00E-02	-2.27	SIGLEC15
A_32_P58280	3.22E-04	9.12E-05	2.36	EXOC6
A_32_P128656	1.07E-02	5.49E-03	3.24	MID1
A_23_P123563	1.00E-04	2.11E-05	-3.53	RPS6
A_23_P44037	2.97E-05	3.90E-06	-3.02	ACTG1
A_23_P6596	7.62E-05	1.46E-05	6.79	HES1
A_23_P169189	2.06E-05	2.17E-06	-2.41	IKBKAP
A_23_P98410	2.94E-05	3.83E-06	3.73	CD3G
A_23_P131024	2.26E-04	5.76E-05	4.70	ZBTB32
A_24_P333525	9.01E-06	5.47E-07	-2.30	RABGAP1L
A_24_P58337	2.56E-05	3.12E-06	-2.91	FTH1
A_32_P12820	9.27E-05	1.92E-05	-3.67	YWHAZ
A_23_P324813	2.52E-04	6.61E-05	2.53	BCL6B
A_23_P353478	5.56E-03	2.62E-03	-2.60	CIITA
A_23_P121533	2.80E-05	3.57E-06	-2.49	SPON2
A_32_P74983	6.01E-05	1.06E-05	-2.39	MICB
A_23_P103104	4.18E-03	1.89E-03	-2.82	MFNG
A_23_P382081	2.56E-03	1.08E-03	3.61	SPEF2
A_23_P59069	1.04E-05	7.02E-07	-1.47	HIST1H2BO
A_23_P147874	3.27E-05	4.52E-06	-2.36	CHID1
A_23_P103414	2.04E-06	2.83E-08	-2.86	YTHDF2
A_23_P373017	1.99E-02	1.12E-02	-1.90	CCL3
A_23_P139585	2.17E-03	8.79E-04	-2.06	PDE1B
A_24_P19983	8.84E-05	1.80E-05	3.07	BCL10
A_24_P237559	3.17E-03	1.37E-03	3.77	LNPEP

A_23_P55936	1.01E-04	2.14E-05	-2.42	FCGRT
A_23_P208482	1.03E-04	2.20E-05	-4.14	CLEC4M
A_23_P70968	1.78E-02	9.88E-03	2.98	HOXA7
A_23_P26771	4.62E-04	1.43E-04	2.82	CD300C
A_23_P210763	4.20E-02	2.63E-02	1.81	JAG1
A_23_P126803	2.14E-04	5.37E-05	-4.28	ARPC5
A_24_P931443	7.62E-06	3.56E-07	-3.42	GPR68
A_24_P353794	4.32E-04	1.31E-04	1.73	GALNT2
A_23_P5550	3.69E-05	5.41E-06	-3.48	PUM2
A_24_P919330	9.25E-06	5.82E-07	-2.22	FTH1
A_23_P425750	2.34E-03	9.68E-04	-2.98	ARMC6
A_23_P54313	1.27E-02	6.67E-03	-2.87	CD276
A_23_P23829	1.94E-02	1.09E-02	-2.69	CD34
A_23_P40527	9.55E-04	3.36E-04	8.63	TBX1
A_23_P503072	1.46E-04	3.38E-05	2.12	CCL28
A_23_P98022	1.89E-03	7.53E-04	-2.11	SIRT1
A_23_P155257	2.28E-04	5.82E-05	-2.43	FOXP1
A_23_P142835	1.27E-04	2.85E-05	-2.36	**DCTN1**
A_24_P914134	5.30E-05	8.99E-06	-7.64	PRNP
A_23_P15326	1.10E-03	4.00E-04	-2.06	TRIM25
A_23_P128744	2.73E-02	1.60E-02	-2.11	BDKRB1
A_23_P155677	3.85E-05	5.73E-06	-1.60	UBE2K
A_23_P210482	2.68E-04	7.26E-05	-2.42	ADA
A_23_P214046	8.99E-05	1.84E-05	-2.42	FBXW11
A_23_P254111	2.22E-05	2.44E-06	-3.83	FBXO9
A_23_P115732	8.53E-06	4.77E-07	-2.75	BMI1
A_23_P204087	2.70E-02	1.58E-02	-4.58	OAS2
A_24_P365327	7.12E-05	1.33E-05	-1.61	LSM14A
A_23_P323823	1.95E-05	2.00E-06	3.67	HIST1H2BA
A_23_P166459	5.62E-05	9.76E-06	-2.52	LGALS1
A_24_P72479	3.88E-06	1.06E-07	-2.78	ARPC1A
A_23_P31097	2.22E-06	3.42E-08	-3.11	OSTM1
A_23_P33752	7.09E-06	2.98E-07	-2.02	SLC25A6
A_23_P304287	8.89E-06	5.29E-07	-2.03	PSMC2
A_23_P407012	8.18E-05	1.60E-05	2.15	CSF1
A_23_P87513	1.04E-02	5.33E-03	-2.85	ATP6V0A2
A_24_P324838	8.10E-04	2.76E-04	2.25	IGHD
A_23_P133424	3.28E-05	4.54E-06	-3.76	SKP1
A_23_P167509	6.16E-05	1.11E-05	-2.66	**CYFIP2**
A_23_P329740	1.96E-04	4.83E-05	-3.51	UBC
A_23_P19650	4.45E-04	1.36E-04	-2.74	VIP
A_24_P133542	1.96E-06	2.39E-08	3.32	PML
A_23_P433760	2.19E-03	8.93E-04	-3.37	SPN
A_23_P27677	3.68E-02	2.27E-02	2.25	IRF3
A_24_P681301	9.83E-04	3.50E-04	-3.06	UBC
A_23_P64898	6.41E-03	3.09E-03	-2.40	KLRG1
A_32_P6344	1.70E-05	1.65E-06	-4.29	MAP2K4
A_23_P257250	8.82E-04	3.04E-04	5.13	KIAA0922
A_24_P889720	4.25E-05	6.57E-06	-3.39	UBC
A_24_P194845	3.88E-06	1.06E-07	-2.40	RAC1

A_24_P270814	4.45E-04	1.36E-04	-2.71	CRK
A_24_P673786	1.09E-05	7.43E-07	-2.76	PIP4K2A
A_23_P123503	8.87E-04	3.07E-04	6.08	TRIB1
A_23_P212768	6.94E-05	1.29E-05	-2.92	ADD1
A_24_P682285	6.08E-05	1.08E-05	1.87	HSPA1A
A_24_P29401	3.47E-03	1.52E-03	-2.39	PIK3R1
A_24_P344307	3.82E-04	1.12E-04	-1.78	PSME3
A_23_P146990	2.20E-05	2.39E-06	-3.00	WWP1
A_24_P380348	3.37E-02	2.03E-02	-1.61	SMAP1
A_23_P84952	4.66E-02	2.99E-02	1.61	TFE3
A_23_P216812	8.89E-06	5.34E-07	9.00	CDKN2B
A_23_P218025	3.96E-05	5.94E-06	-3.60	KIF5A
A_23_P158829	1.66E-04	3.94E-05	-2.09	ARRB2
A_24_P411561	5.91E-03	2.81E-03	2.77	HAVCR2
A_32_P168349	3.80E-02	2.35E-02	-2.67	C6orf25
A_23_P9232	1.85E-05	1.86E-06	3.44	GCNT1
A_24_P940909	2.03E-03	8.14E-04	2.77	TTC7A
A_24_P335781	2.53E-04	6.69E-05	-1.97	ADCYAP1
A_23_P53856	6.23E-06	2.38E-07	-1.89	N4BP2L2
A_23_P63017	2.24E-05	2.47E-06	-2.77	LGALS8
A_23_P74928	4.70E-05	7.66E-06	2.92	MR1
A_23_P94338	1.74E-05	1.71E-06	-3.17	ENPP2
A_24_P69538	1.47E-02	7.93E-03	2.13	TLR4
A_24_P598836	1.21E-04	2.70E-05	-2.21	ITGB1
A_24_P87931	1.58E-05	1.46E-06	-1.55	APOL1
A_24_P335656	2.79E-02	1.64E-02	-3.04	SECTM1
A_23_P51996	1.58E-05	1.45E-06	-2.53	STXBP3
A_24_P411186	4.00E-04	1.18E-04	-5.34	BCL11A
A_23_P80528	1.38E-03	5.16E-04	2.99	TIGIT
A_24_P792734	4.53E-05	7.19E-06	-3.85	PSMC6
A_24_P226554	3.53E-06	8.86E-08	-2.29	ACTB
A_23_P6943	2.46E-03	1.02E-03	-2.65	GPR15
A_23_P89192	4.64E-05	7.46E-06	3.11	EPX
A_24_P341897	5.12E-03	2.39E-03	-2.27	ACVR1B
A_24_P801264	2.00E-04	4.94E-05	-2.43	HMGB1
A_23_P332820	2.74E-02	1.61E-02	-2.78	IL17A
A_23_P354074	4.93E-04	1.55E-04	-2.42	LYST
A_23_P118888	3.13E-06	6.35E-08	-1.90	PAFAH1B1
A_23_P124542	1.12E-04	2.44E-05	-4.19	CR2
A_24_P279704	4.94E-05	8.17E-06	-3.48	MAG
A_24_P162073	3.47E-02	2.11E-02	1.91	BCR
A_23_P46482	4.85E-05	7.98E-06	3.83	IL20
A_23_P331748	2.49E-03	1.04E-03	1.97	CD33
A_23_P10232	4.83E-02	3.11E-02	-2.72	BANK1
A_23_P343303	6.11E-03	2.92E-03	-2.29	GFI1B
A_23_P332713	3.41E-03	1.49E-03	-2.52	ZFR2
A_23_P256470	1.33E-04	2.99E-05	-3.51	NPY
A_23_P370434	2.98E-05	3.92E-06	-3.18	C1QBP
A_23_P125977	2.17E-03	8.78E-04	2.72	C1QC
A_23_P419947	1.02E-03	3.63E-04	-2.37	MLF1

A_24_P231513	1.24E-05	9.54E-07	-2.16	ACTR3
A_24_P936758	3.60E-03	1.59E-03	-3.76	IGF2
A_23_P211985	8.64E-03	4.33E-03	2.07	SNRK
A_23_P166219	3.36E-02	2.03E-02	4.46	GABPA
A_32_P57870	1.50E-04	3.49E-05	-3.47	PSMC1
A_24_P82200	1.24E-02	6.50E-03	1.88	MEIS2
A_24_P53595	1.43E-03	5.40E-04	6.05	GNAS
A_23_P167674	7.39E-05	1.40E-05	-2.10	F12
A_23_P9688	5.91E-05	1.03E-05	-2.80	DYNC1LI2
A_23_P210939	3.20E-05	4.36E-06	-2.68	EIF6
A_23_P393598	3.89E-06	1.14E-07	2.77	CD276
A_23_P171296	2.60E-05	3.20E-06	-2.96	MPP1
A_24_P76267	1.07E-03	3.84E-04	-2.10	GGT1
A_24_P938293	4.65E-05	7.51E-06	-2.00	HES1
A_23_P82929	7.74E-06	3.68E-07	-2.97	NOV
A_23_P65031	2.06E-05	2.18E-06	-4.25	DYNLL1
A_23_P65370	1.04E-05	7.04E-07	-3.50	GLRX5
A_23_P35755	4.11E-02	2.57E-02	-3.79	TYR
A_24_P472055	4.59E-03	2.11E-03	-2.75	RNF125
A_23_P258837	3.81E-04	1.11E-04	5.28	ORAI1
A_23_P213204	1.26E-06	6.76E-09	-1.79	WHSC1
A_23_P76538	9.98E-05	2.09E-05	-3.56	TESC
A_23_P166371	8.92E-03	4.48E-03	-2.19	VPREB3
A_23_P111381	5.74E-06	1.96E-07	-2.66	ATG5
A_32_P170003	3.87E-04	1.14E-04	-2.19	TRAF3IP1
A_32_P162726	1.77E-03	6.96E-04	-2.82	EXOSC6
A_23_P122174	4.21E-02	2.64E-02	-2.09	XRCC4
A_23_P99980	6.90E-05	1.28E-05	-3.15	HMGB1
A_23_P153372	2.63E-04	7.07E-05	-2.57	HSH2D
A_23_P97632	7.19E-06	3.05E-07	-2.88	EPRS
A_24_P236949	3.87E-02	2.40E-02	2.43	C19orf66
A_23_P159956	2.90E-02	1.72E-02	1.53	MID2
A_23_P254120	1.40E-04	3.19E-05	-3.65	FBXO9
A_23_P120002	4.65E-05	7.49E-06	2.41	SP110
A_23_P71624	1.97E-02	1.11E-02	-1.86	PAX5
A_23_P205929	9.39E-05	1.95E-05	-2.97	DPP8
A_32_P210252	4.59E-05	7.34E-06	-2.83	RPL22
A_23_P75453	2.32E-04	5.96E-05	-2.49	MEN1
A_24_P916522	4.40E-02	2.78E-02	-2.03	PAX5
A_24_P362737	2.67E-05	3.32E-06	-2.99	FOXP1
A_23_P134851	2.46E-04	6.42E-05	2.45	DOK2
A_23_P107351	1.34E-04	3.03E-05	-3.31	NLRP1
A_24_P13381	1.16E-03	4.21E-04	2.51	TRPV4
A_23_P59426	8.87E-04	3.07E-04	-1.72	PAXIP1
A_24_P935026	1.51E-04	3.52E-05	-2.95	STK4
A_32_P342064	1.14E-04	2.49E-05	-5.33	FTH1
A_23_P8281	1.14E-05	8.08E-07	-2.52	IFNGR1
A_23_P5273	7.32E-04	2.45E-04	4.97	SBNO2
A_23_P348636	1.25E-02	6.55E-03	1.76	FOXJ1
A_23_P140648	2.81E-04	7.69E-05	-2.03	CYFIP1

A_23_P29773	4.68E-02	3.00E-02	-2.01	LAMP3
A_24_P402242	4.70E-05	7.65E-06	3.34	COL3A1
A_24_P374244	5.91E-03	2.81E-03	3.68	GATA1
A_23_P250358	8.52E-03	4.25E-03	6.15	HERC6
A_24_P278637	1.27E-02	6.68E-03	4.10	FADD
A_32_P97489	4.00E-05	6.02E-06	-2.22	YWHAZ
A_32_P43217	5.11E-05	8.59E-06	-4.01	PSMA6
A_23_P145238	8.65E-04	2.97E-04	-2.52	HIST1H2BK
A_23_P35256	9.04E-04	3.14E-04	2.94	POLR3C
A_24_P370952	5.09E-05	8.49E-06	4.30	LAX1
A_23_P120325	1.29E-04	2.90E-05	-3.24	KIF3C
A_23_P211957	9.15E-04	3.19E-04	-2.06	TGFBR2
A_23_P60742	9.52E-04	3.35E-04	2.60	MYLPF
A_23_P39665	2.76E-02	1.62E-02	-1.87	SLC11A1
A_24_P873764	2.11E-04	5.27E-05	-2.42	BCR
A_23_P4308	5.55E-04	1.78E-04	2.06	PLD2
A_23_P17548	2.54E-04	6.72E-05	4.16	DEFB118
A_23_P156017	1.21E-04	2.69E-05	-2.81	GOLPH3
A_23_P52266	1.44E-05	1.20E-06	-3.18	IFIT1
A_24_P414371	2.51E-05	3.06E-06	-3.53	**PPP3CA**
A_24_P76759	1.64E-04	3.90E-05	2.62	FARP2
A_24_P124624	2.72E-03	1.16E-03	-1.83	OLR1
A_23_P161983	1.34E-02	7.10E-03	-2.35	MRGPRX4
A_23_P63158	1.75E-02	9.71E-03	4.71	SPTA1
A_23_P54573	1.03E-03	3.70E-04	-3.95	ZFPM1
A_23_P200560	3.61E-05	5.25E-06	-2.76	CDC42
A_23_P76059	1.08E-05	7.32E-07	-2.35	DCTN2
A_24_P173325	1.44E-05	1.22E-06	-3.50	UBA52
A_23_P128930	1.22E-05	9.24E-07	-2.23	PSMC6
A_23_P345591	9.05E-05	1.86E-05	-1.93	PSMA2
A_23_P2601	2.36E-05	2.71E-06	-3.43	HSP90B1
A_23_P214499	5.10E-05	8.54E-06	3.19	BTN3A1
A_24_P272088	3.08E-05	4.10E-06	1.78	SPTB
A_23_P121459	8.52E-05	1.71E-05	12.88	THPO
A_23_P302386	6.19E-04	2.00E-04	3.75	HLA-DOA
A_23_P147450	1.26E-06	9.54E-09	-2.53	SPG21
A_23_P116898	4.12E-04	1.23E-04	-2.89	A2M
A_23_P257956	2.46E-05	2.92E-06	-2.91	ILF2
A_24_P45451	1.91E-04	4.66E-05	3.97	SLAMF6
A_24_P252945	2.32E-03	9.56E-04	5.95	CXCR5
A_23_P141917	2.45E-03	1.02E-03	3.44	TYK2
A_24_P124032	4.59E-06	1.45E-07	-2.22	RIPK2
A_32_P416583	3.50E-02	2.13E-02	-2.21	NLRC5
A_23_P38959	8.64E-03	4.33E-03	2.12	VAV1
A_23_P25735	7.62E-06	3.57E-07	-3.50	PSMA6
A_23_P102202	3.40E-06	7.88E-08	-1.88	MSH6
A_23_P819	1.44E-03	5.44E-04	-1.88	ISG15
A_23_P48088	3.02E-04	8.42E-05	-2.52	CD27
A_23_P300100	1.10E-03	3.99E-04	2.29	PLA2G2D
A_23_P39386	1.41E-03	5.29E-04	-2.61	HCST

A_23_P219060	1.65E-02	9.04E-03	-2.23	GPSM3
A_23_P356684	5.29E-06	1.73E-07	-3.51	ANLN
A_32_P111565	1.43E-05	1.19E-06	-3.02	FTH1
A_32_P880454	3.26E-05	4.50E-06	-4.18	APLF
A_23_P162866	4.21E-06	1.27E-07	-3.31	HSP90AA1
A_32_P24581	2.00E-05	2.08E-06	-2.81	RPS27A
A_24_P339944	6.58E-04	2.17E-04	2.03	PDGFB
A_23_P137806	3.88E-03	1.73E-03	-2.61	MPL
A_24_P125283	7.26E-05	1.37E-05	-2.95	HDAC5
A_23_P118544	3.49E-05	5.01E-06	-3.90	CLTC
A_23_P25433	2.50E-03	1.04E-03	-1.87	C12orf4
A_32_P199252	4.45E-04	1.36E-04	-3.43	HSP90AA1
A_23_P6130	6.00E-06	2.23E-07	2.18	PAX1
A_23_P171074	3.51E-05	5.07E-06	-4.27	ITM2A
A_23_P207842	3.53E-06	8.79E-08	-5.02	RARA
A_23_P103398	2.24E-04	5.69E-05	-2.65	PSEN2
A_23_P119943	4.74E-04	1.48E-04	-2.56	IGFBP2
A_24_P141332	6.61E-05	1.21E-05	-2.87	CAMK2G
A_32_P175198	1.57E-04	3.69E-05	-3.25	ACTG1
A_24_P275984	1.87E-03	7.41E-04	7.87	ZNF616
A_23_P138635	2.48E-05	2.98E-06	-5.72	BNIP3
A_23_P88201	2.15E-06	3.18E-08	-2.69	PPP2R3C
A_23_P133245	7.74E-06	3.70E-07	-2.10	IK
A_24_P311917	6.56E-04	2.16E-04	2.28	BTN3A3
A_23_P256473	3.12E-04	8.76E-05	-2.41	SEMA3C
A_24_P63019	2.60E-03	1.09E-03	4.54	IL1R2
A_23_P210100	4.29E-05	6.66E-06	-2.64	CYP26B1
A_23_P502174	3.28E-05	4.56E-06	-3.57	DYNC2LI1
A_23_P79732	1.35E-04	3.06E-05	-3.38	RPS27A
A_23_P38894	2.19E-05	2.36E-06	-2.43	C19orf66
A_23_P163814	3.35E-04	9.50E-05	-3.83	ATXN1L
A_23_P20814	6.17E-04	2.00E-04	1.61	DDX58
A_24_P340679	9.05E-06	5.55E-07	-3.65	PPIA
A_23_P35970	2.68E-04	7.24E-05	-1.67	SLC37A4
A_23_P389919	1.44E-02	7.76E-03	-1.81	WHSC1
A_24_P82106	5.89E-04	1.89E-04	4.73	MMP14
A_23_P367995	7.57E-03	3.72E-03	3.52	PSMA8
A_23_P146922	1.43E-02	7.72E-03	1.94	GAS6
A_23_P22444	2.55E-02	1.48E-02	1.68	CFP
A_23_P22378	1.33E-04	3.01E-05	2.96	SOX11
A_23_P115223	1.81E-05	1.82E-06	-3.07	HAX1
A_24_P373976	3.14E-04	8.84E-05	14.21	SDC4
A_23_P99771	7.53E-05	1.44E-05	-3.81	PNMA1
A_24_P166431	1.71E-03	6.72E-04	-2.70	TRIM10
A_24_P216165	2.09E-04	5.23E-05	-4.75	CEBPA
A_23_P10815	9.43E-06	6.09E-07	-3.60	PUM1
A_32_P186921	3.29E-05	4.59E-06	-1.98	ZNF616
A_23_P29953	1.71E-05	1.68E-06	-11.09	IL15
A_23_P218369	2.87E-06	5.47E-08	7.86	CCL15
A_24_P154037	1.41E-05	1.16E-06	-2.23	IRS2

A_24_P105721	4.12E-04	1.23E-04	9.19	CREB1
A_24_P10890	9.50E-05	1.97E-05	-3.61	PRR5
A_23_P106016	2.56E-04	6.84E-05	-2.34	PRKD1
A_23_P250904	1.09E-03	3.95E-04	-2.10	UBQLN1
A_23_P337242	1.26E-06	8.32E-09	1.92	TGFBR2
A_24_P94916	1.78E-02	9.88E-03	1.90	LST1
A_23_P404162	1.57E-02	8.56E-03	-3.41	HDAC9
A_24_P284783	1.68E-05	1.62E-06	1.91	PPIL2
A_32_P46765	4.41E-05	6.93E-06	-4.23	C12orf29
A_23_P218685	2.34E-03	9.62E-04	-2.29	UBE2V1
A_23_P311875	4.33E-02	2.72E-02	-1.86	CD6
A_23_P259863	1.79E-02	9.96E-03	-4.14	CD177
A_23_P31323	2.70E-06	4.61E-08	-2.25	ACTB
A_23_P321855	6.99E-04	2.33E-04	-2.35	ARHGEF7
A_23_P165879	2.49E-05	2.99E-06	-2.47	RAB10
A_32_P80678	6.14E-03	2.94E-03	2.51	ADAM9
A_23_P761	5.15E-05	8.67E-06	-3.73	PSMB4
A_23_P130169	8.40E-06	4.55E-07	-4.94	TBKBP1
A_24_P91272	4.85E-02	3.12E-02	-2.55	SOCS6
A_23_P214876	6.14E-05	1.10E-05	-1.87	JARID2
A_23_P167452	1.01E-04	2.12E-05	4.13	ERAP1
A_24_P116351	1.52E-05	1.36E-06	-2.75	SIVA1
A_23_P151930	9.09E-03	4.58E-03	-2.13	TCF12
A_23_P26815	8.42E-05	1.67E-05	-3.00	RILP
A_23_P217384	6.57E-05	1.19E-05	-2.36	AP1S2
A_23_P30913	1.65E-05	1.56E-06	-2.93	HLA-DPA1
A_24_P566891	8.89E-06	5.32E-07	-3.28	ARF1
A_23_P413641	6.00E-06	2.20E-07	-1.89	PREX1
A_24_P22488	6.21E-04	2.01E-04	2.78	AXIN1
A_24_P363802	5.55E-04	1.78E-04	-2.48	PSMD5
A_32_P134968	1.34E-03	4.98E-04	-3.06	SPTB
A_23_P216257	2.25E-05	2.50E-06	-3.74	TPD52
A_23_P215491	6.56E-03	3.17E-03	-3.02	CCL24
A_23_P56188	3.35E-05	4.76E-06	-3.73	UBA52
A_23_P258570	1.96E-06	2.30E-08	-3.92	PSMD10
A_23_P132316	2.28E-05	2.54E-06	3.36	APOBEC3D
A_23_P162846	1.93E-04	4.73E-05	-3.16	LAMP1
A_24_P849801	9.85E-05	2.06E-05	-2.28	RPL22
A_23_P98884	3.40E-03	1.49E-03	-2.63	RNF41
A_23_P414884	4.28E-04	1.29E-04	-3.28	CRHR1
A_24_P399630	5.08E-04	1.60E-04	-2.14	PRKACA
A_23_P128337	7.06E-06	2.89E-07	-5.82	PTMS
A_23_P204304	1.31E-05	1.04E-06	-3.76	PTPRO
A_23_P158239	2.34E-02	1.35E-02	1.84	SHMT2
A_23_P306500	9.83E-06	6.42E-07	-3.10	KRAS
A_23_P201459	2.14E-03	8.63E-04	-2.03	IFI6
A_23_P157478	6.90E-05	1.28E-05	-2.98	ATP6V1H
A_23_P93180	3.29E-03	1.43E-03	2.02	HIST1H2BC
A_23_P134925	1.33E-05	1.07E-06	-2.45	BNIP3L
A_23_P56938	2.16E-02	1.24E-02	2.57	REL

A_23_P501538	7.30E-06	3.22E-07	1.97	HOXA3
A_23_P69493	3.88E-06	1.11E-07	-3.42	RHOA
A_24_P39759	1.41E-05	1.17E-06	1.64	RNF135
A_24_P277456	1.02E-04	2.17E-05	-3.50	UBE2K
A_24_P935437	4.77E-05	7.81E-06	1.92	MBP
A_23_P2097	5.73E-04	1.84E-04	-1.79	TRIM68
A_23_P256561	2.05E-04	5.13E-05	1.64	TLR6
A_23_P207940	5.57E-05	9.61E-06	-1.79	PTPN2

In bold are highlighted candidate genes already known to contribute to ALS susceptibility.

*Gene expression fold-changes are given on a linear scale.

Supplementary Table 5. Neuroinflammatory genes differentially expressed in SALS1 and SALS2.

Probe ID	p Value (Corr)	p Value	Fold Change (SALS1/CTRL$^+$)*	Fold Change (SALS2/CTRL$^+$)*	GENE_SYMBOL
A_23_P89249	7.67E-04	2.59E-04	1.99	1.6245594	ERBB2
A_23_P309739	2.75E-02	1.61E-02	-1.46	-1.5552287	ESR1
A_23_P70095	1.39E-02	7.41E-03	1.69	2.1040883	CD74
A_23_P101992	3.31E-02	1.99E-02	2.44	2.9789243	MARCO
A_23_P103361	1.17E-02	6.08E-03	-1.48	-1.6601439	LCK
A_24_P943283	1.43E-03	5.37E-04	-1.64	-2.3417015	DENND1B
A_24_P254079	6.81E-03	3.31E-03	2.10	2.963726	ICAM5
A_23_P81912	5.03E-06	1.64E-07	2.33	-2.1507022	TUBB
A_23_P151294	1.01E-06	3.77E-09	-1.80	1.9352487	IFNG
A_23_P140916	4.24E-06	1.30E-07	2.17	-2.0372417	GLG1
A_23_P29994	8.12E-06	4.08E-07	2.08	-2.1523817	RBPJ
A_23_P42935	1.37E-02	7.30E-03	-1.32	-1.4475981	BRAF
A_32_P120977	8.16E-06	4.15E-07	1.64	-1.6825967	PRELID1
A_23_P121253	1.57E-02	8.57E-03	-1.40	-1.5319853	TNFSF10
A_23_P113716	7.07E-06	2.91E-07	2.11	-2.3847184	HLA-C
A_23_P130815	3.40E-03	1.49E-03	-1.84	-1.9945887	KIR2DS2
A_24_P276853	1.29E-03	4.79E-04	2.01	2.162412	GON4L
A_24_P252078	1.81E-02	1.01E-02	1.63	1.7563841	BTN3A2
A_24_P766716	2.55E-02	1.48E-02	1.72	1.8776253	CMKLR1
A_23_P207582	7.09E-06	2.99E-07	-3.23	3.2294095	CCL16
A_24_P103469	4.54E-03	2.08E-03	1.98	2.8276353	LST1
A_32_P232865	6.76E-03	3.28E-03	3.14	3.7766464	BCR
A_23_P25644	6.68E-07	8.25E-10	1.69	-2.291853	PCID2
A_23_P79161	3.88E-06	1.11E-07	1.54	-1.6402473	PRELID1
A_23_P56195	6.68E-07	1.26E-09	1.58	-2.210804	UBA52
A_23_P203053	2.04E-03	8.20E-04	1.70	2.2072346	NCAM1
A_23_P30122	4.59E-06	1.48E-07	-1.83	2.1090114	IL2
A_23_P169331	1.37E-02	7.28E-03	1.78	1.5586059	TRAF2
A_24_P222655	1.03E-02	5.29E-03	2.90	2.3396752	C1QA
A_32_P217709	4.24E-06	1.29E-07	2.37	-2.2239976	RAC1
A_23_P38346	8.68E-06	4.99E-07	1.98	-2.0749657	DHX58
A_23_P31921	4.35E-03	1.98E-03	2.69	3.2652416	ASS1
A_23_P142380	6.68E-07	9.44E-10	1.79	-1.8057177	AKAP8
A_23_P136325	6.22E-03	2.98E-03	1.62	2.1003823	WIPF2
A_32_P67533	6.00E-06	2.15E-07	1.40	-1.482858	L3MBTL3
A_23_P71268	1.55E-03	5.97E-04	2.25	3.2261822	AZGP1
A_24_P184031	8.21E-06	4.26E-07	2.08	-2.0806754	PHPT1
A_23_P359091	5.39E-03	2.53E-03	1.73	1.6670423	SMAD3
A_23_P59005	1.64E-06	1.41E-08	2.05	-2.0266466	TAP1
A_24_P337592	9.84E-03	5.01E-03	1.61	1.8112911	BTN2A2
A_23_P115460	1.01E-06	4.08E-09	1.75	-1.9200194	RPL22
A_32_P9700	2.37E-06	3.82E-08	1.94	-2.1930177	PSMC1
A_23_P171249	1.67E-06	1.69E-08	1.98	-1.8409251	IGBP1
A_23_P17560	6.07E-03	2.90E-03	3.32	6.288712	KIF3B
A_24_P346277	7.30E-06	3.15E-07	1.93	-2.0923793	SRF
A_24_P8892	4.59E-06	1.46E-07	1.75	-1.7740107	HDAC4
A_23_P119562	1.15E-02	5.97E-03	2.46	1.9826583	CFD

A_23_P151610	1.26E-06	7.59E-09	2.42	-1.9032911	PSME1
A_23_P142631	1.60E-06	1.33E-08	1.92	-2.3611374	FKBP1B
A_24_P402222	2.90E-02	1.72E-02	2.53	2.2931263	HLA-DRB3
A_24_P353794	4.32E-04	1.31E-04	2.05	1.7299206	GALNT2
A_23_P128744	2.73E-02	1.60E-02	-2.15	-2.1131637	BDKRB1
A_23_P33752	7.09E-06	2.98E-07	2.12	-2.0164661	SLC25A6
A_24_P682285	6.08E-05	1.08E-05	3.53	1.8744959	HSPA1A
A_24_P226554	3.53E-06	8.86E-08	2.64	-2.288506	ACTB
A_23_P211985	8.64E-03	4.33E-03	1.87	2.0685003	SNRK
A_24_P82200	1.24E-02	6.50E-03	1.59	1.8813595	MEIS2
A_24_P472055	4.59E-03	2.11E-03	-2.14	-2.748359	RNF125
A_23_P166371	8.92E-03	4.48E-03	-1.99	-2.1929023	VPREB3
A_23_P71624	1.97E-02	1.11E-02	-2.18	-1.8598564	PAX5
A_23_P348636	1.25E-02	6.55E-03	1.91	1.7638527	FOXJ1
A_23_P38959	8.64E-03	4.33E-03	1.66	2.119077	VAV1
A_23_P48088	3.02E-04	8.42E-05	-1.64	-2.5189831	CD27
A_23_P133245	7.74E-06	3.70E-07	1.99	-2.1024985	IK
A_24_P216165	2.09E-04	5.23E-05	-2.49	-4.7468586	CEBPA
A_32_P186921	3.29E-05	4.59E-06	-1.42	-1.9837334	ZNF616
A_23_P337242	1.26E-06	8.32E-09	-1.75	1.9213755	TGFBR2
A_24_P94916	1.78E-02	9.88E-03	1.88	1.896965	LST1
A_23_P31323	2.70E-06	4.61E-08	2.60	-2.2473917	ACTB
A_23_P413641	6.00E-06	2.20E-07	1.78	-1.890054	PREX1
A_23_P158239	2.34E-02	1.35E-02	1.75	1.8354497	SHMT2
A_23_P501538	7.30E-06	3.22E-07	-2.04	1.9682595	HOXA3

*Gene expression fold-changes are given on a linear scale.

Supplementary Table 6. List of genes included in the SALS1-related PPI network.

Degree	Node	Gene name
157	UBC	ubiquitin C
84	TP53	tumor protein p53
79	AKT1	v-akt murine thymoma viral oncogene homolog 1
66	MYC	v-myc myelocytomatosis viral oncogene homolog (avian)
64	CCND1	cyclin D1
64	STAT3	signal transducer and activator of transcription 3 (acute-phase response factor)
63	HSP90AA1	heat shock protein 90kDa alpha (cytosolic), class A member 1
62	JUN	jun proto-oncogene
58	EGFR	epidermal growth factor receptor
56	FOS	FBJ murine osteosarcoma viral oncogene homolog
56	MAPK14	mitogen-activated protein kinase 14
54	RAC1	ras-related C3 botulinum toxin substrate 1 (rho family, small GTP binding protein Rac1)
49	RHOA	ras homolog family member A
47	MAPK1	mitogen-activated protein kinase 1
47	PIK3R1	phosphoinositide-3-kinase, regulatory subunit 1 (alpha)
46	MAPK8	mitogen-activated protein kinase 8
45	NFKB1	nuclear factor of kappa light polypeptide gene enhancer in B-cells 1
45	CTNNB1	catenin (cadherin-associated protein), beta 1, 88kDa
45	PTPN11	protein tyrosine phosphatase, non-receptor type 11
44	FYN	FYN oncogene related to SRC, FGR, YES
44	STAT1	signal transducer and activator of transcription 1, 91kDa
43	HRAS	v-Ha-ras Harvey rat sarcoma viral oncogene homolog
43	UBB	ubiquitin B
42	EP300	E1A binding protein p300
41	CREBBP	CREB binding protein
40	ERBB2	v-erb-b2 erythroblastic leukemia viral oncogene homolog 2, neuro/glioblastoma derived oncogene homolog (avian)
40	MTOR	mechanistic target of rapamycin (serine/threonine kinase)
39	ABL1	c-abl oncogene 1, non-receptor tyrosine kinase
39	AR	androgen receptor
39	RELA	v-rel reticuloendotheliosis viral oncogene homolog A (avian)
39	VEGFA	vascular endothelial growth factor A
39	UBA52	ubiquitin A-52 residue ribosomal protein fusion product 1
38	CDH1	cadherin 1, type 1, E-cadherin (epithelial)
38	GRB2	growth factor receptor-bound protein 2
38	HIF1A	hypoxia inducible factor 1, alpha subunit (basic helix-loop-helix transcription factor)
36	EGR1	early growth response 1
36	MAPK3	mitogen-activated protein kinase 3
35	RAC3	ras-related C3 botulinum toxin substrate 3 (rho family, small GTP binding protein Rac3)
34	FOXO3	forkhead box O3
34	HSPA8	heat shock 70kDa protein 8
34	CDKN1B	cyclin-dependent kinase inhibitor 1B (p27, Kip1)
34	SMAD3	SMAD family member 3
33	JAK2	Janus kinase 2
33	NRAS	neuroblastoma RAS viral (v-ras) oncogene homolog
33	CDC42	cell division cycle 42 (GTP binding protein, 25kDa)
33	PTK2	PTK2 protein tyrosine kinase 2
32	NOTCH1	notch 1
32	BCL2L1	BCL2-like 1
30	KRAS	v-Ki-ras2 Kirsten rat sarcoma viral oncogene homolog
30	LYN	v-yes-1 Yamaguchi sarcoma viral related oncogene homolog
30	HSP90AB1	heat shock protein 90kDa alpha (cytosolic), class B member 1
30	JAK1	Janus kinase 1
29	CREB1	cAMP responsive element binding protein 1
29	CD44	CD44 molecule (Indian blood group)
28	PLK1	polo-like kinase 1
28	HDAC9	histone deacetylase 9
27	KIT	v-kit Hardy-Zuckerman 4 feline sarcoma viral oncogene homolog
27	CXCL12	chemokine (C-X-C motif) ligand 12
27	APP	amyloid beta (A4) precursor protein
27	ACTB	actin, beta
26	MAPK11	mitogen-activated protein kinase 11
25	EZR	ezrin
25	NOS3	nitric oxide synthase 3 (endothelial cell)
24	GAPDH	glyceraldehyde-3-phosphate dehydrogenase
24	SUMO1	SMT3 suppressor of mif two 3 homolog 1 (S. cerevisiae)
24	TLR4	toll-like receptor 4
24	HLA-DRB1	major histocompatibility complex, class II, DR beta 1
23	IKBKB	inhibitor of kappa light polypeptide gene enhancer in B-cells, kinase beta
22	F2	coagulation factor II (thrombin)
21	JAK3	Janus kinase 3
21	PML	promyelocytic leukemia
20	MAPK10	mitogen-activated protein kinase 10

20	ALB	albumin
19	BIRC5	baculoviral IAP repeat containing 5
19	BCR	breakpoint cluster region
19	CDK4	cyclin-dependent kinase 4
19	PSMD4	proteasome (prosome, macropain) 26S subunit, non-ATPase, 4
19	NCAM1	neural cell adhesion molecule 1
18	PSMC5	proteasome (prosome, macropain) 26S subunit, ATPase, 5
17	INSR	insulin receptor
17	CCND3	cyclin D3
17	SMARCA4	SWI/SNF related, matrix associated, actin dependent regulator of chromatin, subfamily a, member 4
17	TRAF2	TNF receptor-associated factor 2
17	HLA-A	major histocompatibility complex, class I, A
16	PSMD13	proteasome (prosome, macropain) 26S subunit, non-ATPase, 13
16	PSMD11	proteasome (prosome, macropain) 26S subunit, non-ATPase, 11
16	PSMD9	proteasome (prosome, macropain) 26S subunit, non-ATPase, 9
16	PSMD3	proteasome (prosome, macropain) 26S subunit, non-ATPase, 3
16	ACTN1	actinin, alpha 1
16	PSMC3	proteasome (prosome, macropain) 26S subunit, ATPase, 3
16	TSPO	translocator protein (18kDa)
16	DNM2	dynamin 2
16	PSMC1	proteasome (prosome, macropain) 26S subunit, ATPase, 1
16	PSMF1	proteasome (prosome, macropain) inhibitor subunit 1 (PI31)
16	PSMD1	proteasome (prosome, macropain) 26S subunit, non-ATPase, 1
15	CLU	clusterin
15	HLA-C	major histocompatibility complex, class I, C
15	MYH14	myosin, heavy chain 14, non-muscle
15	HLA-B	major histocompatibility complex, class I, B
14	SQSTM1	sequestosome 1
14	BECN1	beclin 1, autophagy related
14	OASL	2'-5'-oligoadenylate synthetase-like
14	OAS1	2'-5'-oligoadenylate synthetase 1, 40/46kDa
13	ANGPT2	angiopoietin 2
13	TYK2	tyrosine kinase 2
13	BCL6	B-cell CLL/lymphoma 6
13	NFATC1	nuclear factor of activated T-cells, cytoplasmic, calcineurin-dependent 1
13	DECR1	2,4-dienoyl CoA reductase 1, mitochondrial
13	PARK2	parkinson protein 2, E3 ubiquitin protein ligase (parkin)
12	HSPA5	heat shock 70kDa protein 5 (glucose-regulated protein, 78kDa)
12	MSN	moesin
12	ARNT	aryl hydrocarbon receptor nuclear translocator
12	CDC37	cell division cycle 37 homolog (S. cerevisiae)
12	NTRK1	neurotrophic tyrosine kinase, receptor, type 1
12	DDX41	DEAD (Asp-Glu-Ala-Asp) box polypeptide 41
12	PRDM10	PR domain containing 10
11	CD80	CD80 molecule
11	TGFBR2	transforming growth factor, beta receptor II (70/80kDa)
11	BRAF	v-raf murine sarcoma viral oncogene homolog B1
11	CYLD	cylindromatosis (turban tumor syndrome)
10	ANXA2	annexin A2
10	SLC3A2	solute carrier family 3 (activators of dibasic and neutral amino acid transport), member 2
10	PDIA3	protein disulfide isomerase family A, member 3
10	TSC1	tuberous sclerosis 1
10	AP2M1	adaptor-related protein complex 2, mu 1 subunit
10	NCF2	neutrophil cytosolic factor 2

Supplementary Table 7. List of genes included in the SALS2-related PPI network.

Degree	Node	Gene name
326	UBC	ubiquitin C
131	AKT1	v-akt murine thymoma viral oncogene homolog 1
130	TP53	tumor protein p53
105	EGFR	epidermal growth factor receptor
99	RAC1	ras-related C3 botulinum toxin substrate 1 (rho family, small GTP binding protein Rac1)
98	STAT3	signal transducer and activator of transcription 3 (acute-phase response factor)
97	MAPK1	mitogen-activated protein kinase 1
95	HSP90AA1	heat shock protein 90kDa alpha (cytosolic), class A member 1
90	JUN	jun proto-oncogene
89	MYC	v-myc myelocytomatosis viral oncogene homolog (avian)
88	CCND1	cyclin D1
87	UBB	ubiquitin B
86	RPS27A	ribosomal protein S27a
81	HRAS	v-Ha-ras Harvey rat sarcoma viral oncogene homolog
81	UBA52	ubiquitin A-52 residue ribosomal protein fusion product 1
79	MAPK14	mitogen-activated protein kinase 14
78	RHOA	ras homolog family member A
76	CTNNB1	catenin (cadherin-associated protein), beta 1, 88kDa
76	MAPK8	mitogen-activated protein kinase 8
75	PIK3R1	phosphoinositide-3-kinase, regulatory subunit 1 (alpha)
75	MAPK3	mitogen-activated protein kinase 3
74	PTPN11	protein tyrosine phosphatase, non-receptor type 11
74	FOS	FBJ murine osteosarcoma viral oncogene homolog
72	NFKB1	nuclear factor of kappa light polypeptide gene enhancer in B-cells 1
71	GRB2	growth factor receptor-bound protein 2
70	STAT1	signal transducer and activator of transcription 1, 91kDa
69	ABL1	c-abl oncogene 1, non-receptor tyrosine kinase
69	FYN	FYN oncogene related to SRC, FGR, YES
68	RELA	v-rel reticuloendotheliosis viral oncogene homolog A (avian)
68	JAK2	Janus kinase 2
68	CREBBP	CREB binding protein
67	CDC42	cell division cycle 42 (GTP binding protein, 25kDa)
67	VEGFA	vascular endothelial growth factor A
66	EP300	E1A binding protein p300
66	SOS1	son of sevenless homolog 1 (Drosophila)
65	HSPA8	heat shock 70kDa protein 8
64	SHC1	SHC (Src homology 2 domain containing) transforming protein 1
62	AGT	angiotensinogen (serpin peptidase inhibitor, clade A, member 8)
61	PTK2	PTK2 protein tyrosine kinase 2
61	HSPB1	heat shock 27kDa protein 1
60	CREB1	cAMP responsive element binding protein 1
59	PLCG1	phospholipase C, gamma 1
57	AR	androgen receptor
56	RAC3	ras-related C3 botulinum toxin substrate 3 (rho family, small GTP binding protein Rac3)
56	CDH1	cadherin 1, type 1, E-cadherin (epithelial)
56	EGR1	early growth response 1
56	CDKN1B	cyclin-dependent kinase inhibitor 1B (p27, Kip1)
55	NRAS	neuroblastoma RAS viral (v-ras) oncogene homolog
55	PRKCA	protein kinase C, alpha
55	RAF1	v-raf-1 murine leukemia viral oncogene homolog 1
55	HIF1A	hypoxia inducible factor 1, alpha subunit (basic helix-loop-helix transcription factor)
54	APP	amyloid beta (A4) precursor protein
53	JAK1	Janus kinase 1
53	LYN	v-yes-1 Yamaguchi sarcoma viral related oncogene homolog
52	ACTB	actin, beta
52	NOTCH1	notch 1
51	BTRC	beta-transducin repeat containing E3 ubiquitin protein ligase
51	SMAD3	SMAD family member 3
51	IGF1	insulin-like growth factor 1 (somatomedin C)
51	PRKCD	protein kinase C, delta
50	SKP1	S-phase kinase-associated protein 1
50	MTOR	mechanistic target of rapamycin (serine/threonine kinase)
50	NOS3	nitric oxide synthase 3 (endothelial cell)
49	CUL1	cullin 1
48	ERBB2	v-erb-b2 erythroblastic leukemia viral oncogene homolog 2, neuro/glioblastoma derived oncogene homolog (avian)
48	KIT	v-kit Hardy-Zuckerman 4 feline sarcoma viral oncogene homolog
47	CAV1	caveolin 1, caveolae protein, 22kDa
47	PRKACA	protein kinase, cAMP-dependent, catalytic, alpha
45	IGF1R	insulin-like growth factor 1 receptor
45	PLK1	polo-like kinase 1
44	CXCL12	chemokine (C-X-C motif) ligand 12
44	HSP90AB1	heat shock protein 90kDa alpha (cytosolic), class B member 1

44	PXN	paxillin
43	NFKBIA	nuclear factor of kappa light polypeptide gene enhancer in B-cells inhibitor, alpha
43	PDGFRB	platelet-derived growth factor receptor, beta polypeptide
42	BCL2L1	BCL2-like 1
42	KRAS	v-Ki-ras2 Kirsten rat sarcoma viral oncogene homolog
41	PDGFB	platelet-derived growth factor beta polypeptide
41	SMAD4	SMAD family member 4
41	MET	met proto-oncogene (hepatocyte growth factor receptor)
40	YES1	v-yes-1 Yamaguchi sarcoma viral oncogene homolog 1
40	PSMA4	proteasome (prosome, macropain) subunit, alpha type, 4
40	PSMB1	proteasome (prosome, macropain) subunit, beta type, 1
40	PSMC2	proteasome (prosome, macropain) 26S subunit, ATPase, 2
39	RAC2	ras-related C3 botulinum toxin substrate 2 (rho family, small GTP binding protein Rac2)
39	PSMD10	proteasome (prosome, macropain) 26S subunit, non-ATPase, 10
39	PSMD14	proteasome (prosome, macropain) 26S subunit, non-ATPase, 14
39	PSMD3	proteasome (prosome, macropain) 26S subunit, non-ATPase, 3
39	MAPK11	mitogen-activated protein kinase 11
39	PSMC1	proteasome (prosome, macropain) 26S subunit, ATPase, 1
38	PSMD12	proteasome (prosome, macropain) 26S subunit, non-ATPase, 12
38	PSMD6	proteasome (prosome, macropain) 26S subunit, non-ATPase, 6
38	PSMD1	proteasome (prosome, macropain) 26S subunit, non-ATPase, 1
38	PSMF1	proteasome (prosome, macropain) inhibitor subunit 1 (PI31)
38	F2	coagulation factor II (thrombin)
38	PSMA5	proteasome (prosome, macropain) subunit, alpha type, 5
38	PSMA6	proteasome (prosome, macropain) subunit, alpha type, 6
38	SMAD2	SMAD family member 2
38	PSMA1	proteasome (prosome, macropain) subunit, alpha type, 1
38	PSMB4	proteasome (prosome, macropain) subunit, beta type, 4
38	PSMB5	proteasome (prosome, macropain) subunit, beta type, 5
38	PSMB2	proteasome (prosome, macropain) subunit, beta type, 2
38	PSMB3	proteasome (prosome, macropain) subunit, beta type, 3
38	PSMC6	proteasome (prosome, macropain) 26S subunit, ATPase, 6
37	TLR4	toll-like receptor 4
37	PTK2B	PTK2B protein tyrosine kinase 2 beta
37	PSME3	proteasome (prosome, macropain) activator subunit 3 (PA28 gamma; Ki)
37	PSMB6	proteasome (prosome, macropain) subunit, beta type, 6
37	PSMB7	proteasome (prosome, macropain) subunit, beta type, 7
36	ITGB1	integrin, beta 1 (fibronectin receptor, beta polypeptide, antigen CD29 includes MDF2, MSK12)
36	PSMD9	proteasome (prosome, macropain) 26S subunit, non-ATPase, 9
36	PSME4	proteasome (prosome, macropain) activator subunit 4
36	PRKCB	protein kinase C, beta
35	CASP3	caspase 3, apoptosis-related cysteine peptidase
35	PRKACB	protein kinase, cAMP-dependent, catalytic, beta
35	LNPEP	leucyl/cystinyl aminopeptidase
35	PPARA	peroxisome proliferator-activated receptor alpha
34	YWHAZ	tyrosine 3-monooxygenase/tryptophan 5-monooxygenase activation protein, zeta polypeptide
34	JAK3	Janus kinase 3
34	PRKCZ	protein kinase C, zeta
34	SUMO1	SMT3 suppressor of mif two 3 homolog 1 (S. cerevisiae)
33	GAPDH	glyceraldehyde-3-phosphate dehydrogenase
33	GAB2	GRB2-associated binding protein 2
33	MMP2	matrix metallopeptidase 2 (gelatinase A, 72kDa gelatinase, 72kDa type IV collagenase)
32	HDAC9	histone deacetylase 9
31	INSR	insulin receptor
31	AKT2	v-akt murine thymoma viral oncogene homolog 2
30	ARRB2	arrestin, beta 2
30	HSPA4	heat shock 70kDa protein 4
30	PLCB1	phospholipase C, beta 1 (phosphoinositide-specific)
29	B2M	beta-2-microglobulin
29	FGR	Gardner-Rasheed feline sarcoma viral (v-fgr) oncogene homolog
29	SMARCA4	SWI/SNF related, matrix associated, actin dependent regulator of chromatin, subfamily a, member 4
28	BCR	breakpoint cluster region
27	TRAF2	TNF receptor-associated factor 2
27	CEBPB	CCAAT/enhancer binding protein (C/EBP), beta
27	PML	promyelocytic leukemia
27	CDK4	cyclin-dependent kinase 4
27	MAPK9	mitogen-activated protein kinase 9
26	HLA-C	major histocompatibility complex, class I, C
26	VAV2	vav 2 guanine nucleotide exchange factor
26	PRKCE	protein kinase C, epsilon
25	A2M	alpha-2-macroglobulin
25	PDGFRA	platelet-derived growth factor receptor, alpha polypeptide
25	NCAM1	neural cell adhesion molecule 1
25	ALB	albumin

24	MYD88	myeloid differentiation primary response 88
24	KITLG	KIT ligand
24	SH3GL2	SH3-domain GRB2-like 2
24	PLD2	phospholipase D2
24	BAX	BCL2-associated X protein
24	DNM2	dynamin 2
24	MAP3K5	mitogen-activated protein kinase kinase kinase 5
23	HMGB1	high mobility group box 1
23	HSPD1	heat shock 60kDa protein 1 (chaperonin)
23	PSMA2	proteasome (prosome, macropain) subunit, alpha type, 2
23	PTAFR	platelet-activating factor receptor
23	RET	ret proto-oncogene
23	ABI1	abl-interactor 1
22	RPS6KA3	ribosomal protein S6 kinase, 90kDa, polypeptide 3
22	ITCH	itchy E3 ubiquitin protein ligase
22	CAMK2D	calcium/calmodulin-dependent protein kinase II delta
21	IGF2	insulin-like growth factor 2 (somatomedin A)
21	SPTBN2	spectrin, beta, non-erythrocytic 2
21	TYK2	tyrosine kinase 2
21	ARHGEF7	Rho guanine nucleotide exchange factor (GEF) 7
21	IFNB1	interferon, beta 1, fibroblast
21	CAMK2G	calcium/calmodulin-dependent protein kinase II gamma
21	SNCA	synuclein, alpha (non A4 component of amyloid precursor)
21	DECR1	2,4-dienoyl CoA reductase 1, mitochondrial
21	HSPA5	heat shock 70kDa protein 5 (glucose-regulated protein, 78kDa)
21	MAPK13	mitogen-activated protein kinase 13
21	HSP90B1	heat shock protein 90kDa beta (Grp94), member 1
20	MAP2K4	mitogen-activated protein kinase kinase 4
20	MITF	microphthalmia-associated transcription factor
20	SOS2	son of sevenless homolog 2 (Drosophila)
20	ADRM1	adhesion regulating molecule 1
20	CALM1	calmodulin 1 (phosphorylase kinase, delta)
20	CD74	CD74 molecule, major histocompatibility complex, class II invariant chain
20	FGFR2	fibroblast growth factor receptor 2
20	PSEN1	presenilin 1
20	TSPO	translocator protein (18kDa)
20	DCTN1	dynactin 1
20	CSF1R	colony stimulating factor 1 receptor
20	SUMO2	SMT3 suppressor of mif two 3 homolog 2 (S. cerevisiae)
19	RPS6	ribosomal protein S6
19	NFE2L2	nuclear factor (erythroid-derived 2)-like 2
19	APOA1	apolipoprotein A-I
19	IFIT1	interferon-induced protein with tetratricopeptide repeats 1
19	RPS6KA5	ribosomal protein S6 kinase, 90kDa, polypeptide 5
19	CANX	calnexin
19	RIPK1	receptor (TNFRSF)-interacting serine-threonine kinase 1
19	ZAP70	zeta-chain (TCR) associated protein kinase 70kDa
19	GNAS	GNAS complex locus
19	MYH14	myosin, heavy chain 14, non-muscle
19	OAS1	2'-5'-oligoadenylate synthetase 1, 40/46kDa
19	DYNLL1	dynein, light chain, LC8-type 1
19	NCF1	neutrophil cytosolic factor 1
19	UBE2D2	ubiquitin-conjugating enzyme E2D 2
19	WWP1	WW domain containing E3 ubiquitin protein ligase 1
18	ACTR3	ARP3 actin-related protein 3 homolog (yeast)
18	ITGA6	integrin, alpha 6
18	SIN3A	SIN3 transcription regulator homolog A (yeast)
18	SDC4	syndecan 4
18	CALR	calreticulin
18	ACTR1A	ARP1 actin-related protein 1 homolog A, centractin alpha (yeast)
18	PARP1	poly (ADP-ribose) polymerase 1
18	OASL	2'-5'-oligoadenylate synthetase-like
18	TGFBR2	transforming growth factor, beta receptor II (70/80kDa)
18	OAS3	2'-5'-oligoadenylate synthetase 3, 100kDa
18	PARK2	parkinson protein 2, E3 ubiquitin protein ligase (parkin)
18	MAPK7	mitogen-activated protein kinase 7
18	BRAF	v-raf murine sarcoma viral oncogene homolog B1
18	PAFAH1B1	platelet-activating factor acetylhydrolase 1b, regulatory subunit 1 (45kDa)
18	STK11	serine/threonine kinase 11
17	ARPC5	actin related protein 2/3 complex, subunit 5, 16kDa
17	ARPC2	actin related protein 2/3 complex, subunit 2, 34kDa
17	ARPC3	actin related protein 2/3 complex, subunit 3, 21kDa
17	ITPR1	inositol 1,4,5-trisphosphate receptor, type 1
17	BECN1	beclin 1, autophagy related

17	TRAF3	TNF receptor-associated factor 3
17	EPAS1	endothelial PAS domain protein 1
17	PSEN2	presenilin 2 (Alzheimer disease 4)
17	DCTN2	dynactin 2 (p50)
17	CYFIP2	cytoplasmic FMR1 interacting protein 2
17	NCKAP1	NCK-associated protein 1
17	HNRNPK	heterogeneous nuclear ribonucleoprotein K
16	RNF41	ring finger protein 41
16	SOX9	SRY (sex determining region Y)-box 9
16	KLK3	kallikrein-related peptidase 3
16	ATM	ataxia telangiectasia mutated
16	DYNC1I2	dynein, cytoplasmic 1, intermediate chain 2
16	GBP2	guanylate binding protein 2, interferon-inducible
16	TFRC	transferrin receptor (p90, CD71)
16	DCTN3	dynactin 3 (p22)
16	APOE	apolipoprotein E
16	RARA	retinoic acid receptor, alpha
15	NME1	NME/NM23 nucleoside diphosphate kinase 1
15	RPA1	replication protein A1, 70kDa
15	FBXW11	F-box and WD repeat domain containing 11
15	ADAR	adenosine deaminase, RNA-specific
15	F11R	F11 receptor
15	NCSTN	nicastrin
15	ARPC1A	actin related protein 2/3 complex, subunit 1A, 41kDa
15	SPP1	secreted phosphoprotein 1
15	IFNGR1	interferon gamma receptor 1
14	DNAJA3	DnaJ (Hsp40) homolog, subfamily A, member 3
14	XRCC6	X-ray repair complementing defective repair in Chinese hamster cells 6
14	ID2	inhibitor of DNA binding 2, dominant negative helix-loop-helix protein
14	DLG1	discs, large homolog 1 (Drosophila)
14	UBE2N	ubiquitin-conjugating enzyme E2N
14	PRDM10	PR domain containing 10
14	RBPJ	recombination signal binding protein for immunoglobulin kappa J region
14	HSPH1	heat shock 105kDa/110kDa protein 1
14	AP1S1	adaptor-related protein complex 1, sigma 1 subunit
14	ARF1	ADP-ribosylation factor 1
13	CDK6	cyclin-dependent kinase 6
13	RPL13A	ribosomal protein L13a
13	BSG	basigin (Ok blood group)
13	AP2A2	adaptor-related protein complex 2, alpha 2 subunit
13	SEC13	SEC13 homolog (S. cerevisiae)
13	FAU	Finkel-Biskis-Reilly murine sarcoma virus (FBR-MuSV) ubiquitously expressed
13	PDIA3	protein disulfide isomerase family A, member 3
13	CYBA	cytochrome b-245, alpha polypeptide
13	FES	feline sarcoma oncogene
13	DCTN6	dynactin 6
13	DCTN4	dynactin 4 (p62)
13	CNR1	cannabinoid receptor 1 (brain)
13	PRKAR1A	protein kinase, cAMP-dependent, regulatory, type I, alpha
13	CAT	catalase
13	HIPK2	homeodomain interacting protein kinase 2
13	MBP	myelin basic protein
12	OSBPL1A	oxysterol binding protein-like 1A
12	ITGA3	integrin, alpha 3 (antigen CD49C, alpha 3 subunit of VLA-3 receptor)
12	NF1	neurofibromin 1
12	RILP	Rab interacting lysosomal protein
12	IFITM1	interferon induced transmembrane protein 1
12	TF	transferrin
12	NPY	neuropeptide Y
12	LMO4	LIM domain only 4
12	CACNA1C	calcium channel, voltage-dependent, L type, alpha 1C subunit
12	DDX58	DEAD (Asp-Glu-Ala-Asp) box polypeptide 58
12	DDX41	DEAD (Asp-Glu-Ala-Asp) box polypeptide 41
12	GATA2	GATA binding protein 2
12	DYNC2LI1	dynein, cytoplasmic 2, light intermediate chain 1
12	PIP5K1C	phosphatidylinositol-4-phosphate 5-kinase, type I, gamma
12	RPL22	ribosomal protein L22
12	DYNC1LI2	dynein, cytoplasmic 1, light intermediate chain 2
12	PRDX1	peroxiredoxin 1
12	IP6K2	inositol hexakisphosphate kinase 2
11	CXCL11	chemokine (C-X-C motif) ligand 11
11	TNFRSF21	tumor necrosis factor receptor superfamily, member 21
11	HDAC5	histone deacetylase 5
11	APLN	apelin

11	RPS14	ribosomal protein S14
11	RPS19	ribosomal protein S19
11	GNRH1	gonadotropin-releasing hormone 1 (luteinizing-releasing hormone)
11	RPS24	ribosomal protein S24
11	IFI6	interferon, alpha-inducible protein 6
11	ELMO1	engulfment and cell motility 1
11	EFNB2	ephrin-B2
11	FGF10	fibroblast growth factor 10
11	SLC3A2	solute carrier family 3 (activators of dibasic and neutral amino acid transport), member 2
11	MX2	myxovirus (influenza virus) resistance 2 (mouse)
11	SOD2	superoxide dismutase 2, mitochondrial
11	SAR1B	SAR1 homolog B (S. cerevisiae)
11	EPRS	glutamyl-prolyl-tRNA synthetase
11	PLA2G6	phospholipase A2, group VI (cytosolic, calcium-independent)
11	JAG1	jagged 1
11	DUSP3	dual specificity phosphatase 3
11	CLTA	clathrin, light chain A
11	UBE2D3	ubiquitin-conjugating enzyme E2D 3
11	TICAM1	toll-like receptor adaptor molecule 1
10	OGT	O-linked N-acetylglucosamine (GlcNAc) transferase
10	AP1G1	adaptor-related protein complex 1, gamma 1 subunit
10	UBQLN1	ubiquilin 1
10	RPS17	ribosomal protein S17
10	FTH1	ferritin, heavy polypeptide 1
10	SEC31A	SEC31 homolog A (S. cerevisiae)
10	GAL	galanin/GMAP prepropeptide
10	SCARB1	scavenger receptor class B, member 1
10	THPO	thrombopoietin
10	UBE2V1	ubiquitin-conjugating enzyme E2 variant 1
10	EPS8	epidermal growth factor receptor pathway substrate 8
10	AP1S2	adaptor-related protein complex 1, sigma 2 subunit
10	RICTOR	RPTOR independent companion of MTOR, complex 2
10	ADORA3	adenosine A3 receptor

Supplementary Table 8. Pathway enrichment analyses on the list of statistically deregulated neuroinflammatory genes in SALS patients.

#	Maps	min(pValue)	Min FDR	SALS1/CTRL+		SALS2_CTRL	
				p-value	FDR	p-value	FDR
1	Immune response_Classical complement pathway	1.384E-17	1.082E-14	1.384E-17	1.082E-14	1.090E-02	1.752E-02
2	Immune response_Role of PKR in stress-induced antiviral cell response	8.973E-17	8.318E-15	5.572E-04	4.685E-03	8.973E-17	8.318E-15
3	Immune response_Lectin induced complement pathway	9.264E-17	3.622E-14	9.264E-17	3.622E-14	2.192E-03	4.080E-03
4	Immune response_HMGB1/RAGE signaling pathway	1.427E-15	1.203E-13	3.541E-04	3.505E-03	1.427E-15	1.203E-13
5	SLE genetic marker-specific pathways in T cells	2.990E-15	2.133E-13	4.328E-09	2.821E-07	2.990E-15	2.133E-13
6	Oxidative stress_Activation of NADPH oxidase	3.036E-15	2.133E-13	1.659E-02	5.253E-02	3.036E-15	2.133E-13
7	PDE4 regulation of cyto/chemokine expression in inflammatory skin diseases	3.221E-15	2.133E-13	3.743E-02	9.413E-02	3.221E-15	2.133E-13
8	Immune response_B cell antigen receptor (BCR) pathway	8.304E-15	5.132E-13	1.886E-03	1.109E-02	8.304E-15	5.132E-13
9	Development_VEGF signaling via VEGFR2 - generic cascades	2.129E-14	1.234E-12	2.646E-04	2.914E-03	2.129E-14	1.234E-12
10	Immune response_Antigen presentation by MHC class I, classical pathway	2.835E-14	1.546E-12	3.982E-04	3.731E-03	2.835E-14	1.546E-12
11	Immune response_Platelet activating factor/ PTAFR pathway signaling	4.701E-14	2.293E-12	2.570E-03	1.305E-02	4.701E-14	2.293E-12
12	Immune response_T cell receptor signaling pathway	4.701E-14	2.293E-12	2.570E-03	1.305E-02	4.701E-14	2.293E-12
13	Development_EGFR signaling pathway	7.271E-14	3.210E-12	9.080E-03	3.395E-02	7.271E-14	3.210E-12
14	Role of Tissue factor in cancer independent of coagulation protease signaling	1.408E-13	5.933E-12	1.757E-03	1.049E-02	1.408E-13	5.933E-12
15	Aberrant production of IL-2 and IL-17 in SLE T cells	1.978E-13	7.973E-12	3.369E-03	1.597E-02	1.978E-13	7.973E-12
16	Immune response_CD16 signaling in NK cells	2.733E-13	1.056E-11	8.445E-06	2.608E-04	2.733E-13	1.056E-11
17	Development_c-Kit ligand signaling pathway during hemopoiesis	7.479E-13	2.773E-11	1.414E-04	1.962E-03	7.479E-13	2.773E-11
18	Immune response_IL-3 signaling via ERK and PI3K	1.157E-12	4.117E-11	2.540E-04	2.878E-03	1.157E-12	4.117E-11
19	Development_Cytokine-mediated regulation of megakaryopoiesis	1.199E-12	4.117E-11	5.572E-04	4.685E-03	1.199E-12	4.117E-11
20	Immune response_ICOS signaling pathway in T-helper cell	1.722E-12	5.700E-11	2.704E-05	6.219E-04	1.722E-12	5.700E-11
21	Signal transduction_Additional pathways of NF-kB activation (in the cytoplasm)	1.873E-12	5.986E-11	1.507E-01	2.567E-01	1.873E-12	5.986E-11
22	Immune response_CCL2 signaling	2.954E-12	8.557E-11	4.758E-02	1.130E-01	2.954E-12	8.557E-11
23	Immune response_Role of DAP12 receptors in NK cells	4.601E-12	1.292E-10	8.672E-06	2.608E-04	4.601E-12	1.292E-10
24	Immune response_TLR2 and TLR4 signaling pathways	1.078E-11	2.848E-10	5.609E-02	1.286E-01	1.078E-11	2.848E-10
25	Immune response_IL-15 signaling	1.415E-11	3.451E-10	7.538E-02	1.583E-01	1.415E-11	3.451E-10
26	Cell adhesion_Chemokines and adhesion	2.272E-11	5.137E-10	3.726E-09	2.649E-07	2.272E-11	5.137E-10
27	SLE genetic marker-specific pathways in B cells	2.272E-11	5.137E-10	2.034E-07	1.060E-05	2.272E-11	5.137E-10
28	Immune response_IL-4 signaling pathway	2.480E-11	5.473E-10	9.783E-03	3.579E-02	2.480E-11	5.473E-10
29	Immune response_HSP60 and HSP70/ TLR signaling pathway	2.692E-11	5.802E-10	3.982E-04	3.731E-03	2.692E-11	5.802E-10
30	Immune response_IL-18 signaling	3.571E-11	6.962E-10	3.996E-03	1.838E-02	3.571E-11	6.962E-10
31	Apoptosis and survival_NGF/ TrkA PI3K-mediated signaling	3.605E-11	6.962E-10	1.353E-04	1.923E-03	3.605E-11	6.962E-10
32	Immune response_IL-5 signaling via PI3K, MAPK and NF-kB	3.605E-11	6.962E-10	7.128E-04	5.630E-03	3.605E-11	6.962E-10
33	Cytoskeleton remodeling_Cytoskeleton remodeling	3.888E-11	7.356E-10	9.801E-06	2.839E-04	3.888E-11	7.356E-10
34	Development_Non-genomic action of Retinoic acid in cell differentiation	4.070E-11	7.397E-10	1.629E-01	2.733E-01	4.070E-11	7.397E-10
35	Immune response_Fc epsilon RI pathway	4.070E-11	7.397E-10	4.465E-04	4.013E-03	4.070E-11	7.397E-10
36	Development_Adenosine A2B receptor signaling	4.361E-11	7.774E-10	3.743E-02	9.413E-02	4.361E-11	7.774E-10
37	Immune response_IL-6 signaling pathway via JAK/STAT	4.751E-11	8.310E-10	4.521E-04	4.018E-03	4.751E-11	8.310E-10
38	Development_PDGF signaling via STATs and NF-kB	6.419E-11	1.102E-09	8.312E-03	3.155E-02	6.419E-11	1.102E-09
39	Cytoskeleton remodeling_TGF, WNT and cytoskeletal remodeling	7.224E-11	1.218E-09	1.759E-08	9.824E-07	7.224E-11	1.218E-09
40	Signal transduction_mTORC2 downstream signaling	8.608E-11	1.425E-09	1.613E-03	9.855E-03	8.608E-11	1.425E-09

41	Cytoskeleton remodeling_FAK signaling	9.008E-11	1.448E-09	1.445E-02	4.709E-02	9.008E-11	1.448E-09
42	Immune response_IL-6 signaling pathway via MEK/ERK and PI3K/AKT cascades	9.150E-11	1.448E-09	2.633E-03	1.329E-02	9.150E-11	1.448E-09
43	Main pathways of Schwann cells transformation in neurofibromatosis type 1	9.214E-11	1.448E-09	9.199E-04	6.661E-03	9.214E-11	1.448E-09
44	Oxidative stress_Activation of NOX1, NOX5, DUOX1 and DUOX2 NADPH Oxidases	1.043E-10	1.611E-09	8.987E-02	1.809E-01	1.043E-10	1.611E-09
45	Immune response_Role of DPP4 (CD26) in immune regulation	1.078E-10	1.638E-09	6.491E-03	2.551E-02	1.078E-10	1.638E-09
46	LRRK2 in neurons in Parkinson's disease	1.165E-10	1.715E-09	5.140E-09	3.092E-07	1.165E-10	1.715E-09
47	Normal and pathological TGF-beta-mediated regulation of cell proliferation	1.165E-10	1.715E-09	1.564E-04	2.073E-03	1.165E-10	1.715E-09
48	Mucin expression in CF airways	1.207E-10	1.748E-09	2.548E-01	3.731E-01	1.207E-10	1.748E-09
49	Development_Prolactin receptor signaling	1.320E-10	1.882E-09	3.369E-03	1.597E-02	1.320E-10	1.882E-09
50	Apoptosis and survival_Role of PKR in stress-induced apoptosis	1.530E-10	2.141E-09	2.123E-03	1.145E-02	1.530E-10	2.141E-09
51	Myeloid-derived suppressor cells and M2 macrophages in cancer	1.547E-10	2.141E-09	8.077E-01	8.289E-01	1.547E-10	2.141E-09
52	Immune response_IL-2 activation and signaling pathway	1.668E-10	2.273E-09	1.261E-03	8.217E-03	1.668E-10	2.273E-09
53	Development_FGFR signaling pathway	2.271E-10	3.051E-09	4.758E-02	1.130E-01	2.271E-10	3.051E-09
54	Immune response _CCR3 signaling in eosinophils	2.332E-10	3.089E-09	1.313E-01	2.304E-01	2.332E-10	3.089E-09
55	Role of tumor microenvironment in plexiform neurofibroma formation in neurofibromatosis type 1	2.506E-10	3.234E-09	2.871E-03	1.406E-02	2.506E-10	3.234E-09
56	PDE4 regulation of cyto/chemokine expression in arthritis	2.546E-10	3.234E-09	1.272E-01	2.272E-01	2.546E-10	3.234E-09
57	Transcription_CREB signaling pathway	2.546E-10	3.234E-09	3.511E-02	9.033E-02	2.546E-10	3.234E-09
58	Reproduction_Gonadotropin-releasing hormone (GnRH) signaling	3.189E-10	3.995E-09	3.567E-02	9.136E-02	3.189E-10	3.995E-09
59	Immune response_Alternative complement pathway	4.558E-10	3.960E-08	4.558E-10	3.960E-08	5.656E-05	1.573E-04
60	Immune response_HMGB1/TLR signaling pathway	5.971E-10	7.345E-09	6.231E-02	1.380E-01	5.971E-10	7.345E-09
61	Immune response_IL-3 signaling via JAK/STAT, p38, JNK and NF-kB	6.022E-10	7.345E-09	3.701E-06	1.357E-04	6.022E-10	7.345E-09
62	Immune response_Naive CD4+ T cell differentiation	6.354E-10	7.649E-09	1.004E-03	6.827E-03	6.354E-10	7.649E-09
63	Main growth factor signaling cascades in multiple myeloma cells	6.511E-10	7.738E-09	3.586E-03	1.669E-02	6.511E-10	7.738E-09
64	Development_PDGF signaling via MAPK cascades	9.628E-10	1.130E-08	1.127E-03	7.597E-03	9.628E-10	1.130E-08
65	Immune response_Inhibitory PD-1 signaling in T cells	1.228E-09	1.424E-08	2.123E-03	1.145E-02	1.228E-09	1.424E-08
66	Immune response_TREM1 signaling pathway	1.410E-09	1.609E-08	1.659E-02	5.253E-02	1.410E-09	1.609E-08
67	Immune response_Histamine H1 receptor signaling in immune response	1.440E-09	1.609E-08	1.216E-01	2.208E-01	1.440E-09	1.609E-08
68	IL-17-induced mucin expression in CF airways	1.440E-09	1.609E-08	3.288E-02	8.655E-02	1.440E-09	1.609E-08
69	Inhibition of neutrophil migration by proresolving lipid mediators in COPD	1.493E-09	1.648E-08	1.508E-06	6.937E-05	1.493E-09	1.648E-08
70	Cytoskeleton remodeling_Hyaluronic acid/ CD44 signaling pathways	1.582E-09	1.675E-08	1.033E-05	2.885E-04	1.582E-09	1.675E-08
71	Signal transduction_PTMs in BAFF-induced canonical NF-kB signaling	1.582E-09	1.675E-08	2.979E-01	4.182E-01	1.582E-09	1.675E-08
72	Immune response_Role of integrins in NK cells cytotoxicity	1.590E-09	1.675E-08	3.505E-04	3.505E-03	1.590E-09	1.675E-08
73	Role of Tissue factor-induced Thrombin signaling in cancerogenesis	2.050E-09	2.112E-08	6.395E-03	2.551E-02	2.050E-09	2.112E-08
74	Development_PEDF signaling	2.129E-09	2.169E-08	3.539E-01	4.692E-01	2.129E-09	2.169E-08
75	Neurogenesis_NGF/ TrkA MAPK-mediated signaling	2.167E-09	2.183E-08	1.724E-02	5.435E-02	2.167E-09	2.183E-08
76	Chemotaxis_CXCR4 signaling pathway	2.357E-09	2.349E-08	1.538E-03	9.620E-03	2.357E-09	2.349E-08
77	Immune response_IL-1 signaling pathway	2.411E-09	2.353E-08	1.000E-01	1.936E-01	2.411E-09	2.353E-08
78	Apoptosis and survival_Inhibition of ROS-induced apoptosis by 17beta-estradiol	2.411E-09	2.353E-08	3.073E-01	4.245E-01	2.411E-09	2.353E-08
79	Signal transduction_PTMs (ubiquitination and phosphorylation) in TNF-alpha-induced NF-kB signaling	2.521E-09	2.434E-08	7.553E-02	1.583E-01	2.521E-09	2.434E-08
80	Rheumatoid arthritis (general schema)	3.111E-09	2.973E-08	3.817E-06	1.357E-04	3.111E-09	2.973E-08
81	Signal transduction_Additional pathways of NF-kB activation (in the nucleus)	3.274E-09	3.097E-08	3.953E-02	9.814E-02	3.274E-09	3.097E-08
82	Immune response_CD28 signaling	3.582E-09	3.354E-08	7.610E-05	1.322E-03	3.582E-09	3.354E-08
83	Development_Gastrin in cell growth and proliferation	3.795E-09	3.518E-08	2.018E-02	6.093E-02	3.795E-09	3.518E-08

84	Signal transduction_NF-kB activation pathways	4.495E-09	4.126E-08	9.161E-03	3.395E-02	4.495E-09	4.126E-08
85	Signal transduction_PTMs in IL-23 signaling pathway	5.587E-09	5.077E-08	1.850E-01	2.996E-01	5.587E-09	5.077E-08
86	Signal transduction_PTMs (phosphorylation) in TNF-alpha-induced NF-kB signaling	6.029E-09	5.374E-08	1.961E-02	5.947E-02	6.029E-09	5.374E-08
87	Signal transduction_PTMs (phosphorylation and acetylation) in TNF-alpha-induced NF-kB signaling	6.029E-09	5.374E-08	3.586E-03	1.669E-02	6.029E-09	5.374E-08
88	Development_EPO-induced Jak-STAT pathway	6.187E-09	5.411E-08	2.317E-01	3.478E-01	6.187E-09	5.411E-08
89	HBV signaling via protein kinases leading to HCC	6.187E-09	5.411E-08	1.258E-02	4.206E-02	6.187E-09	5.411E-08
90	Signal transduction_Activation of PKC via G-Protein coupled receptor	6.429E-09	5.570E-08	4.233E-02	1.035E-01	6.429E-09	5.570E-08
91	Immune response_CCR5 signaling in macrophages and T lymphocytes	6.985E-09	5.887E-08	1.550E-02	4.967E-02	6.985E-09	5.887E-08
92	Immune response_IL-33 signaling pathway	6.985E-09	5.887E-08	5.909E-02	1.328E-01	6.985E-09	5.887E-08
93	Development_TGF-beta-dependent induction of EMT via MAPK	7.877E-09	6.578E-08	2.052E-05	5.349E-04	7.877E-09	6.578E-08
94	Immune response_MIF-mediated glucocorticoid regulation	8.903E-09	7.369E-08	1.962E-02	5.947E-02	8.903E-09	7.369E-08
95	SLE genetic marker-specific pathways in antigen-presenting cells (APC)	9.196E-09	7.544E-08	4.910E-05	9.142E-04	9.196E-09	7.544E-08
96	Muscle contraction_Relaxin signaling pathway	1.141E-08	9.115E-08	3.447E-01	4.631E-01	1.141E-08	9.115E-08
97	Stimulation of TGF-beta signaling in lung cancer	1.141E-08	9.115E-08	3.288E-02	8.655E-02	1.141E-08	9.115E-08
98	Oxidative stress_Role of IL-8 signaling pathway in respiratory burst	1.141E-08	9.115E-08	3.447E-01	4.631E-01	1.141E-08	9.115E-08
99	Development_VEGF signaling and activation	1.357E-08	1.075E-07	9.489E-02	1.879E-01	1.357E-08	1.075E-07
100	Signal transduction_IP3 signaling	1.633E-08	1.283E-07	3.539E-01	4.692E-01	1.633E-08	1.283E-07
101	Development_Flt3 signaling	1.995E-08	1.554E-07	4.887E-03	2.123E-02	1.995E-08	1.554E-07
102	Immune response_Th17, Th22 and Th9 cell differentiation	2.288E-08	1.724E-07	4.056E-04	3.731E-03	2.288E-08	1.724E-07
103	HIV-1 signaling via CCR5 in macrophages and T lymphocytes	2.288E-08	1.724E-07	7.553E-02	1.583E-01	2.288E-08	1.724E-07
104	Immune response_HMGB1 release from the cell	2.288E-08	1.724E-07	4.781E-05	9.142E-04	2.288E-08	1.724E-07
105	Development_Thromboxane A2 signaling pathway	2.315E-08	1.730E-07	1.565E-03	9.638E-03	2.315E-08	1.730E-07
106	Cell adhesion_Alpha-4 integrins in cell migration and adhesion	2.403E-08	1.776E-07	1.031E-02	3.647E-02	2.403E-08	1.776E-07
107	Immune response_IL-10 signaling pathway	2.413E-08	1.776E-07	4.705E-03	2.090E-02	2.413E-08	1.776E-07
108	Immune response _IFN gamma signaling pathway	2.446E-08	1.785E-07	5.316E-02	1.248E-01	2.446E-08	1.785E-07
109	Immune response_MIF - the neuroendocrine-macrophage connector	2.895E-08	2.097E-07	1.053E-01	2.022E-01	2.895E-08	2.097E-07
110	Immune response_Function of MEF2 in T lymphocytes	3.247E-08	2.333E-07	1.388E-01	2.412E-01	3.247E-08	2.333E-07
111	Immune response_MIF in innate immunity response	3.424E-08	2.442E-07	3.214E-03	1.561E-02	3.424E-08	2.442E-07
112	Development_Osteopontin signaling in osteoclasts	3.662E-08	2.591E-07	8.554E-04	6.251E-03	3.662E-08	2.591E-07
113	Role of growth factor receptors transactivation by Hyaluronic acid / CD44 signaling in tumor progression	3.747E-08	2.632E-07	2.280E-05	5.571E-04	3.747E-08	2.632E-07
114	Immune response_MIF-induced cell adhesion, migration and angiogenesis	4.153E-08	2.873E-07	1.430E-04	1.962E-03	4.153E-08	2.873E-07
115	Development_Thrombopoietin-regulated cell processes	4.153E-08	2.873E-07	2.867E-02	7.866E-02	4.153E-08	2.873E-07
116	Development_IGF-1 receptor signaling	4.512E-08	3.045E-07	9.931E-03	3.579E-02	4.512E-08	3.045E-07
117	Development_FGF-family signaling	4.512E-08	3.045E-07	4.233E-02	1.035E-01	4.512E-08	3.045E-07
118	G-protein signaling_Proinsulin C-peptide signaling	4.512E-08	3.045E-07	9.931E-03	3.579E-02	4.512E-08	3.045E-07
119	Effect of H. pylori infection on gastric epithelial cell proliferation	4.533E-08	3.045E-07	5.909E-02	1.328E-01	4.533E-08	3.045E-07
120	Development_VEGF-family signaling	5.052E-08	3.369E-07	2.790E-01	4.003E-01	5.052E-08	3.369E-07
121	PI3K Signal Transduction Pathway_Simona	5.567E-08	3.686E-07	5.450E-03	2.316E-02	5.567E-08	3.686E-07
122	Development_Adenosine A1 receptor signaling	6.213E-08	4.056E-07	1.074E-02	3.734E-02	6.213E-08	4.056E-07
123	Development_Endothelin-1/EDNRA signaling	6.213E-08	4.056E-07	2.123E-03	1.145E-02	6.213E-08	4.056E-07
124	Apoptosis and survival_BAD phosphorylation	7.353E-08	4.766E-07	2.126E-02	6.321E-02	7.353E-08	4.766E-07
125	IGF family signaling in colorectal cancer	8.150E-08	5.246E-07	6.536E-02	1.444E-01	8.150E-08	5.246E-07
126	Immune response_TLR5, TLR7, TLR8 and TLR9 signaling pathways	8.264E-08	5.283E-07	3.288E-02	8.655E-02	8.264E-08	5.283E-07

127	Th17 cells in CF	8.481E-08	5.385E-07	2.338E-03	1.244E-02	8.481E-08	5.385E-07
128	Immune response_Role of HMGB1 in dendritic cell maturation and migration	8.796E-08	5.547E-07	5.156E-04	4.480E-03	8.796E-08	5.547E-07
129	B cell signaling in hematological malignancies	9.054E-08	5.671E-07	1.820E-04	2.259E-03	9.054E-08	5.671E-07
130	Signal transduction_PTMs in IL-17-induced CIKS-dependent MAPK signaling pathways	9.306E-08	5.789E-07	4.656E-02	1.117E-01	9.306E-08	5.789E-07
131	Role of cell adhesion in vaso-occlusion in Sickle cell disease	1.056E-07	6.486E-07	6.963E-04	5.556E-03	1.056E-07	6.486E-07
132	Tissue Factor signaling in cancer via PAR1 and PAR2	1.148E-07	6.911E-07	3.539E-01	4.692E-01	1.148E-07	6.911E-07
133	Immune response_Bacterial infections in normal airways	1.148E-07	6.911E-07	3.511E-02	9.033E-02	1.148E-07	6.911E-07
134	Blood coagulation_GPVI-dependent platelet activation	1.148E-07	6.911E-07	2.570E-03	1.305E-02	1.148E-07	6.911E-07
135	Glucocorticoid-induced elevation of intraocular pressure as glaucoma risk factor	1.425E-07	8.457E-07	4.705E-03	2.090E-02	1.425E-07	8.457E-07
136	Development_EGFR signaling via small GTPases	1.437E-07	8.457E-07	1.564E-04	2.073E-03	1.437E-07	8.457E-07
137	Immune response_Innate immune response to RNA viral infection	1.441E-07	8.457E-07	3.309E-02	8.655E-02	1.441E-07	8.457E-07
138	Development_Angiotensin signaling via PYK2	1.500E-07	8.635E-07	4.887E-03	2.123E-02	1.500E-07	8.635E-07
139	Nociception_Expression and role of Nociceptin in immune system	1.500E-07	8.635E-07	3.073E-01	4.245E-01	1.500E-07	8.635E-07
140	Influence of smoking on activation of EGFR signaling in lung cancer cells	1.500E-07	8.635E-07	1.000E-01	1.936E-01	1.500E-07	8.635E-07
141	Development_GM-CSF signaling	1.579E-07	8.980E-07	8.433E-03	3.186E-02	1.579E-07	8.980E-07
142	Immune response_C5a signaling	1.579E-07	8.980E-07	3.743E-02	9.413E-02	1.579E-07	8.980E-07
143	Immune response_Gastrin in inflammatory response	1.629E-07	9.209E-07	3.043E-02	8.259E-02	1.629E-07	9.209E-07
144	Apoptosis and survival_APRIL and BAFF signaling	1.869E-07	1.050E-06	4.056E-04	3.731E-03	1.869E-07	1.050E-06
145	Immune response_IFN alpha/beta signaling pathway	2.087E-07	1.161E-06	2.889E-04	3.012E-03	2.087E-07	1.161E-06
146	Immune response_TNF-R2 signaling pathways	2.105E-07	1.161E-06	5.386E-03	2.302E-02	2.105E-07	1.161E-06
147	Development_EPO-induced MAPK pathway	2.105E-07	1.161E-06	1.239E-04	1.794E-03	2.105E-07	1.161E-06
148	Signal transduction_PTMs in BAFF-induced signaling	2.153E-07	1.174E-06	1.388E-01	2.412E-01	2.153E-07	1.174E-06
149	Immune response_NFAT in immune response	2.153E-07	1.174E-06	3.806E-05	8.038E-04	2.153E-07	1.174E-06
150	Pacap Igf1 BS	2.231E-07	1.210E-06	1.387E-03	8.892E-03	2.231E-07	1.210E-06
151	Role of B cells in SLE	2.719E-07	1.466E-06	9.829E-05	1.580E-03	2.719E-07	1.466E-06
152	Signal transduction_PTMs in IL-17-induced CIKS-independent signaling pathways	2.922E-07	1.566E-06	5.920E-03	2.418E-02	2.922E-07	1.566E-06
153	Development_Role of IL-8 in angiogenesis	3.139E-07	1.644E-06	2.276E-01	3.456E-01	3.139E-07	1.644E-06
154	Muscle contraction_Regulation of eNOS activity in endothelial cells	3.139E-07	1.644E-06	5.936E-03	2.418E-02	3.139E-07	1.644E-06
155	Ovarian cancer (main signaling cascades)	3.139E-07	1.644E-06	2.221E-04	2.592E-03	3.139E-07	1.644E-06
156	NF-AT signaling in cardiac hypertrophy	3.139E-07	1.644E-06	8.246E-02	1.703E-01	3.139E-07	1.644E-06
157	Immune response_Oncostatin M signaling via MAPK in mouse cells	3.244E-07	1.689E-06	5.817E-02	1.315E-01	3.244E-07	1.689E-06
158	Immune response_T cell subsets: secreted signals	3.491E-07	1.808E-06	2.455E-02	7.008E-02	3.491E-07	1.808E-06
159	Development_Thyroliberin signaling	4.664E-07	2.350E-06	4.520E-01	5.584E-01	4.664E-07	2.350E-06
160	Apoptosis and survival_TNF-alpha-induced ROS-dependent Caspase-3 signaling	4.664E-07	2.350E-06	1.774E-02	5.570E-02	4.664E-07	2.350E-06
161	Immune response_Regulation of T cell function by CTLA-4	4.753E-07	2.369E-06	1.999E-03	1.141E-02	4.753E-07	2.369E-06
162	G-protein signaling_RAC1 in cellular process	4.753E-07	2.369E-06	1.258E-02	4.206E-02	4.753E-07	2.369E-06
163	CXCL12_cancer_2	4.862E-07	2.410E-06	4.172E-01	5.271E-01	4.862E-07	2.410E-06
164	Membrane-bound ESR1: interaction with G-proteins signaling	5.182E-07	2.528E-06	3.994E-01	5.129E-01	5.182E-07	2.528E-06
165	Neurophysiological process_Dynein-dynactin motor complex in axonal transport in neurons	5.182E-07	2.528E-06	3.982E-04	3.731E-03	5.182E-07	2.528E-06
166	Role of IL-23/ T17 pathogenic axis in psoriasis	5.182E-07	2.528E-06	1.160E-02	3.979E-02	5.182E-07	2.528E-06
167	Immune response_Neurotensin-induced activation of IL-8 in colonocytes	5.368E-07	2.557E-06	8.987E-02	1.809E-01	5.368E-07	2.557E-06
168	Immune response_Murine NKG2D signaling	5.368E-07	2.557E-06	2.126E-02	6.321E-02	5.368E-07	2.557E-06
169	Immune response_PIP3 signaling in B lymphocytes	5.368E-07	2.557E-06	8.595E-06	2.608E-04	5.368E-07	2.557E-06

170	Development_Growth hormone signaling via PI3K/AKT and MAPK cascades	5.368E-07	2.557E-06	2.884E-01	4.101E-01	5.368E-07	2.557E-06
171	Chemotaxis_CCR1 signaling	5.466E-07	2.557E-06	3.447E-01	4.631E-01	5.466E-07	2.557E-06
172	Signal transduction_PTMs in IL-12 signaling pathway	5.466E-07	2.557E-06	1.261E-03	8.217E-03	5.466E-07	2.557E-06
173	Cytoskeleton remodeling_Fibronectin-binding integrins in cell motility	5.490E-07	2.557E-06	1.085E-04	1.601E-03	5.490E-07	2.557E-06
174	Cytoskeleton remodeling_Fibronectin-binding integrins in cell motility	5.490E-07	2.557E-06	1.085E-04	1.601E-03	5.490E-07	2.557E-06
175	Development_Cross-talk between VEGF and Angiopoietin 1 signaling pathways	5.667E-07	2.627E-06	4.875E-01	5.865E-01	5.667E-07	2.627E-06
176	Immune response_Sublytic effects of membrane attack complex	6.567E-07	3.014E-06	2.479E-01	3.676E-01	6.567E-07	3.014E-06
177	Development_Growth factors in regulation of oligodendrocyte precursor cell survival	6.860E-07	3.117E-06	1.384E-02	4.527E-02	6.860E-07	3.117E-06
178	Immune response_Oncostatin M signaling via MAPK in human cells	6.860E-07	3.117E-06	6.659E-02	1.450E-01	6.860E-07	3.117E-06
179	Development_G-CSF signaling	7.372E-07	3.285E-06	3.200E-06	1.317E-04	7.372E-07	3.285E-06
180	Signal transduction_Soluble CXCL16 signaling	7.372E-07	3.285E-06	3.511E-02	9.033E-02	7.372E-07	3.285E-06
181	Apoptosis and survival_Anti-apoptotic action of Gastrin	7.449E-07	3.304E-06	2.299E-02	6.658E-02	7.449E-07	3.304E-06
182	Immune response_Human NKG2D signaling	9.761E-07	4.229E-06	2.555E-03	1.305E-02	9.761E-07	4.229E-06
183	Neurophysiological process_Corticoliberin signaling via CRHR1	9.854E-07	4.229E-06	7.239E-01	7.589E-01	9.854E-07	4.229E-06
184	Transport_The role of AVP in regulation of Aquaporin 2 and renal water reabsorption	9.854E-07	4.229E-06	1.565E-03	9.638E-03	9.854E-07	4.229E-06
185	Immune response_Histamine signaling in dendritic cells	9.854E-07	4.229E-06	3.631E-01	4.773E-01	9.854E-07	4.229E-06
186	Immune response_IL-13 signaling via PI3K-ERK pathway	9.854E-07	4.229E-06	3.631E-01	4.773E-01	9.854E-07	4.229E-06
187	Transport_Macropinocytosis regulation by growth factors	9.985E-07	4.266E-06	5.092E-03	2.200E-02	9.985E-07	4.266E-06
188	Development_Regulation of epithelial-to-mesenchymal transition (EMT)	1.272E-06	5.409E-06	5.502E-03	2.326E-02	1.272E-06	5.409E-06
189	IL-6 signaling in multiple myeloma	1.306E-06	5.529E-06	9.161E-03	3.395E-02	1.306E-06	5.529E-06
190	Development_Insulin, IGF-1 and TNF-alpha in brown adipocyte differentiation	1.718E-06	7.239E-06	1.447E-01	2.492E-01	1.718E-06	7.239E-06
191	G-protein signaling_G-Protein alpha-q signaling cascades	1.752E-06	7.348E-06	5.830E-01	6.494E-01	1.752E-06	7.348E-06
192	Development_TGF-beta-dependent induction of EMT via RhoA, PI3K and ILK.	1.834E-06	7.969E-05	1.834E-06	7.969E-05	2.667E-04	6.150E-04
193	Development_GDNF family signaling	1.869E-06	7.701E-06	1.106E-01	2.074E-01	1.869E-06	7.701E-06
194	Immune response_Inhibitory action of Lipoxins on pro-inflammatory TNF-alpha signaling	1.869E-06	7.701E-06	1.106E-01	2.074E-01	1.869E-06	7.701E-06
195	Development_Endothelin-1/EDNRA transactivation of EGFR	1.869E-06	7.701E-06	1.106E-01	2.074E-01	1.869E-06	7.701E-06
196	Transcription_Androgen Receptor nuclear signaling	1.869E-06	7.701E-06	1.004E-03	6.827E-03	1.869E-06	7.701E-06
197	Cytoskeleton remodeling_Role of PKA in cytoskeleton reorganisation	1.901E-06	7.764E-06	1.805E-02	5.602E-02	1.901E-06	7.764E-06
198	Development_GDNF signaling	2.226E-06	8.970E-06	2.201E-02	6.399E-02	2.226E-06	8.970E-06
199	Immune response_Th17 cell differentiation	2.496E-06	9.975E-06	1.757E-03	1.049E-02	2.496E-06	9.975E-06
200	Development_Angiopoietin - Tie2 signaling	2.496E-06	9.975E-06	5.817E-02	1.315E-01	2.496E-06	9.975E-06
201	Chemotaxis_Leukocyte chemotaxis	2.508E-06	9.980E-06	9.903E-05	1.580E-03	2.508E-06	9.980E-06
202	Immune response_Mast cell proliferation, differentiation and survival	3.086E-06	1.223E-05	8.936E-05	1.519E-03	3.086E-06	1.223E-05
203	Bacterial infections in CF airways	3.293E-06	1.293E-05	3.447E-01	4.631E-01	3.293E-06	1.293E-05
204	Cell adhesion_Integrin-mediated cell adhesion and migration	3.293E-06	1.293E-05	3.288E-02	8.655E-02	3.293E-06	1.293E-05
205	Immune response_TLR3 and TLR4 induced TICAM1-specific signaling pathway	3.350E-06	1.310E-05	9.024E-02	1.809E-01	3.350E-06	1.310E-05
206	Immune response_IL-23 signaling pathway	3.477E-06	1.354E-05	1.314E-01	2.304E-01	3.477E-06	1.354E-05
207	Immune response_IL-9 signaling pathway	3.507E-06	1.354E-05	1.999E-03	1.141E-02	3.507E-06	1.354E-05
208	G-protein signaling_Rac2 regulation pathway	3.507E-06	1.354E-05	2.580E-04	2.883E-03	3.507E-06	1.354E-05
209	Apoptosis and survival_Apoptotic TNF-family pathways	3.534E-06	1.354E-05	2.884E-01	4.101E-01	3.534E-06	1.354E-05
210	Apoptosis and survival_Lymphotoxin-beta receptor signaling	3.534E-06	1.354E-05	6.119E-04	5.037E-03	3.534E-06	1.354E-05
211	Apoptosis and survival_NO synthesis and signaling	3.738E-06	1.426E-05	5.033E-02	1.189E-01	3.738E-06	1.426E-05
212	Blood coagulation_GPIb-IX-V-dependent platelet activation	3.784E-06	1.438E-05	1.249E-02	4.206E-02	3.784E-06	1.438E-05

213	Development_Melanocyte development and pigmentation	4.315E-06	1.626E-05	7.746E-03	2.969E-02	4.315E-06	1.626E-05
214	Th17 cells in CF (mouse model)	4.315E-06	1.626E-05	2.151E-04	2.588E-03	4.315E-06	1.626E-05
215	Development_EPO-induced PI3K/AKT pathway and Ca(2+) influx	4.743E-06	1.766E-05	4.421E-03	1.998E-02	4.743E-06	1.766E-05
216	Chemotaxis_CCL16-, CCL20-, CXCL16- and CCL25-mediated cell migration	4.743E-06	1.766E-05	6.693E-01	7.111E-01	4.743E-06	1.766E-05
217	Development_Adenosine A2A receptor signaling	4.743E-06	1.766E-05	9.489E-02	1.879E-01	4.743E-06	1.766E-05
218	Chemotaxis_CCL2-induced chemotaxis	4.779E-06	1.772E-05	1.345E-02	4.439E-02	4.779E-06	1.772E-05
219	Neuroprotective action of lithium	4.944E-06	1.826E-05	2.143E-01	3.285E-01	4.944E-06	1.826E-05
220	Apoptosis and survival_HTR1A signaling	5.610E-06	2.064E-05	3.631E-01	4.773E-01	5.610E-06	2.064E-05
221	Development_Ligand-independent activation of ESR1 and ESR2	6.303E-06	2.309E-05	4.887E-03	2.123E-02	6.303E-06	2.309E-05
222	Development_Transcription regulation of granulocyte development	6.399E-06	2.326E-05	1.943E-01	3.105E-01	6.399E-06	2.326E-05
223	Development_Angiotensin signaling via STATs	6.399E-06	2.326E-05	1.943E-01	3.105E-01	6.399E-06	2.326E-05
224	Development_SDF-1 signaling in hematopoietic stem cell homing	6.654E-06	2.391E-05	4.009E-05	8.038E-04	6.654E-06	2.391E-05
225	Apoptosis and survival_Anti-apoptotic action of membrane-bound ESR1	6.654E-06	2.391E-05	2.506E-01	3.676E-01	6.654E-06	2.391E-05
226	Development_Gastrin in differentiation of the gastric mucosa	6.654E-06	2.391E-05	6.238E-01	6.804E-01	6.654E-06	2.391E-05
227	Inflammatory factors-induced expression of mucins in normal and asthmatic epithelium	7.238E-06	2.581E-05	1.388E-01	2.412E-01	7.238E-06	2.581E-05
228	Some pathways of EMT in cancer cells	7.238E-06	2.581E-05	2.777E-04	2.934E-03	7.238E-06	2.581E-05
229	Regulation of lipid metabolism_Stimulation of Arachidonic acid production by ACM receptors	7.334E-06	2.605E-05	5.487E-01	6.309E-01	7.334E-06	2.605E-05
230	Development_Regulation of cytoskeleton proteins in oligodendrocyte differentiation and myelination	7.662E-06	2.700E-05	3.369E-03	1.597E-02	7.662E-06	2.700E-05
231	Immune response_PGE2 signaling in immune response	8.299E-06	2.892E-05	2.669E-02	7.454E-02	8.299E-06	2.892E-05
232	Development_Adiponectin signaling	8.299E-06	2.892E-05	3.167E-01	4.352E-01	8.299E-06	2.892E-05
233	Cytoskeleton remodeling_ESR1 action on cytoskeleton remodeling and cell migration	8.725E-06	3.029E-05	2.061E-03	1.145E-02	8.725E-06	3.029E-05
234	Transcription_Receptor-mediated HIF regulation	9.002E-06	3.091E-05	2.600E-01	3.787E-01	9.002E-06	3.091E-05
235	Development_PACAP signaling in neural cells	9.002E-06	3.091E-05	6.334E-01	6.860E-01	9.002E-06	3.091E-05
236	G-protein signaling_Regulation of p38 and JNK signaling mediated by G-proteins	9.002E-06	3.091E-05	7.553E-02	1.583E-01	9.002E-06	3.091E-05
237	Immune response_Antiviral actions of Interferons	9.272E-06	3.172E-05	3.140E-04	3.231E-03	9.272E-06	3.172E-05
238	Immune response_Fc gamma R-mediated phagocytosis in macrophages	1.083E-05	3.623E-05	5.920E-03	2.418E-02	1.083E-05	3.623E-05
239	FGF signaling in pancreatic cancer	1.083E-05	3.623E-05	2.867E-02	7.866E-02	1.083E-05	3.623E-05
240	Cell adhesion_Integrin inside-out signaling in T cells	1.086E-05	3.623E-05	9.903E-05	1.580E-03	1.086E-05	3.623E-05
241	Signal transduction_PTMs in IL-17-induced CIKS-dependent NF-kB signaling and mRNA stabilization	1.149E-05	3.819E-05	1.578E-01	2.665E-01	1.149E-05	3.819E-05
242	Immune response_T regulatory cell-mediated modulation of effector T cell and NK cell functions	1.180E-05	3.891E-05	5.071E-05	9.222E-04	1.180E-05	3.891E-05
243	Cell cycle_Influence of Ras and Rho proteins on G1/S Transition	1.180E-05	3.891E-05	4.491E-02	1.085E-01	1.180E-05	3.891E-05
244	Immune response_IL-17 signaling pathways	1.200E-05	3.944E-05	1.946E-01	3.105E-01	1.200E-05	3.944E-05
245	Apoptosis and survival_NGF activation of NF-kB	1.205E-05	3.946E-05	1.805E-02	5.602E-02	1.205E-05	3.946E-05
246	Signal transduction_Erk Interactions: Inhibition of Erk	1.246E-05	4.052E-05	2.129E-01	3.277E-01	1.246E-05	4.052E-05
247	Immune response_CXCR4 signaling via second messenger	1.246E-05	4.052E-05	2.129E-01	3.277E-01	1.246E-05	4.052E-05
248	Immune response_iC3b-induced phagocytosis via alpha-M/beta-2 integrin	1.489E-05	4.827E-05	4.340E-03	1.973E-02	1.489E-05	4.827E-05
249	Immune response_Immunological synapse formation	1.568E-05	1.257E-04	1.568E-05	4.229E-04	4.393E-05	1.257E-04
250	Immune response_CD137 signaling in immune cell	1.645E-05	5.312E-05	5.825E-03	2.418E-02	1.645E-05	5.312E-05
251	Development_Growth hormone signaling via STATs and PLC/IP3	1.702E-05	5.479E-05	5.936E-01	6.565E-01	1.702E-05	5.479E-05
252	Immune response_C3a signaling	1.801E-05	5.776E-05	1.216E-01	2.208E-01	1.801E-05	5.776E-05
253	Immune response_IL-16 signaling pathway	1.872E-05	5.984E-05	1.629E-01	2.733E-01	1.872E-05	5.984E-05
254	Cell adhesion_Integrin inside-out signaling in neutrophils	1.902E-05	6.059E-05	1.326E-02	4.414E-02	1.902E-05	6.059E-05
255	Cytoskeleton remodeling_Role of PDGFs in cell migration	2.018E-05	6.405E-05	2.201E-02	6.399E-02	2.018E-05	6.405E-05

256	Immune response_IL-4-responsive genes in type 2 immunity	2.102E-05	6.628E-05	8.353E-01	8.505E-01	2.102E-05	6.628E-05
257	Target_metanalisi_Stat-sign	2.182E-05	6.857E-05	1.160E-03	7.750E-03	2.182E-05	6.857E-05
258	Androgen receptor activation and downstream signaling in Prostate cancer	2.250E-05	7.045E-05	1.886E-03	1.109E-02	2.250E-05	7.045E-05
259	Immune response_Differentiation of natural regulatory T cells	2.280E-05	2.666E-04	2.280E-05	5.571E-04	1.024E-04	2.666E-04
260	Development_Adenosine A3 receptor signaling	2.295E-05	7.144E-05	1.272E-01	2.272E-01	2.295E-05	7.144E-05
261	Immune response_IL-22 signaling pathway	2.297E-05	7.144E-05	1.999E-03	1.141E-02	2.297E-05	7.144E-05
262	Development_G-CSF-induced myeloid differentiation	2.313E-05	7.170E-05	8.554E-04	6.251E-03	2.313E-05	7.170E-05
263	Development_ACM2 and ACM4 activation of ERK	2.718E-05	8.343E-05	6.693E-01	7.111E-01	2.718E-05	8.343E-05
264	Immune response_Substance P-stimulated expression of proinflammatory cytokines via MAPKs	2.718E-05	8.343E-05	2.979E-01	4.182E-01	2.718E-05	8.343E-05
265	Immune response_HTR2A-induced activation of cPLA2	2.718E-05	8.343E-05	6.693E-01	7.111E-01	2.718E-05	8.343E-05
266	Neurophysiological process_Constitutive and regulated NMDA receptor trafficking	2.761E-05	8.448E-05	8.077E-01	8.289E-01	2.761E-05	8.448E-05
267	Cytoskeleton remodeling_Integrin outside-in signaling	2.816E-05	2.830E-04	2.816E-05	6.293E-04	1.105E-04	2.830E-04
268	Modulation of tumor response to cytotoxic T cells by hypoxia in tumors	2.904E-05	8.827E-05	3.743E-02	9.413E-02	2.904E-05	8.827E-05
269	Development_TGF-beta receptor signaling	2.904E-05	8.827E-05	2.448E-04	2.815E-03	2.904E-05	8.827E-05
270	Resolution of inflammation in healing myocardial infarction	2.957E-05	8.899E-05	4.742E-01	5.713E-01	2.957E-05	8.899E-05
271	Development_Angiotensin signaling via beta-Arrestin	2.957E-05	8.899E-05	4.742E-01	5.713E-01	2.957E-05	8.899E-05
272	Transcription_Transcription regulation of aminoacid metabolism	2.957E-05	8.899E-05	4.742E-01	5.713E-01	2.957E-05	8.899E-05
273	Cell adhesion_Role of tetraspanins in the integrin-mediated cell adhesion	3.063E-05	9.129E-05	2.265E-03	1.213E-02	3.063E-05	9.129E-05
274	Immune response_Generation of memory CD4+ T cells	3.063E-05	9.129E-05	3.342E-05	7.260E-04	3.063E-05	9.129E-05
275	Role of red blood cell adhesion to endothelium in vaso-occlusion in Sickle cell disease	3.063E-05	9.129E-05	6.659E-02	1.450E-01	3.063E-05	9.129E-05
276	IL-1 beta-dependent CFTR expression	3.200E-05	9.509E-05	5.494E-01	6.309E-01	3.200E-05	9.509E-05
277	Immune response_CD40 signaling	3.360E-05	9.951E-05	5.936E-03	2.418E-02	3.360E-05	9.951E-05
278	Development_FGF2-dependent induction of EMT	3.414E-05	1.008E-04	9.024E-02	1.809E-01	3.414E-05	1.008E-04
279	K-RAS signaling in pancreatic cancer	3.501E-05	1.027E-04	3.073E-01	4.245E-01	3.501E-05	1.027E-04
280	Development_Activation of Erk by ACM1, ACM3 and ACM5	3.501E-05	1.027E-04	3.073E-01	4.245E-01	3.501E-05	1.027E-04
281	Hyaluronic acid/ CD44 signaling in cancer	3.579E-05	1.043E-04	1.817E-01	2.960E-01	3.579E-05	1.043E-04
282	Cell adhesion_Integrin inside-out signaling	3.579E-05	1.043E-04	3.369E-03	1.597E-02	3.579E-05	1.043E-04
283	Transcription_Role of AP-1 in regulation of cellular metabolism	4.009E-05	8.038E-04	4.009E-05	8.038E-04	4.279E-03	7.428E-03
284	Immune response_IL-7 signaling in T lymphocytes	4.039E-05	1.170E-04	2.555E-03	1.305E-02	4.039E-05	1.170E-04
285	Substance P-mediated inflammation and pain in Sickle cell disease	4.039E-05	1.170E-04	6.238E-01	6.804E-01	4.039E-05	1.170E-04
286	Signal transduction_ERK1/2 signaling pathway	4.364E-05	1.257E-04	1.943E-01	3.105E-01	4.364E-05	1.257E-04
287	Signal transduction_mTORC1 upstream signaling	4.373E-05	1.257E-04	3.945E-02	9.814E-02	4.373E-05	1.257E-04
288	Cell adhesion_Histamine H1 receptor signaling in the interruption of cell barrier integrity	4.472E-05	1.272E-04	2.669E-02	7.454E-02	4.472E-05	1.272E-04
289	Development_Membrane-bound ESR1: interaction with growth factors signaling	4.472E-05	1.272E-04	2.669E-02	7.454E-02	4.472E-05	1.272E-04
290	Development_Growth factors in regulation of oligodendrocyte precursor cell proliferation	4.909E-05	1.392E-04	6.878E-03	2.650E-02	4.909E-05	1.392E-04
291	Development_ERBB-family signaling	5.273E-05	1.486E-04	7.553E-02	1.583E-01	5.273E-05	1.486E-04
292	Immune response_TSLP signalling	5.273E-05	1.486E-04	1.657E-02	5.253E-02	5.273E-05	1.486E-04
293	Immune response_LTBR1 signaling	5.365E-05	1.507E-04	1.946E-01	3.105E-01	5.365E-05	1.507E-04
294	Translation_Regulation of EIF4F activity	5.656E-05	1.573E-04	2.123E-03	1.145E-02	5.656E-05	1.573E-04
295	Development_Role of HDAC and calcium/calmodulin-dependent kinase (CaMK) in control of skeletal myogenesis	5.656E-05	1.573E-04	1.507E-01	2.567E-01	5.656E-05	1.573E-04
296	Development_G-protein-mediated regulation of MAPK-ERK signaling	5.668E-05	1.573E-04	1.106E-01	2.074E-01	5.668E-05	1.573E-04
297	Immune response_Th1 and Th2 cell differentiation	5.671E-05	1.864E-04	5.671E-05	1.008E-03	6.816E-05	1.864E-04
298	G-protein signaling_N-RAS regulation pathway	5.871E-05	1.615E-04	5.721E-01	6.446E-01	5.871E-05	1.615E-04

299	Immune response_ETV3 affect on CSF1-promoted macrophage differentiation	5.871E-05	1.615E-04	2.036E-01	3.196E-01	5.871E-05	1.615E-04
300	Chemotaxis_CCL19- and CCl21-mediated chemotaxis	5.871E-05	1.615E-04	2.036E-01	3.196E-01	5.871E-05	1.615E-04
301	Transcription_Role of VDR in regulation of genes involved in osteoporosis	6.520E-05	1.788E-04	4.340E-03	1.973E-02	6.520E-05	1.788E-04
302	Immune response_NF-AT signaling and leukocyte interactions	7.131E-05	1.939E-04	6.491E-03	2.551E-02	7.131E-05	1.939E-04
303	Development_PIP3 signaling in cardiac myocytes	7.131E-05	1.939E-04	6.491E-03	2.551E-02	7.131E-05	1.939E-04
304	Development_Thrombopoetin signaling via JAK-STAT pathway	7.635E-05	2.070E-04	2.061E-03	1.145E-02	7.635E-05	2.070E-04
305	Signal transduction_PTMs in BAFF-induced non-canonical NF-kB signaling	7.798E-05	2.101E-04	1.031E-02	3.647E-02	7.798E-05	2.101E-04
306	G-protein signaling_G-Protein beta/gamma signaling cascades	7.798E-05	2.101E-04	5.416E-02	1.249E-01	7.798E-05	2.101E-04
307	Development_Delta-type opioid receptor signaling via G-protein alpha-14	8.252E-05	2.211E-04	1.578E-01	2.665E-01	8.252E-05	2.211E-04
308	Development_Thrombospondin-1 signaling	8.252E-05	2.211E-04	5.133E-01	6.081E-01	8.252E-05	2.211E-04
309	Immune response_T cell co-signaling receptors, schema	8.557E-05	2.279E-04	1.250E-02	4.206E-02	8.557E-05	2.279E-04
310	Aberrant B-Raf signaling in melanoma progression	8.557E-05	2.279E-04	4.084E-01	5.201E-01	8.557E-05	2.279E-04
311	DeltaF508-CFTR traffic / ER-to-Golgi in CF	8.662E-05	2.293E-04	3.540E-01	4.692E-01	8.662E-05	2.293E-04
312	wtCFTR traffic / ER-to-Golgi (normal)	8.662E-05	2.293E-04	3.540E-01	4.692E-01	8.662E-05	2.293E-04
313	Breast cancer (general schema)	8.730E-05	2.293E-04	1.961E-02	5.947E-02	8.730E-05	2.293E-04
314	Neurophysiological process_Netrin-1 in regulation of axon guidance	8.730E-05	2.293E-04	8.497E-02	1.744E-01	8.730E-05	2.293E-04
315	Development_Neurotrophin family signaling	8.730E-05	2.293E-04	5.357E-04	4.604E-03	8.730E-05	2.293E-04
316	Oxidative stress_Angiotensin II-induced production of ROS	1.024E-04	2.666E-04	2.223E-01	3.382E-01	1.024E-04	2.666E-04
317	NALP3 inflammasome activation in age-related macular degeneration (AMD)	1.024E-04	2.666E-04	2.223E-01	3.382E-01	1.024E-04	2.666E-04
318	Development_Keratinocyte differentiation	1.044E-04	2.710E-04	5.316E-02	1.248E-01	1.044E-04	2.710E-04
319	Cytoskeleton remodeling_Reverse signaling by ephrin B	1.085E-04	1.601E-03	1.085E-04	1.601E-03	4.926E-03	8.487E-03
320	Alternative complement cascade disruption in age-related macular degeneration	1.085E-04	1.601E-03	1.085E-04	1.601E-03	1.069E-03	2.154E-03
321	Proteolysis_Putative ubiquitin pathway	1.099E-04	2.830E-04	4.464E-01	5.550E-01	1.099E-04	2.830E-04
322	Development_EGFR signaling via PIP3	1.099E-04	2.830E-04	2.446E-03	1.293E-02	1.099E-04	2.830E-04
323	Transcription_N-CoR/ SMRT complex-mediated epigenetic gene silencing	1.105E-04	2.830E-04	3.511E-02	9.033E-02	1.105E-04	2.830E-04
324	Role and regulation of Prostaglandin E2 in gastric cancer	1.105E-04	2.830E-04	1.272E-01	2.272E-01	1.105E-04	2.830E-04
325	Apoptosis and survival_Anti-apoptotic TNFs/NF-kB/Bcl-2 pathway	1.109E-04	2.831E-04	6.119E-04	5.037E-03	1.109E-04	2.831E-04
326	Neurophysiological process_NMDA-dependent postsynaptic long-term potentiation in CA1 hippocampal neurons	1.178E-04	3.000E-04	6.062E-01	6.676E-01	1.178E-04	3.000E-04
327	Immune response_IL-5 signaling via JAK/STAT	1.266E-04	3.216E-04	5.609E-02	1.286E-01	1.266E-04	3.216E-04
328	Immune response_IL-12-induced IFN-gamma production	1.329E-04	3.364E-04	1.258E-02	4.206E-02	1.329E-04	3.364E-04
329	Multiple myeloma (general schema)	1.332E-04	3.364E-04	7.508E-02	1.583E-01	1.332E-04	3.364E-04
330	Signal transduction_AKT signaling	1.396E-04	3.498E-04	6.963E-04	5.556E-03	1.396E-04	3.498E-04
331	Regulation of Tissue factor signaling in cancer	1.396E-04	3.498E-04	2.979E-01	4.182E-01	1.396E-04	3.498E-04
332	Apoptosis and survival_TNF-alpha-induced Caspase-8 signaling	1.396E-04	3.498E-04	9.489E-02	1.879E-01	1.396E-04	3.498E-04
333	Development_Slit-Robo signaling	1.504E-04	3.739E-04	8.554E-04	6.251E-03	1.504E-04	3.739E-04
334	Inter-cellular relations in COPD (general schema)	1.504E-04	3.739E-04	6.590E-03	2.551E-02	1.504E-04	3.739E-04
335	Immune response_IL-27 signaling pathway	1.548E-04	3.826E-04	2.201E-02	6.399E-02	1.548E-04	3.826E-04
336	G-protein signaling_M-RAS regulation pathway	1.548E-04	3.826E-04	4.605E-01	5.635E-01	1.548E-04	3.826E-04
337	Immune response_Antigen presentation by MHC class II	1.704E-04	2.149E-03	1.704E-04	2.149E-03	4.735E-02	6.795E-02
338	Development_Beta adrenergic receptors in brown adipocyte differentiation	1.708E-04	4.188E-04	6.659E-02	1.450E-01	1.708E-04	4.188E-04
339	G-protein signaling_H-RAS regulation pathway	1.708E-04	4.188E-04	2.411E-01	3.585E-01	1.708E-04	4.188E-04
340	Immune response_IL-13 signaling via JAK-STAT	1.745E-04	4.245E-04	1.000E-01	1.936E-01	1.745E-04	4.245E-04
341	Immune response_CRTH2 signaling in Th2 cells	1.745E-04	4.245E-04	3.073E-01	4.245E-01	1.745E-04	4.245E-04

342	Development_S1P1 signaling pathway	1.745E-04	4.245E-04	1.000E-01	1.936E-01	1.745E-04	4.245E-04
343	LRRK2 and immune function in Parkinson's disease	1.866E-04	2.280E-03	1.866E-04	2.280E-03	3.349E-03	5.971E-03
344	Ligand-independent activation of Androgen receptor in Prostate Cancer	1.910E-04	4.636E-04	2.748E-04	2.934E-03	1.910E-04	4.636E-04
345	Development_Activation of ERK by Kappa-type opioid receptor	1.989E-04	4.801E-04	5.494E-01	6.309E-01	1.989E-04	4.801E-04
346	Development_Transactivation of PDGFR in non-neuronal cells by Dopamine D2 receptor	1.989E-04	4.801E-04	1.850E-01	2.996E-01	1.989E-04	4.801E-04
347	Development_Glucocorticoid receptor signaling	2.137E-04	5.118E-04	3.537E-04	3.505E-03	2.137E-04	5.118E-04
348	G-protein signaling_TC21 regulation pathway	2.137E-04	5.118E-04	4.742E-01	5.713E-01	2.137E-04	5.118E-04
349	G-protein signaling_R-RAS regulation pathway	2.137E-04	5.118E-04	1.314E-01	2.304E-01	2.137E-04	5.118E-04
350	Development_Activation of ERK by Alpha-1 adrenergic receptors	2.165E-04	5.140E-04	6.859E-01	7.258E-01	2.165E-04	5.140E-04
351	Cell adhesion_Ephrin signaling	2.165E-04	5.140E-04	1.053E-01	2.022E-01	2.165E-04	5.140E-04
352	Role of alpha-6/beta-4 integrins in carcinoma progression	2.165E-04	5.140E-04	2.669E-02	7.454E-02	2.165E-04	5.140E-04
353	G-protein signaling_G-Protein alpha-12 signaling pathway	2.173E-04	5.140E-04	1.516E-02	4.900E-02	2.173E-04	5.140E-04
354	Signal transduction_Cyclic AMP signaling	2.173E-04	5.140E-04	6.238E-01	6.804E-01	2.173E-04	5.140E-04
355	Development_TGF-beta-dependent induction of EMT via SMADs	2.196E-04	2.592E-03	2.196E-04	2.592E-03	2.483E-03	4.594E-03
356	Nociception_Nociceptin receptor signaling	2.227E-04	5.253E-04	4.347E-02	1.056E-01	2.227E-04	5.253E-04
357	Translation_Translation regulation by Alpha-1 adrenergic receptors	2.463E-04	5.795E-04	3.904E-01	5.030E-01	2.463E-04	5.795E-04
358	Putative pathways for stimulation of fat cell differentiation by Bisphenol A	2.596E-04	6.093E-04	4.656E-02	1.117E-01	2.596E-04	6.093E-04
359	High shear stress-induced platelet activation	2.667E-04	6.150E-04	1.106E-01	2.074E-01	2.667E-04	6.150E-04
360	Chemotaxis_Lipoxin inhibitory action on Formyl-Met-Leu-Phe-induced neutrophil chemotaxis	2.667E-04	6.150E-04	5.920E-03	2.418E-02	2.667E-04	6.150E-04
361	Development_Angiotensin activation of Akt	2.667E-04	6.150E-04	3.260E-01	4.442E-01	2.667E-04	6.150E-04
362	Neurophysiological process_ACM regulation of nerve impulse	2.667E-04	6.150E-04	6.939E-01	7.333E-01	2.667E-04	6.150E-04
363	Regulation of GSK3 beta in bipolar disorder	2.667E-04	6.150E-04	3.260E-01	4.442E-01	2.667E-04	6.150E-04
364	Signal transduction_Calcium signaling	2.667E-04	6.150E-04	1.106E-01	2.074E-01	2.667E-04	6.150E-04
365	Cell adhesion_PLAU signaling	2.741E-04	6.290E-04	7.553E-02	1.583E-01	2.741E-04	6.290E-04
366	Immune response_Differentiation and clonal expansion of CD8+ T cells	2.741E-04	6.290E-04	4.056E-04	3.731E-03	2.741E-04	6.290E-04
367	Development_TGF-beta-induction of EMT via ROS	2.864E-04	6.556E-04	9.024E-02	1.809E-01	2.864E-04	6.556E-04
368	G-protein signaling_Ras family GTPases in kinase cascades (schema)	2.898E-04	6.601E-04	4.289E-04	3.900E-03	2.898E-04	6.601E-04
369	Development_Hedgehog signaling	3.264E-04	7.379E-04	1.160E-01	2.125E-01	3.264E-04	7.379E-04
370	Development_Leptin signaling via PI3K-dependent pathway	3.264E-04	7.379E-04	1.160E-01	2.125E-01	3.264E-04	7.379E-04
371	Development_Angiotensin activation of ERK	3.350E-04	7.537E-04	5.721E-01	6.446E-01	3.350E-04	7.537E-04
372	Cytoskeleton remodeling_Thyroliberin in cytoskeleton remodeling	3.350E-04	7.537E-04	5.721E-01	6.446E-01	3.350E-04	7.537E-04
373	Development_Role of Activin A in cell differentiation and proliferation	3.428E-04	7.675E-04	8.019E-02	1.677E-01	3.428E-04	7.675E-04
374	Apoptosis and survival_Ceramides signaling pathway	3.428E-04	7.675E-04	2.695E-01	3.888E-01	3.428E-04	7.675E-04
375	PGE2 pathways in cancer	3.557E-04	7.945E-04	1.629E-01	2.733E-01	3.557E-04	7.945E-04
376	Transport_Clathrin-coated vesicle cycle	3.615E-04	8.055E-04	2.075E-03	1.145E-02	3.615E-04	8.055E-04
377	Neurophysiological process_Activity-dependent synaptic AMPA receptor removal	3.658E-04	8.133E-04	2.148E-02	6.339E-02	3.658E-04	8.133E-04
378	Apoptosis and survival_Anti-apoptotic TNFs/NF-kB/IAP pathway	3.867E-04	8.555E-04	1.489E-01	2.553E-01	3.867E-04	8.555E-04
379	HCV-dependent regulation of membrane receptors signaling in HCC	3.867E-04	8.555E-04	3.010E-02	8.200E-02	3.867E-04	8.555E-04
380	Multiple sclerosis (general schema)	4.038E-04	8.911E-04	9.813E-02	1.918E-01	4.038E-04	8.911E-04
381	Airway smooth muscle contraction in asthma	4.242E-04	9.216E-04	7.635E-01	7.929E-01	4.242E-04	9.216E-04
382	Muscle contraction_ACM regulation of smooth muscle contraction	4.242E-04	9.216E-04	7.635E-01	7.929E-01	4.242E-04	9.216E-04
383	Development_Growth hormone-releasing hormone (GH-RH) signaling	4.252E-04	9.216E-04	2.790E-01	4.003E-01	4.252E-04	9.216E-04
384	TLR2-induced platelet activation	4.252E-04	9.216E-04	2.790E-01	4.003E-01	4.252E-04	9.216E-04

385	Possible pathway of TGF-beta 1-dependent inhibition of CFTR expression	4.252E-04	9.216E-04	8.497E-02	1.744E-01	4.252E-04	9.216E-04
386	Development_CNTF receptor signaling	4.275E-04	9.216E-04	2.129E-01	3.277E-01	4.275E-04	9.216E-04
387	Apoptosis and survival_Role of CDK5 in neuronal death and survival	4.275E-04	9.216E-04	1.538E-03	9.620E-03	4.275E-04	9.216E-04
388	Oxidative stress_ROS-mediated activation of MAPK via inhibition of phosphatases	4.275E-04	9.216E-04	5.830E-01	6.494E-01	4.275E-04	9.216E-04
389	Development_Role of CDK5 in neuronal development	4.275E-04	9.216E-04	1.031E-02	3.647E-02	4.275E-04	9.216E-04
390	Development_Role of CNTF and LIF in regulation of oligodendrocyte development	5.084E-04	1.093E-03	5.133E-01	6.081E-01	5.084E-04	1.093E-03
391	Translation_Non-genomic (rapid) action of Androgen Receptor	5.234E-04	1.123E-03	2.126E-02	6.321E-02	5.234E-04	1.123E-03
392	Altered Ca2+ handling in heart failure	5.401E-04	1.154E-03	5.936E-01	6.565E-01	5.401E-04	1.154E-03
393	Development_Transcriptional regulation of megakaryopoiesis	5.401E-04	1.154E-03	1.757E-03	1.049E-02	5.401E-04	1.154E-03
394	Expression targets of Tissue factor signaling in cancer	5.565E-04	1.183E-03	4.320E-01	5.405E-01	5.565E-04	1.183E-03
395	Immune response_IL-15 signaling via JAK-STAT cascade	5.565E-04	1.183E-03	1.062E-01	2.026E-01	5.565E-04	1.183E-03
396	Development_EDNRB signaling	5.767E-04	1.223E-03	3.631E-01	4.773E-01	5.767E-04	1.223E-03
397	Immune response_Reactive oxygen species (ROS) in IL-4 signaling	5.876E-04	1.244E-03	8.613E-02	1.758E-01	5.876E-04	1.244E-03
398	Immune response_IL-7 signaling in B lymphocytes	6.397E-04	1.345E-03	6.963E-04	5.556E-03	6.397E-04	1.345E-03
399	Nicotine signaling in dopaminergic neurons, Pt. 2 - axon terminal	6.397E-04	1.345E-03	6.693E-01	7.111E-01	6.397E-04	1.345E-03
400	Apoptosis and survival_TNFR1 signaling pathway	6.397E-04	1.345E-03	2.979E-01	4.182E-01	6.397E-04	1.345E-03
401	Proteolysis_Putative SUMO-1 pathway	6.593E-04	1.380E-03	3.624E-02	9.231E-02	6.593E-04	1.380E-03
402	Immune response_Delta-type opioid receptor signaling in T-cells	6.593E-04	1.380E-03	5.825E-03	2.418E-02	6.593E-04	1.380E-03
403	Development_Lipoxin inhibitory action on PDGF, EGF and LTD4 signaling	6.758E-04	1.411E-03	2.317E-01	3.478E-01	6.758E-04	1.411E-03
404	Neurophysiological process_Constitutive and activity-dependent synaptic AMPA receptor delivery	7.001E-04	1.458E-03	6.218E-02	1.380E-01	7.001E-04	1.458E-03
405	DNA damage_Role of SUMO in p53 regulation	7.410E-04	5.738E-03	7.410E-04	5.738E-03	2.772E-02	4.183E-02
406	Immune response_IL-12 signaling pathway	7.519E-04	1.559E-03	1.144E-01	2.121E-01	7.519E-04	1.559E-03
407	Cytoskeleton remodeling_Regulation of actin cytoskeleton by Rho GTPases	7.519E-04	1.559E-03	1.962E-02	5.947E-02	7.519E-04	1.559E-03
408	Aberrant lipid trafficking and metabolism in age-related macular degeneration pathogenesis	7.631E-04	5.850E-03	7.631E-04	5.850E-03	5.800E-02	8.158E-02
409	Apoptosis and survival_FAS signaling cascades	7.765E-04	1.607E-03	3.073E-01	4.245E-01	7.765E-04	1.607E-03
410	Development_Beta-adrenergic receptors signaling via Cyclic AMP	8.199E-04	1.693E-03	7.378E-01	7.714E-01	8.199E-04	1.693E-03
411	Cytoskeleton remodeling_RalA regulation pathway	8.554E-04	6.251E-03	8.554E-04	6.251E-03	1.649E-02	2.583E-02
412	Neurophysiological process_Receptor-mediated axon growth repulsion	9.365E-04	1.921E-03	1.053E-01	2.022E-01	9.365E-04	1.921E-03
413	Cell adhesion_ECM remodeling	9.701E-04	1.981E-03	1.074E-02	3.734E-02	9.701E-04	1.981E-03
414	Development_Differentiation of white adipocytes	9.701E-04	1.981E-03	2.123E-03	1.145E-02	9.701E-04	1.981E-03
415	Signal transduction_PTEN pathway	1.004E-03	2.257E-03	1.004E-03	6.827E-03	1.123E-03	2.257E-03
416	Role of Endothelin-1 in inflammation and vasoconstriction in Sickle cell disease	1.031E-03	2.100E-03	2.506E-01	3.676E-01	1.031E-03	2.100E-03
417	Development_Positive regulation of STK3/4 (Hippo) pathway and negative regulation of YAP/TAZ function	1.051E-03	2.137E-03	2.616E-01	3.803E-01	1.051E-03	2.137E-03
418	Development_Role of proteases in hematopoietic stem cell mobilization	1.062E-03	2.154E-03	9.920E-03	3.579E-02	1.062E-03	2.154E-03
419	Apoptosis and survival_Role of IAP-proteins in apoptosis	1.069E-03	2.154E-03	5.494E-01	6.309E-01	1.069E-03	2.154E-03
420	Influence of low doses of Arsenite on glucose uptake in adipocytes	1.069E-03	2.154E-03	4.297E-02	1.047E-01	1.069E-03	2.154E-03
421	Impaired inhibitory action of lipoxins on ROS production in neutrophils in CF	1.142E-03	2.292E-03	3.994E-01	5.129E-01	1.142E-03	2.292E-03
422	Blood coagulation_GPCRs in platelet aggregation	1.206E-03	2.414E-03	3.386E-02	8.827E-02	1.206E-03	2.414E-03
423	Cell adhesion_Role of CDK5 in cell adhesion	1.209E-03	8.011E-03	1.209E-03	8.011E-03	2.117E-02	3.265E-02
424	Transcription_PPAR Pathway	1.288E-03	2.573E-03	2.143E-01	3.285E-01	1.288E-03	2.573E-03
425	Development_Leptin signaling via JAK/STAT and MAPK cascades	1.303E-03	2.586E-03	1.314E-01	2.304E-01	1.303E-03	2.586E-03
426	Development_Signaling of Beta-adrenergic receptors via Beta-arrestins	1.303E-03	2.586E-03	4.742E-01	5.713E-01	1.303E-03	2.586E-03
427	Putative pathways of hormone action in neurofibromatosis type 1	1.303E-03	2.586E-03	2.455E-02	7.008E-02	1.303E-03	2.586E-03

428	Immune response_PGE2 common pathways	1.338E-03	2.613E-03	7.017E-01	7.376E-01	1.338E-03	2.613E-03
429	Regulation of lipid metabolism_Insulin signaling: generic cascades	1.338E-03	2.613E-03	3.073E-02	8.259E-02	1.338E-03	2.613E-03
430	Development_Beta-adrenergic receptor-induced regulation of ERK	1.338E-03	2.613E-03	1.160E-01	2.125E-01	1.338E-03	2.613E-03
431	Signal transduction_JNK pathway	1.338E-03	2.613E-03	6.491E-03	2.551E-02	1.338E-03	2.613E-03
432	Development_WNT5A signaling	1.338E-03	2.613E-03	3.073E-02	8.259E-02	1.338E-03	2.613E-03
433	Development_HGF signaling pathway	1.338E-03	2.613E-03	6.491E-03	2.551E-02	1.338E-03	2.613E-03
434	Apoptosis and survival_Endoplasmic reticulum stress response pathway	1.339E-03	2.613E-03	4.084E-01	5.201E-01	1.339E-03	2.613E-03
435	Tau pathology in Alzheimer disease	1.339E-03	2.613E-03	4.084E-01	5.201E-01	1.339E-03	2.613E-03
436	Signal transduction_mTORC2 upstream signaling	1.353E-03	8.742E-03	1.353E-03	8.742E-03	5.942E-03	1.002E-02
437	Blood coagulation_Platelet microparticle generation	1.379E-03	2.680E-03	5.487E-01	6.309E-01	1.379E-03	2.680E-03
438	wtCFTR and deltaF508-CFTR traffic / Clathrin coated vesicles formation (normal and CF)	1.421E-03	9.033E-03	1.421E-03	9.033E-03	1.087E-02	1.750E-02
439	Development_WNT signaling pathway. Part 1. Degradation of beta-catenin in the absence WNT signaling	1.461E-03	2.821E-03	8.255E-02	1.703E-01	1.461E-03	2.821E-03
440	Translation_IL-2 regulation of translation	1.461E-03	2.821E-03	1.156E-02	3.979E-02	1.461E-03	2.821E-03
441	Development_YAP/TAZ-mediated co-regulation of transcription	1.563E-03	3.011E-03	2.819E-03	1.406E-02	1.563E-03	3.011E-03
442	Development_Activation of astroglial cells proliferation by ACM3	1.660E-03	3.192E-03	2.036E-01	3.196E-01	1.660E-03	3.192E-03
443	Cell adhesion_Cadherin-mediated cell adhesion	1.676E-03	3.217E-03	3.891E-03	1.801E-02	1.676E-03	3.217E-03
444	Ca(2+)-dependent NF-AT signaling in cardiac hypertrophy	1.816E-03	3.478E-03	7.696E-01	7.981E-01	1.816E-03	3.478E-03
445	Development_Regulation of lung epithelial progenitor cell differentiation	1.832E-03	3.502E-03	3.586E-03	1.669E-02	1.832E-03	3.502E-03
446	Immune response_Oncostatin M signaling via JAK-Stat in mouse cells	1.964E-03	3.746E-03	9.024E-02	1.809E-01	1.964E-03	3.746E-03
447	Immune response _Sialic-acid receptors (Siglecs) signaling	1.997E-03	3.802E-03	4.769E-02	1.130E-01	1.997E-03	3.802E-03
448	G-protein signaling_Regulation of RAC1 activity	1.999E-03	5.430E-03	1.999E-03	1.141E-02	2.999E-03	5.430E-03
449	Immune response_Inflammasome in inflammatory response	2.039E-03	3.818E-03	5.830E-01	6.494E-01	2.039E-03	3.818E-03
450	G-protein signaling_RhoA regulation pathway	2.039E-03	3.818E-03	5.416E-02	1.249E-01	2.039E-03	3.818E-03
451	Development_S1P1 receptor signaling via beta-arrestin	2.039E-03	3.818E-03	2.129E-01	3.277E-01	2.039E-03	3.818E-03
452	Immune response_TLR ligands	2.039E-03	3.818E-03	5.416E-02	1.249E-01	2.039E-03	3.818E-03
453	Development_Epigenetic and transcriptional regulation of oligodendrocyte precursor cell differentiation and myelination	2.039E-03	3.818E-03	2.129E-01	3.277E-01	2.039E-03	3.818E-03
454	G-protein signaling_RhoA regulation pathway	2.039E-03	3.818E-03	5.416E-02	1.249E-01	2.039E-03	3.818E-03
455	Apoptosis and survival_Cytoplasmic/mitochondrial transport of proapoptotic proteins Bid, Bmf and Bim	2.039E-03	3.818E-03	5.830E-01	6.494E-01	2.039E-03	3.818E-03
456	Development_Role of cell-cell and ECM-cell interactions in oligodendrocyte differentiation and myelination	2.039E-03	3.818E-03	2.129E-01	3.277E-01	2.039E-03	3.818E-03
457	G-protein signaling_G-Protein alpha-i signaling cascades	2.127E-03	3.976E-03	1.489E-01	2.553E-01	2.127E-03	3.976E-03
458	Immune response_Inhibitory action of lipoxins on superoxide production induced by IL-8 and Leukotriene B4 in neutrophils	2.192E-03	4.080E-03	3.631E-01	4.773E-01	2.192E-03	4.080E-03
459	Transcription_Role of the non-genomic action of Retinoic acid and phosphorylation of Retinoic acid receptors in the initiation of transcription	2.483E-03	4.594E-03	5.817E-02	1.315E-01	2.483E-03	4.594E-03
460	Oxidative stress_NOX and DUOX families of NADPH oxidases	2.483E-03	4.594E-03	2.223E-01	3.382E-01	2.483E-03	4.594E-03
461	Cell cycle_Regulation of G1/S transition (part 1)	2.555E-03	1.305E-02	2.555E-03	1.305E-02	1.542E-02	2.439E-02
462	Chemotaxis_Inhibitory action of lipoxins on IL-8- and Leukotriene B4-induced neutrophil migration	2.558E-03	4.724E-03	9.161E-03	3.395E-02	2.558E-03	4.724E-03
463	Development_Role of HGF in hematopoietic stem cell mobilization	2.588E-03	4.753E-03	9.813E-02	1.918E-01	2.588E-03	4.753E-03
464	Transcription_Role of Akt in hypoxia induced HIF1 activation	2.588E-03	4.753E-03	9.813E-02	1.918E-01	2.588E-03	4.753E-03
465	Development_S1P3 receptor signaling pathway	2.599E-03	4.753E-03	9.489E-02	1.879E-01	2.599E-03	4.753E-03
466	Chemotaxis_C5a-induced chemotaxis	2.599E-03	4.753E-03	9.489E-02	1.879E-01	2.599E-03	4.753E-03
467	Development_Notch Signaling Pathway	2.599E-03	4.753E-03	2.979E-01	4.182E-01	2.599E-03	4.753E-03
468	Development_Mu-type opioid receptor regulation of proliferation	2.667E-03	4.857E-03	1.578E-01	2.665E-01	2.667E-03	4.857E-03
469	Development_Regulation of CDK5 in CNS	2.667E-03	4.857E-03	3.309E-02	8.655E-02	2.667E-03	4.857E-03
470	Transcription_P53 signaling pathway	2.871E-03	8.668E-03	2.871E-03	1.406E-02	5.059E-03	8.668E-03

471	Cell adhesion_Endothelial cell contacts by non-junctional mechanisms	2.878E-03	1.406E-02	2.878E-03	1.406E-02	8.413E-02	1.144E-01
472	Cell adhesion_Endothelial cell contacts by non-junctional mechanisms	2.878E-03	1.406E-02	2.878E-03	1.406E-02	8.413E-02	1.144E-01
473	Upregulation of MITF in melanoma	2.999E-03	5.430E-03	2.317E-01	3.478E-01	2.999E-03	5.430E-03
474	Development_Hedgehog and PTH signaling pathways in bone and cartilage development	2.999E-03	5.430E-03	2.317E-01	3.478E-01	2.999E-03	5.430E-03
475	Impaired Lipoxin A4 signaling in CF	3.068E-03	5.533E-03	3.073E-01	4.245E-01	3.068E-03	5.533E-03
476	Retinal ganglion cell damage in glaucoma	3.068E-03	5.533E-03	4.887E-03	2.123E-02	3.068E-03	5.533E-03
477	Apoptosis and survival_nAChR in apoptosis inhibition and cell cycle progression	3.306E-03	5.927E-03	1.668E-01	2.769E-01	3.306E-03	5.927E-03
478	Apoptosis and survival_p53-dependent apoptosis	3.306E-03	5.927E-03	1.668E-01	2.769E-01	3.306E-03	5.927E-03
479	Immune response_Oncostatin M signaling via JAK-Stat in human cells	3.349E-03	5.971E-03	1.062E-01	2.026E-01	3.349E-03	5.971E-03
480	Regulation of lipid metabolism_Regulation of lipid metabolism by niacin and isoprenaline	3.601E-03	6.395E-03	3.167E-01	4.352E-01	3.601E-03	6.395E-03
481	Neurophysiological process_Glutamic acid regulation of Dopamine D1A receptor signaling	3.601E-03	6.395E-03	6.859E-01	7.258E-01	3.601E-03	6.395E-03
482	Regulation of CFTR activity (normal and CF)	3.631E-03	6.423E-03	2.076E-01	3.241E-01	3.631E-03	6.423E-03
483	Development_Alpha-2 adrenergic receptor activation of ERK	3.631E-03	6.423E-03	4.689E-01	5.712E-01	3.631E-03	6.423E-03
484	Regulation of AKT(PKB)/ GSK3 beta cascade in bipolar disorder	3.960E-03	6.992E-03	1.567E-01	2.664E-01	3.960E-03	6.992E-03
485	Colorectal cancer (general schema)	4.055E-03	7.132E-03	1.759E-01	2.877E-01	4.055E-03	7.132E-03
486	Apoptosis and survival_Granzyme A signaling	4.055E-03	7.132E-03	6.590E-03	2.551E-02	4.055E-03	7.132E-03
487	Immune response_IL-4-induced regulators of cell growth, survival, differentiation and metabolism	4.126E-03	7.217E-03	2.148E-02	6.339E-02	4.126E-03	7.217E-03
488	Action of GSK3 beta in bipolar disorder	4.266E-03	7.428E-03	1.144E-01	2.121E-01	4.266E-03	7.428E-03
489	Apoptosis and survival_NO signaling in apoptosis	4.266E-03	7.428E-03	4.464E-01	5.550E-01	4.266E-03	7.428E-03
490	Development_Mu-type opioid receptor signaling	4.279E-03	7.428E-03	2.506E-01	3.676E-01	4.279E-03	7.428E-03
491	Transport_Alpha-2 adrenergic receptor regulation of ion channels	4.883E-03	8.445E-03	7.017E-01	7.376E-01	4.883E-03	8.445E-03
492	wtCFTR and deltaF508 traffic / Membrane expression (normal and CF)	4.883E-03	8.445E-03	1.160E-01	2.125E-01	4.883E-03	8.445E-03
493	NETosis in SLE	4.926E-03	8.487E-03	1.850E-01	2.996E-01	4.926E-03	8.487E-03
494	Muscle contraction_GPCRs in the regulation of smooth muscle tone	5.044E-03	8.668E-03	6.263E-01	6.811E-01	5.044E-03	8.668E-03
495	Complement pathway disruption in thrombotic microangiopathy	5.059E-03	8.668E-03	7.553E-02	1.583E-01	5.059E-03	8.668E-03
496	Platelet activation as a result of endothelial dysfunction after stenting	5.190E-03	8.860E-03	1.691E-01	2.795E-01	5.190E-03	8.860E-03
497	Impaired inhibitory action of lipoxins on neutrophil migration in CF	5.190E-03	8.860E-03	1.345E-02	4.439E-02	5.190E-03	8.860E-03
498	Modification and recycling of the GABA-A and B receptors_Simona	5.278E-03	8.995E-03	8.246E-02	1.703E-01	5.278E-03	8.995E-03
499	Development_Dopamine D2 receptor transactivation of EGFR	5.356E-03	9.109E-03	1.229E-01	2.208E-01	5.356E-03	9.109E-03
500	Development_Role of Thyroid hormone in regulation of oligodendrocyte differentiation	5.644E-03	9.564E-03	3.447E-01	4.631E-01	5.644E-03	9.564E-03
501	DNA damage_ATM/ATR regulation of G1/S checkpoint	5.930E-03	1.002E-02	5.609E-01	6.403E-01	5.930E-03	1.002E-02
502	Reproduction_Progesterone-mediated oocyte maturation	5.943E-03	1.002E-02	2.695E-01	3.888E-01	5.943E-03	1.002E-02
503	Development_Transcription factors in segregation of hepatocytic lineage	6.590E-03	2.551E-02	6.590E-03	2.551E-02	1.649E-02	2.583E-02
504	Apoptosis and survival_Role of nuclear PI3K in NGF/ TrkA signaling	6.635E-03	1.112E-02	1.314E-01	2.304E-01	6.635E-03	1.112E-02
505	G-protein signaling_K-RAS regulation pathway	6.635E-03	1.112E-02	4.742E-01	5.713E-01	6.635E-03	1.112E-02
506	Apoptosis and survival_Apoptotic Activin A signaling	6.635E-03	1.112E-02	2.455E-02	7.008E-02	6.635E-03	1.112E-02
507	Autophagy_Autophagy	7.080E-03	1.183E-02	9.274E-03	3.421E-02	7.080E-03	1.183E-02
508	Nicotine signaling in chromaffin cells	8.057E-03	1.341E-02	2.884E-01	4.101E-01	8.057E-03	1.341E-02
509	Translation_Insulin regulation of translation	8.057E-03	1.341E-02	2.126E-02	6.321E-02	8.057E-03	1.341E-02
510	Development_NOTCH1-mediated pathway for NF-KB activity modulation	8.123E-03	1.347E-02	1.401E-01	2.418E-01	8.123E-03	1.347E-02
511	Development_HGF-dependent inhibition of TGF-beta-induced EMT	8.387E-03	1.386E-02	2.129E-01	3.277E-01	8.387E-03	1.386E-02
512	Oxidative stress_Role of ASK1 under oxidative stress	8.387E-03	1.386E-02	1.031E-02	3.647E-02	8.387E-03	1.386E-02
513	Signal transduction_PKA signaling	8.483E-03	1.397E-02	1.388E-01	2.412E-01	8.483E-03	1.397E-02

514	Development_Oligodendrocyte differentiation from adult stem cells	8.483E-03	1.397E-02	3.723E-01	4.852E-01	8.483E-03	1.397E-02
515	Oxidative stress_Role of Sirtuin1 and PGC1-alpha in activation of antioxidant defense system	8.532E-03	1.400E-02	4.520E-01	5.584E-01	8.532E-03	1.400E-02
516	Development_Regulation of endothelial progenitor cell differentiation from adult stem cells	8.532E-03	1.400E-02	4.520E-01	5.584E-01	8.532E-03	1.400E-02
517	Development_Alpha-1 adrenergic receptors signaling via Cyclic AMP	8.645E-03	1.408E-02	1.156E-02	3.979E-02	8.645E-03	1.408E-02
518	DNA damage_NHEJ mechanisms of DSBs repair	8.645E-03	1.408E-02	3.864E-01	4.998E-01	8.645E-03	1.408E-02
519	Development_NOTCH-induced EMT	8.645E-03	1.408E-02	8.255E-02	1.703E-01	8.645E-03	1.408E-02
520	Neurophysiological process_GABA-A receptor life cycle	9.836E-03	1.593E-02	3.010E-02	8.200E-02	9.836E-03	1.593E-02
521	Action of lithium on synaptic transmission and autophagy	9.863E-03	1.593E-02	5.936E-01	6.565E-01	9.863E-03	1.593E-02
522	Muscle contraction_Oxytocin signaling in uterus and mammary gland	1.073E-02	1.729E-02	4.689E-01	5.712E-01	1.073E-02	1.729E-02
523	TLRs-mediated IFN-alpha production by plasmacytoid dendritic cells in SLE	1.074E-02	3.734E-02	1.074E-02	3.734E-02	1.670E-01	2.195E-01
524	Development_WNT signaling pathway. Part 2	1.074E-02	3.734E-02	1.074E-02	3.734E-02	7.655E-02	1.053E-01
525	Regulation of lipid metabolism_Alpha-1 adrenergic receptors signaling via arachidonic acid	1.141E-02	1.831E-02	8.436E-01	8.579E-01	1.141E-02	1.831E-02
526	Development_SSTR2 in regulation of cell proliferation	1.152E-02	1.841E-02	2.317E-01	3.478E-01	1.152E-02	1.841E-02
527	G-protein signaling_G-Protein alpha-s signaling cascades	1.152E-02	1.841E-02	2.317E-01	3.478E-01	1.152E-02	1.841E-02
528	Immune response_IL-6-induced acute-phase response in hepatocytes	1.258E-02	4.206E-02	1.258E-02	4.206E-02	3.791E-02	5.552E-02
529	Development_Delta-type opioid receptor mediated cardioprotection	1.337E-02	2.133E-02	6.140E-01	6.743E-01	1.337E-02	2.133E-02
530	Cell cycle_Role of SCF complex in cell cycle regulation	1.401E-02	2.227E-02	5.256E-01	6.172E-01	1.401E-02	2.227E-02
531	Cell cycle_Role of SCF complex in cell cycle regulation	1.401E-02	2.227E-02	5.256E-01	6.172E-01	1.401E-02	2.227E-02
532	Development_Role of G-CSF in hematopoietic stem cell mobilization	1.529E-02	4.921E-02	1.529E-02	4.921E-02	1.806E-01	2.364E-01
533	Transport_ACM3 signaling in lacrimal glands	1.529E-02	2.428E-02	5.709E-01	6.446E-01	1.529E-02	2.428E-02
534	Cytoskeleton remodeling_ACM3 and ACM4 in keratinocyte migration	1.542E-02	2.439E-02	6.238E-01	6.804E-01	1.542E-02	2.439E-02
535	Regulation of lipid metabolism_Insulin regulation of glycogen metabolism	1.546E-02	2.441E-02	1.691E-01	2.795E-01	1.546E-02	2.441E-02
536	Immune response_T regulatory cell-mediated modulation of antigen-presenting cell functions	1.636E-02	2.580E-02	8.613E-02	1.758E-01	1.636E-02	2.580E-02
537	Development_S1P4 receptor signaling pathway	1.643E-02	2.583E-02	4.320E-01	5.405E-01	1.643E-02	2.583E-02
538	Transcription_Ligand-dependent activation of the ESR1/SP pathway	1.649E-02	2.583E-02	5.377E-01	6.229E-01	1.649E-02	2.583E-02
539	Translation _Regulation of EIF2 activity	1.769E-02	2.765E-02	7.553E-02	1.583E-01	1.769E-02	2.765E-02
540	Apoptosis and survival_DNA-damage-induced apoptosis	1.778E-02	2.770E-02	3.199E-01	4.381E-01	1.778E-02	2.770E-02
541	Development_Astrocyte differentiation from adult stem cells	1.805E-02	5.602E-02	1.805E-02	5.602E-02	5.907E-02	8.272E-02
542	Development_ERK5 in cell proliferation and neuronal survival	1.962E-02	5.947E-02	1.962E-02	5.947E-02	7.405E-02	1.020E-01
543	Apoptosis and survival_Beta-2 adrenergic receptor anti-apoptotic action	1.981E-02	3.072E-02	4.464E-01	5.550E-01	1.981E-02	3.072E-02
544	wtCFTR traffic / Sorting endosome formation (normal)	1.981E-02	3.072E-02	4.464E-01	5.550E-01	1.981E-02	3.072E-02
545	DNA damage_DNA-damage-induced responses	2.117E-02	3.265E-02	2.064E-01	3.235E-01	2.117E-02	3.265E-02
546	Transcription_Mechanism of activation of the transcription of Retinoid-target genes	2.201E-02	6.399E-02	2.201E-02	6.399E-02	5.115E-01	6.079E-01
547	wtCFTR and deltaF508 traffic / Late endosome and lysosome (normal and CF)	2.242E-02	3.446E-02	6.085E-02	1.359E-01	2.242E-02	3.446E-02
548	Immune response_MIF-JAB1 signaling	2.362E-02	3.601E-02	1.229E-01	2.208E-01	2.362E-02	3.601E-02
549	Development_Mu-type opioid receptor signaling via Beta-arrestin	2.362E-02	3.601E-02	4.605E-01	5.635E-01	2.362E-02	3.601E-02
550	CFTR folding and maturation (normal and CF)	2.362E-02	3.601E-02	1.229E-01	2.208E-01	2.362E-02	3.601E-02
551	RHS_pathways	2.520E-02	7.166E-02	2.520E-02	7.166E-02	1.530E-01	2.030E-01
552	Cell adhesion_IL-8-dependent cell migration and adhesion	2.574E-02	3.918E-02	2.036E-01	3.196E-01	2.574E-02	3.918E-02
553	Development_S1P2 and S1P3 receptors in cell proliferation and differentiation	2.725E-02	7.556E-02	2.725E-02	7.556E-02	1.062E-01	1.424E-01
554	Cell adhesion_Endothelial cell contacts by junctional mechanisms	2.725E-02	7.556E-02	2.725E-02	7.556E-02	2.755E-01	3.480E-01
555	LRRK2 in neuronal apoptosis in Parkinson's disease	2.772E-02	4.183E-02	3.540E-01	4.692E-01	2.772E-02	4.183E-02
556	Transcription_Transcription factor Tubby signaling pathways	2.772E-02	4.183E-02	3.540E-01	4.692E-01	2.772E-02	4.183E-02

557	Neurophysiological process_Synaptic vesicle fusion and recycling in nerve terminals	2.775E-02	4.183E-02	3.814E-01	4.954E-01	2.775E-02	4.183E-02
558	Immune response_T cell subsets: cell surface markers	2.775E-02	4.183E-02	7.378E-01	7.714E-01	2.775E-02	4.183E-02
559	Cytoskeleton remodeling_Neurofilaments	2.787E-02	4.193E-02	1.314E-01	2.304E-01	2.787E-02	4.193E-02
560	Transport_cAMP/ Ca(2+)-dependent Insulin secretion	2.910E-02	4.372E-02	6.693E-01	7.111E-01	2.910E-02	4.372E-02
561	Chemotaxis_CCR4-induced chemotaxis of immune cells	2.946E-02	4.405E-02	5.830E-01	6.494E-01	2.946E-02	4.405E-02
562	Mechanism of action of CCR4 antagonists in asthma and atopic dermatitis (Variant 1)	2.946E-02	4.405E-02	5.830E-01	6.494E-01	2.946E-02	4.405E-02
563	CCR4-dependent immune cell chemotaxis in asthma and atopic dermatitis	2.946E-02	4.405E-02	5.830E-01	6.494E-01	2.946E-02	4.405E-02
564	ENaC regulation in normal and CF airways	3.075E-02	4.590E-02	1.507E-01	2.567E-01	3.075E-02	4.590E-02
565	Cell adhesion_Plasmin signaling	3.351E-02	4.963E-02	2.223E-01	3.382E-01	3.351E-02	4.963E-02
566	Development_Regulation of telomere length and cellular immortalization	3.351E-02	4.963E-02	5.817E-02	1.315E-01	3.351E-02	4.963E-02
567	Neurophysiological process_EphB receptors in dendritic spine morphogenesis and synaptogenesis	3.351E-02	4.963E-02	5.817E-02	1.315E-01	3.351E-02	4.963E-02
568	Beta-adrenergic-dependent CFTR expression	3.371E-02	4.984E-02	3.704E-01	4.835E-01	3.371E-02	4.984E-02
569	Transport_Macropinocytosis	3.575E-02	9.136E-02	3.575E-02	9.136E-02	2.050E-01	2.632E-01
570	Epigenetics_Default	3.662E-02	5.388E-02	2.962E-01	4.182E-01	3.662E-02	5.388E-02
571	Set A_deregulated_mechanisms	3.953E-02	9.814E-02	3.953E-02	9.814E-02	8.884E-01	9.655E-01
572	Schema: Initiation of T cell recruitment in allergic contact dermatitis	4.040E-02	5.907E-02	8.255E-02	1.703E-01	4.040E-02	5.907E-02
573	Muscle contraction_Regulation of eNOS activity in cardiomyocytes	4.104E-02	5.992E-02	4.172E-01	5.271E-01	4.104E-02	5.992E-02
574	Development_Retinoic acid and retinoic acid receptors in regulation of oligodendrocyte differentiation	4.266E-02	6.199E-02	2.411E-01	3.585E-01	4.266E-02	6.199E-02
575	Development_MAG-dependent inhibition of neurite outgrowth	4.266E-02	6.199E-02	2.411E-01	3.585E-01	4.266E-02	6.199E-02
576	Development_Beta-adrenergic receptors transactivation of EGFR	4.266E-02	6.199E-02	6.659E-02	1.450E-01	4.266E-02	6.199E-02
577	DeltaF508-CFTR traffic / Sorting endosome formation in CF	4.337E-02	6.272E-02	5.133E-01	6.081E-01	4.337E-02	6.272E-02
578	Possible influence of low doses of Arsenite on glucose uptake in muscle	4.337E-02	6.272E-02	5.133E-01	6.081E-01	4.337E-02	6.272E-02
579	Neurophysiological process_Dopamine D2 receptor signaling in CNS	4.466E-02	6.428E-02	7.017E-01	7.376E-01	4.466E-02	6.428E-02
580	Mechanisms of deltaF508 CFTR activation by S-nitrosoglutathione	4.466E-02	6.428E-02	3.354E-01	4.561E-01	4.466E-02	6.428E-02
581	Development_Role of nicotinamide in G-CSF-induced granulopoiesis	4.735E-02	6.795E-02	2.653E-01	3.850E-01	4.735E-02	6.795E-02
582	wtCFTR and deltaF508-CFTR traffic / Generic schema (normal and CF)	4.777E-02	6.825E-02	2.506E-01	3.676E-01	4.777E-02	6.825E-02
583	Regulation of degradation of wtCFTR	4.778E-02	6.825E-02	4.020E-01	5.145E-01	4.778E-02	6.825E-02
584	Cytoskeleton remodeling_Role of Activin A in cytoskeleton remodeling	4.778E-02	6.825E-02	4.020E-01	5.145E-01	4.778E-02	6.825E-02
585	Development_SSTR1 in regulation of cell proliferation and migration	4.949E-02	7.058E-02	5.256E-01	6.172E-01	4.949E-02	7.058E-02
